# Supramolecular Recognition of Cytidine Phosphate in Nucleotides and RNA Sequences

**DOI:** 10.1021/jacsau.2c00658

**Published:** 2023-02-13

**Authors:** Boris S. Morozov, Aleksandr S. Oshchepkov, Insa Klemt, Aleksandr M. Agafontsev, Swathi Krishna, Frank Hampel, Hong-Gui Xu, Andriy Mokhir, Dirk Guldi, Evgeny Kataev

**Affiliations:** † Department of Chemistry and Pharmacy, Friedrich-Alexander-Universität Erlangen-Nürnberg, Nikolaus-Fiebiger-Str. 10, Erlangen 91058, Germany; ‡ Max-Planck-Institut für die Physik des Lichts, Staudtstraße 2, Erlangen 91058, Germany; § Department of Chemistry and Pharmacy, Interdisciplinary Center for Molecular Materials (ICMM), Friedrich-Alexander-Universität Erlangen-Nürnberg, Egerlandstr. 3, Erlangen 91058, Germany

**Keywords:** synthetic receptors, fluorescence sensing, nucleotide recognition, cytosine, encapsulation

## Abstract

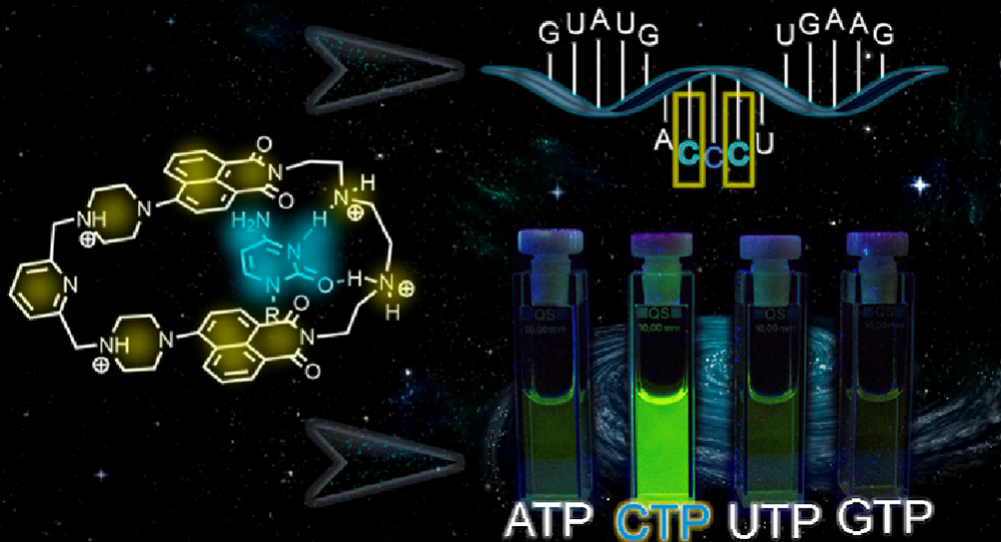

Supramolecular recognition of nucleotides would enable manipulating crucial biochemical pathways like transcription and translation directly and with high precision. Therefore, it offers great promise in medicinal applications, not least in treating cancer or viral infections. This work presents a universal supramolecular approach to target nucleoside phosphates in nucleotides and RNA. The artificial active site in new receptors simultaneously realizes several binding and sensing mechanisms: encapsulation of a nucleobase via dispersion and hydrogen bonding interactions, recognition of the phosphate residue, and a self-reporting feature—“turn-on” fluorescence. Key to the high selectivity is the conscious separation of phosphate- and nucleobase-binding sites by introducing specific spacers in the receptor structure. We have tuned the spacers to achieve high binding affinity and selectivity for cytidine 5′ triphosphate coupled to a record 60-fold fluorescence enhancement. The resulting structures are also the first functional models of poly(rC)-binding protein coordinating specifically to C-rich RNA oligomers, e.g., the 5′-AUCCC(C/U) sequence present in poliovirus type 1 and the human transcriptome. The receptors bind to RNA in human ovarian cells A2780, causing strong cytotoxicity at 800 nM. The performance, self-reporting property, and tunability of our approach open up a promising and unique avenue for sequence-specific RNA binding in cells by using low-molecular-weight artificial receptors.

## Introduction

Supramolecular targeting RNA with small molecules represents a crucial challenge in reading genetic information and controlling replication.^1^ RNA is known to adopt various conformations such as hairpin loops, bulges, etc.^1,2^ Drugs with specific site selectivity would be an excellent solution to treat many diseases such as viral infections and cancer. Antisense oligonucleotides and small interfering RNAs (siRNAs) represent attractive classes of compounds with high RNA specificity. However, their delivery to organs other than the liver remains a significant problem.^3^ Thus, synthetic receptors with high nucleobase selectivity may provide a novel approach to target RNA sequences.

The field of supramolecular recognition of nucleotides has recently attracted much attention.^4−14^ Recent studies have demonstrated the ability of synthetic receptors to detect abasic sites,^15^ short DNA^16^ and RNA^17−20^ sequences, and mismatched base pairs.^21,22^ The fluorescence properties of receptors deliver additional advantages to detect the interactions by imaging.^23−27^ In designing fluorescent probes for nucleotides, dyes are used as molecular building blocks interacting with nucleobases.^28^ Several sensing mechanisms for the detection of nucleotides in water are known to this date, such as receptors based on anthracene- or pyrene excimer–monomer equilibrium,^29−33^ indicator-displacement assay,^34^ PET probes,^31,32,35−42^ Förster resonance energy transfer (FRET),^43^ aggregation-induced emission (AIE), aggregation-induced quenching (AIQ),^44−50^ as well as transition metal and lanthanide complexes.^51−59^

However, the major obstacle to achieve high binding selectivity for nucleotides is to develop the receptor design with tunable binding sites enabling the control over affinity and selectivity for a specific nucleobase. A prerequisite to broad applicability of such receptors is their solubility in an aqueous buffered solution at close to neutral pH.

**Figure 1:**
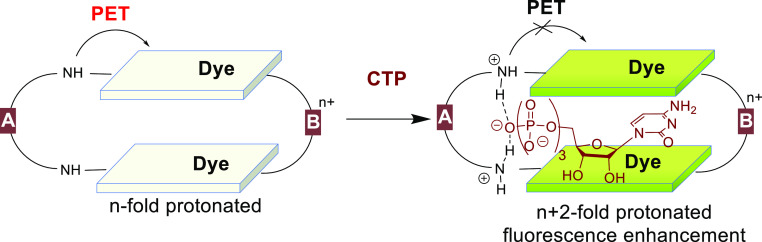
Design and sensing mechanism for detecting cytidine phosphates realized in this work.

In this work, we address these challenges by introducing an effective supramolecular approach to target nucleoside phosphates in nucleotides and RNA. A novel and universal design involves receptors with two dyes combining different *A* and *B spacers* (Figure 1). We have proposed that such an approach will allow us to separate binding sites for the phosphate residue and for the nucleobase. The phosphate-selective recognition unit (*spacer A*) is coupled to dyes in a way to function at the same time as a PET-reporter of the binding process. *Spacer B* bears a hydrogen bonding pattern to recognize nucleobases. We have shown that fine-tuning *spacers A* and *B* dramatically affects the selectivity and fluorescence response of the respective receptors.

To investigate the applicability of our approach, we focused on cytidine phosphate recognition, as none of the known receptors possess sufficient selectivity and affinity.^59,60^ Among the five natural nucleobases, cytosine (C) is the most challenging and important target. There is a cogent need to bind and detect cofactor cytidine triphosphate (CTP), trinucleotide repeat expansion diseases,^61^ and 3′-desoxy-3′,4′-didehydro-cytidine (ddhCTP).^62^ Notable is also the fact that cytosine-rich DNA sequences are likely to form four-stranded structures, which are functionally important parts of the genome: promoters of genes and telomeres.^63,64^ Cytosine-rich RNA bulges are essential parts of enteroviruses and are important for the efficient translation of the viral mRNA.^65^

Using the proposed approach, we synthesized a new family of receptors and assayed with nucleoside phosphates in aqueous buffered solutions. The applicability of new receptors was investigated in the recognition and detection of cytidine phosphates in nucleotides, RNA sequences, and in cancer cells. The introduction of 2,5-pyridine and 1,3-benzene spacers (*spacer A*) and an ethylene diamine spacer (*spacer B*) resulted in receptor **6**, which showed strong fluorescence enhancement in the presence of CTP, while other triphosphates induce negligible changes, namely, < twofold. According to the experiments with human ovarian cancer A2780 cells, our new receptors bind specifically to RNA rather than to free cytidine phosphates and show micromolar cytotoxicity. Our work demonstrates the success in rationally designing receptors, which detect cytidine phosphate and C-rich RNA oligos with high selectivity. In particular, **6** can bind and detect 5′-AUCCC(C) sequences present in poliovirus type 1 (PV1). This sequence is essential for efficient translation of the viral mRNA. Thus, **6** can be considered as the first functional model of poly(rC)-binding protein, known to show high affinity for poly(C) in a sequence-specific manner.^66^ Our results set the stage for developing new water-soluble receptors as promising drug candidates functioning via sequence-selective RNA recognition.

## Results and Discussion

### Design, Synthesis, and Structure of Receptors

Recent investigations with cyclophanes^33,67−70^ and tweezer-like^71^ receptors have demonstrated their ability to bind aromatics even in highly competitive aqueous solutions. The use of naphthalimides in the molecular recognition of nucleic acids inspired us to incorporate them into the new receptor design. By virtue of the high sensitivity of piperazine-functionalized naphthalimides to a protonation equilibrium, we opted for a new family of receptors featuring different spacers, which enable the control over fine-tuning the noncovalent interactions in the receptor–nucleotide complex. According to Figure 1, *spacer A* (pyridine and *p*- and *m*-phenylene) was used to tune the basicity and rigidity of the piperazine nitrogen atoms that coordinate the phosphate residue and produce a strong fluorescence response. Flexible aliphatic *spacer B* connects two dyes and carries hydrogen bond donor (NH) or acceptor (O) sites. The latter is responsible for nucleobase recognition. The particular feature of cytosine that we utilize to discriminate it from other bases is the ability of cytosine to be protonated at the N^3^ position and thus form complementary hydrogen bonding interactions with *spacer B*. For instance, protonated cytosine is observed in the i-motif containing C–C^+^ base pairs.^72^ In a nutshell, the complexation of CTP or C-rich RNA should lead to the protonation of *spacer A*, formation of hydrogen bonds between cytosine and *spacer B*, and as a result, induce a “turn-on” response.

**Figure 2:**
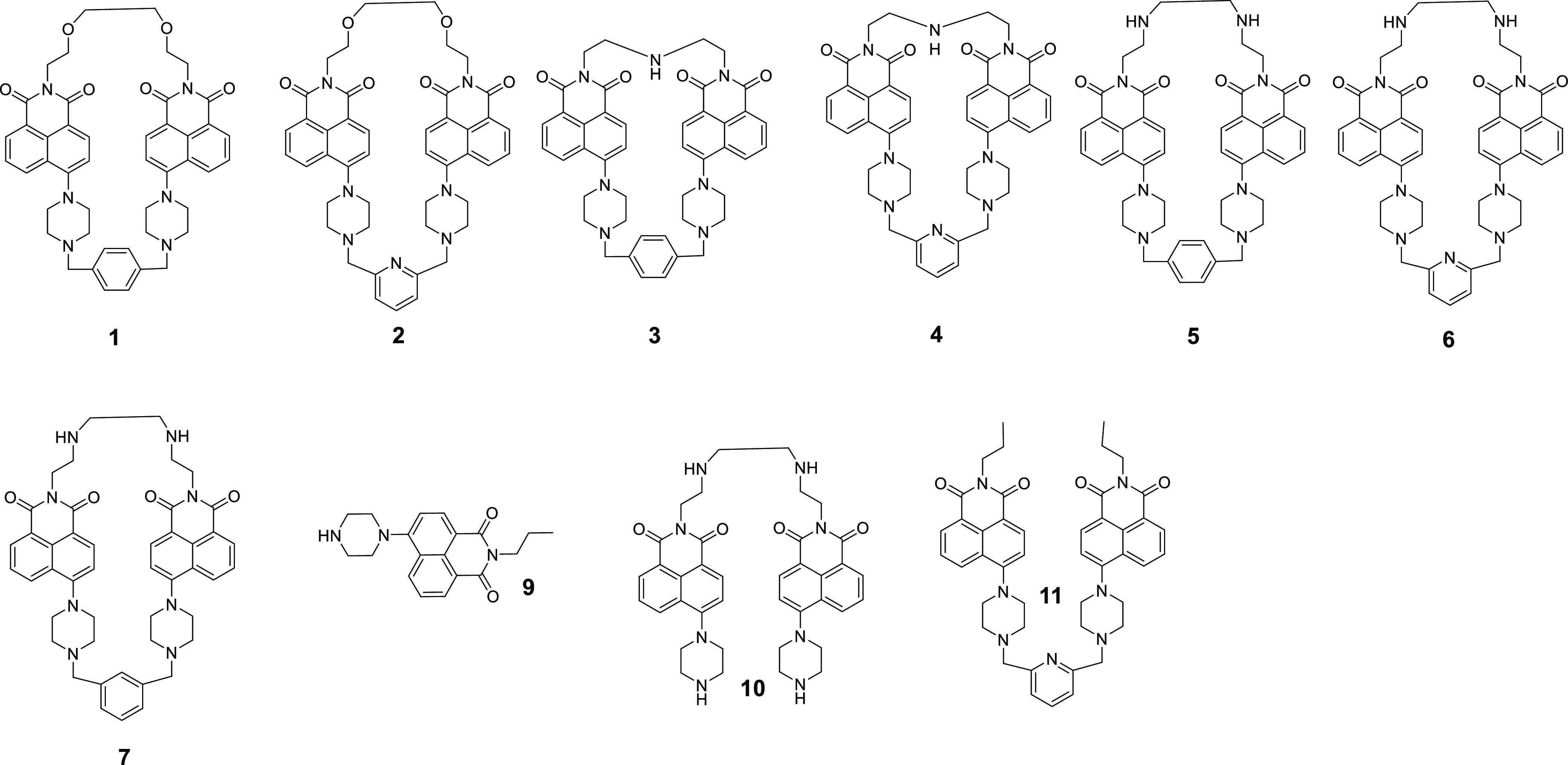
Structures of receptors **1–7** and reference compounds used in the binding studies **9–11**.

**Scheme 1:**
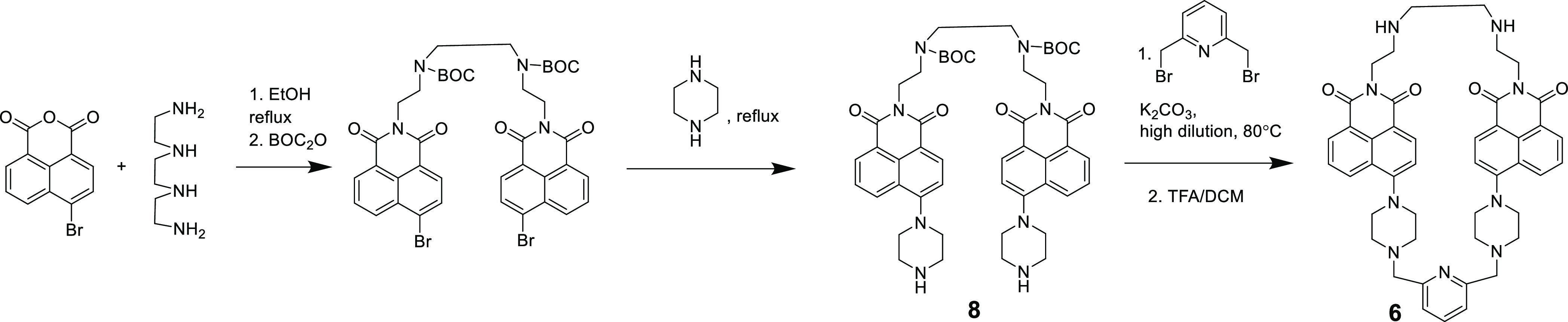
Synthesis of Receptor **6** through Intermediate **8** BOC – *tert*-butyloxycarbonyl, TFA – trifluoroacetic acid, and DCM – dichloromethane.

Receptors were synthesized by using the general strategy shown in Scheme 1 for receptor **6**. 4-Bromo-1,8-naphthalic anhydride was reacted with the corresponding diamine to form, e.g., bisnaphthalimide building blocks, which were then converted to **8**. Alkylation by dibromides under high dilution conditions led to the formation of the receptor with an average yield of 30%. The products were deprotected to form the corresponding receptor quantitatively. Compounds **9**, **10**, and **11** were prepared as references for the binding assays with nucleotides (Figure 2).

**Figure 3:**
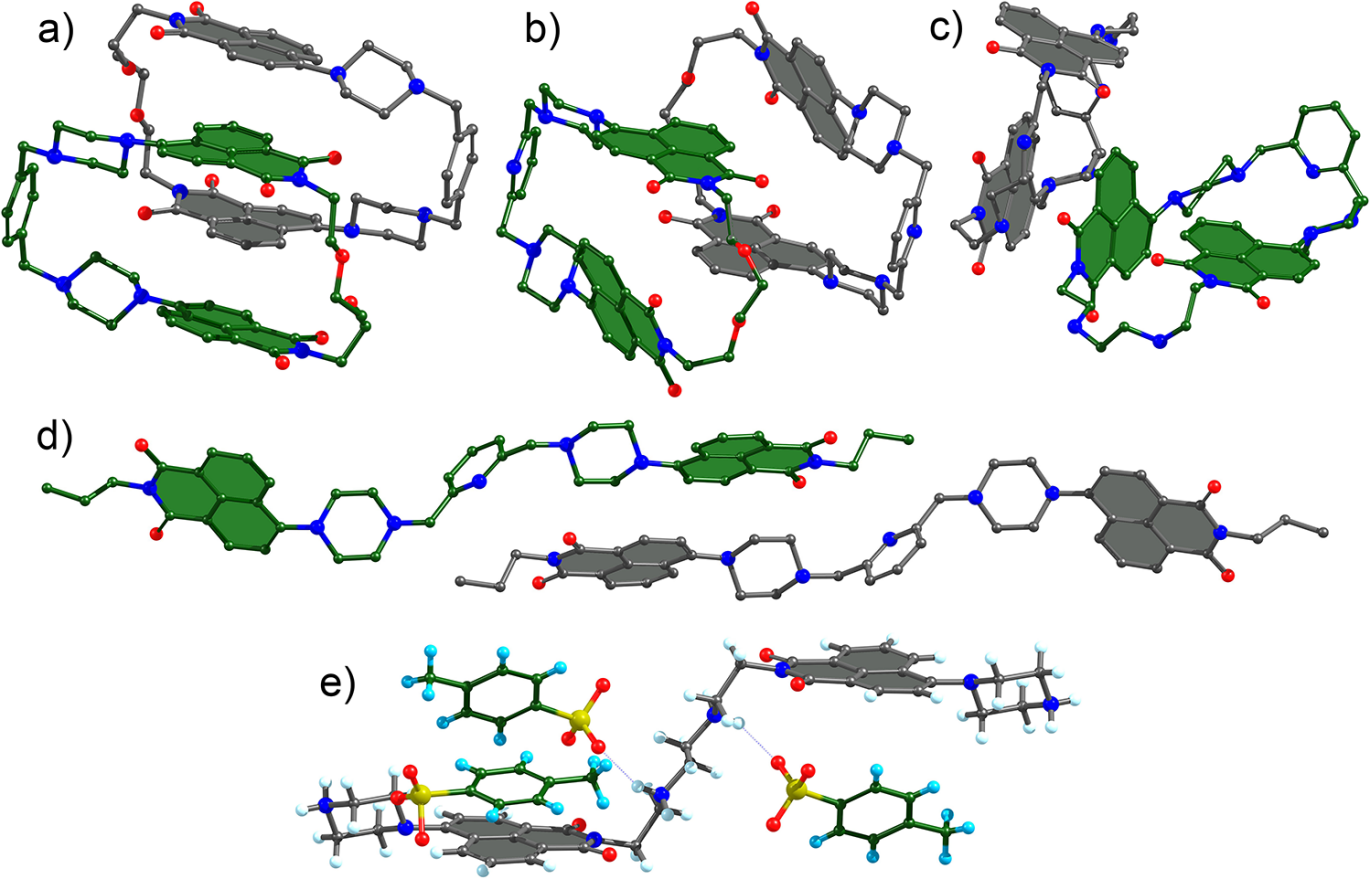
Molecular structures of receptors: (a) **1**(HCl)_2_, (b) **2**, (c) **6**, (d) **10**, and (e) **11**(TsOH)_4_ according to the single-crystal X-ray structure analysis. Solvent molecules, hydrogen, and chloride atoms are omitted for clarity. Structures of **1**, **2**, **3**, and **10** are presented in pairs with the closest molecules in the unit cells showing stacking interactions between naphthalimide rings. Compound **11** is shown together with tosylate (TsO^–^) anions forming noncovalent interactions with the receptor.

Single X-ray crystal structures were obtained for **1**·2HCl, **2**, **6**, **10**, and **11**(TsOH)_4_. According to the data, individual receptors interact with each other through naphthalimide–naphthalimide π–π or H−π interactions, depending on the receptor structure. Figure 3 shows parts of the unit cells and illustrates the different intermolecular interactions. For instance, **1** hosts naphthalimide rings, **2** and **6** also π–π interact with next neighbors. In the crystal structure of **11**, two receptors interact with each other through their naphthalimides. Naphthalimide–guest π–π or H−π interactions were also observed in the structure of the tosylate salt of **10** (Figure 3e). Observations in the solid-state provide solid evidence for our new receptors to complex aromatics between the naphthalimides via π–π and H−π interactions.

### Binding and Sensing of Nucleotides

All receptors were analyzed in aqueous solutions containing different portions of DMSO, namely, 5% DMSO—aqueous 50 mM MOPSO buffer solution—for UV–vis and fluorescence measurements and 10% DMSO for NMR assays. According to dilution experiments, the receptors lack any aggregation under the chosen conditions. Also, temperature-dependent absorption spectra fail to reveal any hyperchromic effects confirming the absence of any intermolecular aggregation.

**Figure 4:**
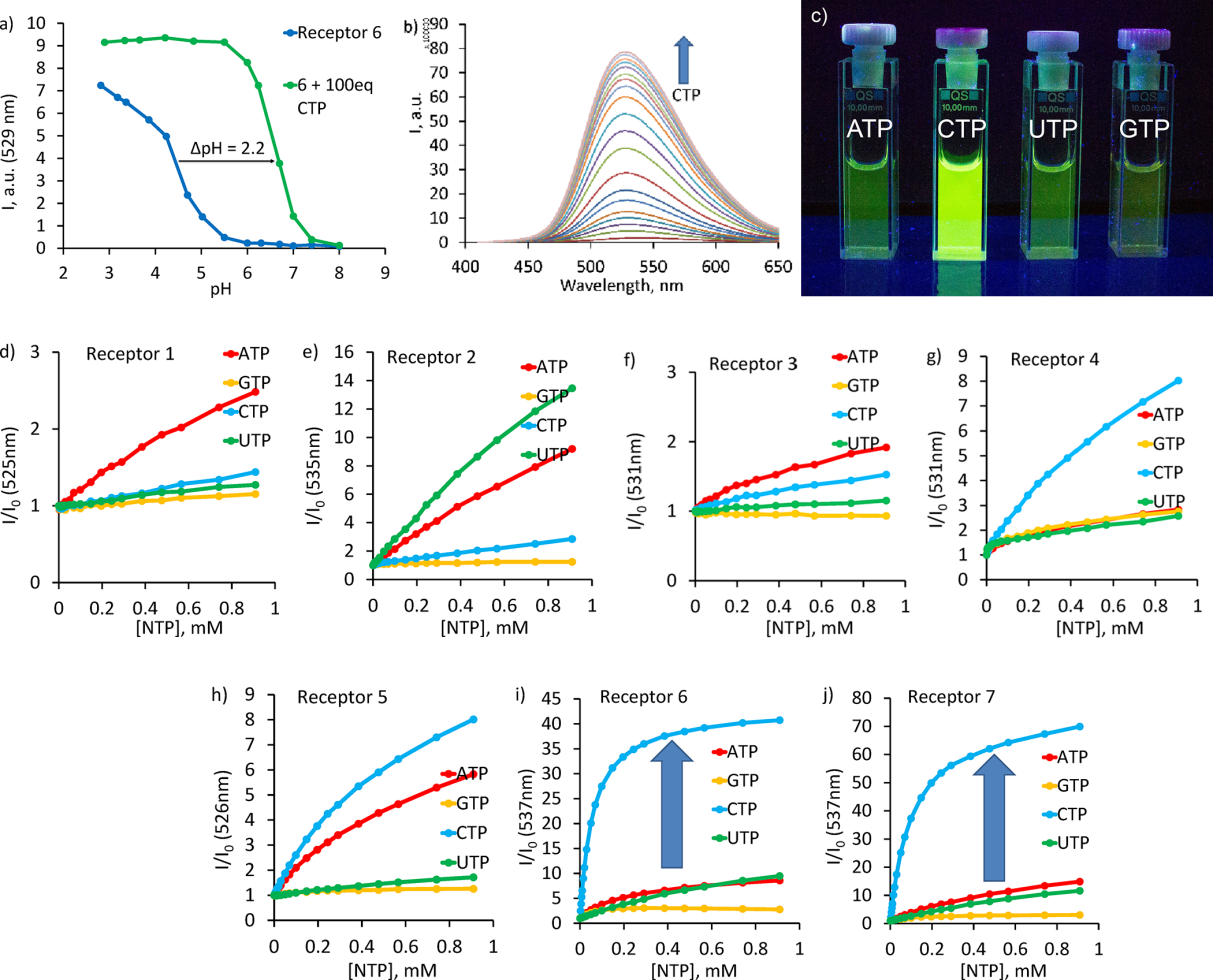
(a) Fluorescence intensity of **6** depending on pH and the presence of CTP. (b) Changes in the fluorescence spectra of **6** during the addition of CTP (ex. 400 nm). (c) Photograph of the solution of **6** in the presence of 10 equiv of four nucleoside triphosphates. (d–j) Fluorescence titrations of receptors **1–7** with nucleotides: 0.01 mM, 50 mM MOPSO buffer (5% DMSO, pH 6.2).

To find the optimum pH window for nucleotide detection, we measured the fluorescence as a function of pH in the absence and presence of CTP. As shown in Figure 3a, an excess of CTP results in a strong fluorescence increase. The strongest enhancement was observed for **5** (pH 4–6), **6**, and **7** (pH 5–7). Pyridine and 1,3-benzene render the receptor more basic and shift the enhancement into the neutral pH region. Considering the aforementioned facts, we selected pH 6.2 for the binding and sensing assays. In line with the UV–vis dilution experiments, the 10^–6^–10^–4^ M concentration range (Supporting Information (SI) Figure S20) lacks any intermolecular aggregation. Fluorescence titration experiments revealed that all triphosphates, that is, ATP, CTP, GTP, and UTP, induce a “turn-on” response. Addition of ATP and CTP resulted in the strongest fluorescence enhancement. Interestingly, even GTP shows in most cases, a small fluorescence enhancement. Such behavior was never seen before. As a matter of fact, guanine typically quenches the fluorescence of most dyes by either a static or a dynamic mechanism.^73^ As illustrated in Figure 4, the remarkable CTP selectivity in the fluorescence response appears only for **4**, **6**, and **7**. These three receptors have similar structural features, that is, polyamine (*spacer B*) and 2,5-pyridine or 1,3-benzene (*spacer A*). Receptors **6** and **7** outperform **4** in the overall fluorescence enhancement with *I*/*I*_0_ values of 40-, 50-fold, and 8-fold with 0.9 mM NTP, respectively. According to our measurements, the fluorescence quantum yield of **6** increases from 0.036 to 0.16 upon saturation with CTP. Receptor **7** has slightly better enhancement because it shows a lower quantum yield (0.01) in the free form.

All binding constants were obtained by fitting the data with the HypSpec program considering a 1:1 host–guest ratio, which was corroborated by the Job plot method and the fitting analysis.^74^ To confirm the above, we also conducted UV–vis titrations (Table 1). In general, receptors with ethylene glycol spacers show slightly lower binding constants of about 10^3^ M^–1^. This is due to the fact that at pH 6.2 they are less positively charged than those bearing ethylene diamine spacers. Receptor **6** shows the highest binding constant and selectivity for CTP with log *K* = 4.4. Additional evidence for the strong binding came from analyzing the ESI spectra in methanol–water mixtures. *m/z* values were in a good agreement with the predicted ones: *m/z* = 911.4092 corresponds to [**6**·Cytosine·Na^2+^]^+^ and *m/z* = 1343.2764 to [**6**·CTP^4–^·Na_2_^+^K^+^]^+^ (Figures S34 and S35). According to the fluorescence measurements, the limit of CTP detection is 250 nM (Figure S32). We also conducted competition experiments, in which we investigated the presence of competing nucleotides on the CTP detection in solution. As expected, GTP affects the overall enhancement after the addition of CTP, while ATP and UTP lack any considerable effects (Figure S31).

**Table 1:** Binding Constants (log *K*) Determined by Fluorescence and UV–vis Experiments: 0.01 mM Receptor in a 50 mM MOPSO Buffer (5% DMSO, pH 6.2)^a^

nucleotide/receptor	CTP	ATP	GTP	UTP
**1**	2.02	2.76	2.39	2.36
**2**	2.31	2.58	3.34	2.64
**3**	2.96	3.11	n.d./3.89b	2.88
**4**	2.93	3.28	3.50	3.15
**5**	3.78/3.80^b^	3.33/3.64^b^	3.43/3.62^b^	2.77/3.20^b^
**6**	4.43/4.38^b^	3.36/3.38^b^	3.40/3.54^b^	3.20/3.27^b^
**7**	3.98	3.08	3.93	2.84

a Binding constants were determined by fitting the experimental data of fluorescence measurements with a 1:1 stoichiometry binding model. Experimental errors do not exceed 5%.

b Binding constants were determined from UV–vis measurements.

### Binding and Sensing Mechanism

To gather additional insights into the binding and sensing mechanism, we focused on two questions. First, what is the origin of fluorescence enhancement induced by CTP? Second, what is the structure of the host–guest complexes?

To answer the first question, we investigated how the fluorescence of **6** depends on protonation. A total of four amine groups are protonable in water. p*K*_a_ values for **6** (corresponding to the conjugated acid) were determined by potentiometric titrations: 8.7, 6.8, 5.4, and 4.9. Our data analysis reveals that **6** is doubly protonated under the titration conditions, namely, pH 6.2 (Figure S19), and two positive charges are located at the secondary amines of *spacer B*. Without any CTP, the fluorescence grows stepwise from pH 6 to 3 (Figure 4a). This fact suggests that **6** accepts two additional protons at its piperazine sites. In the presence of CTP, the fluorescence grows, however, steeply and reaches the maximum at pH 6. The absence of a stepwise growth indicates that coordination of CTP requires simultaneous protonation of the piperazines. This fact was also confirmed by the potentiometric titration of **6** with CTP. The species distribution diagram (Figure S19) suggests the major species in solution under the titration conditions is the complex with composition **6**H_4_^4+^CTP^4–^. Upon lowering the pH, its concentration increases in line with the fluorescence increase detected in the presence of CTP (Figure 4a).

Next, we elucidated how macrocyclic effects influence the receptor selectivity by comparing the binding and sensing of cyclic receptors with those of references **9**, **10**, and **11**. Compounds **9** and **11** show weak responses, namely, a 5% fluorescence increase upon addition of ATP and CTP, and low binding constants of <100 M^–1^. For **10** however, a stronger fluorescence response and binding constants of log *K* 5.01 for GTP, 4.12 for ATP, and 3.92 for CTP were noted. Compound **10** is, therefore, selective for GTP and shows a 10-fold fluorescence quenching. According to the literature, quenching by GTP is operative when receptors are subject to π–π interactions.^28^ The fluorescence of **10** is quenched by GTP, while **6** shows a weak fluorescence enhancement. We postulate that guanine is bound to the receptors in different modes. Both amine and carbonyl groups of the cytosine and guanine nucleobases are likely to be responsible for hydrogen bond formation. As such, the binding modes of these nucleotides with **6** are thought to be similar. Based on comparing the binding data for **6** and the references, we suggest that the ethylenediamine linker is essential to achieve high affinity and selectivity for CTP.

**Figure 5:**
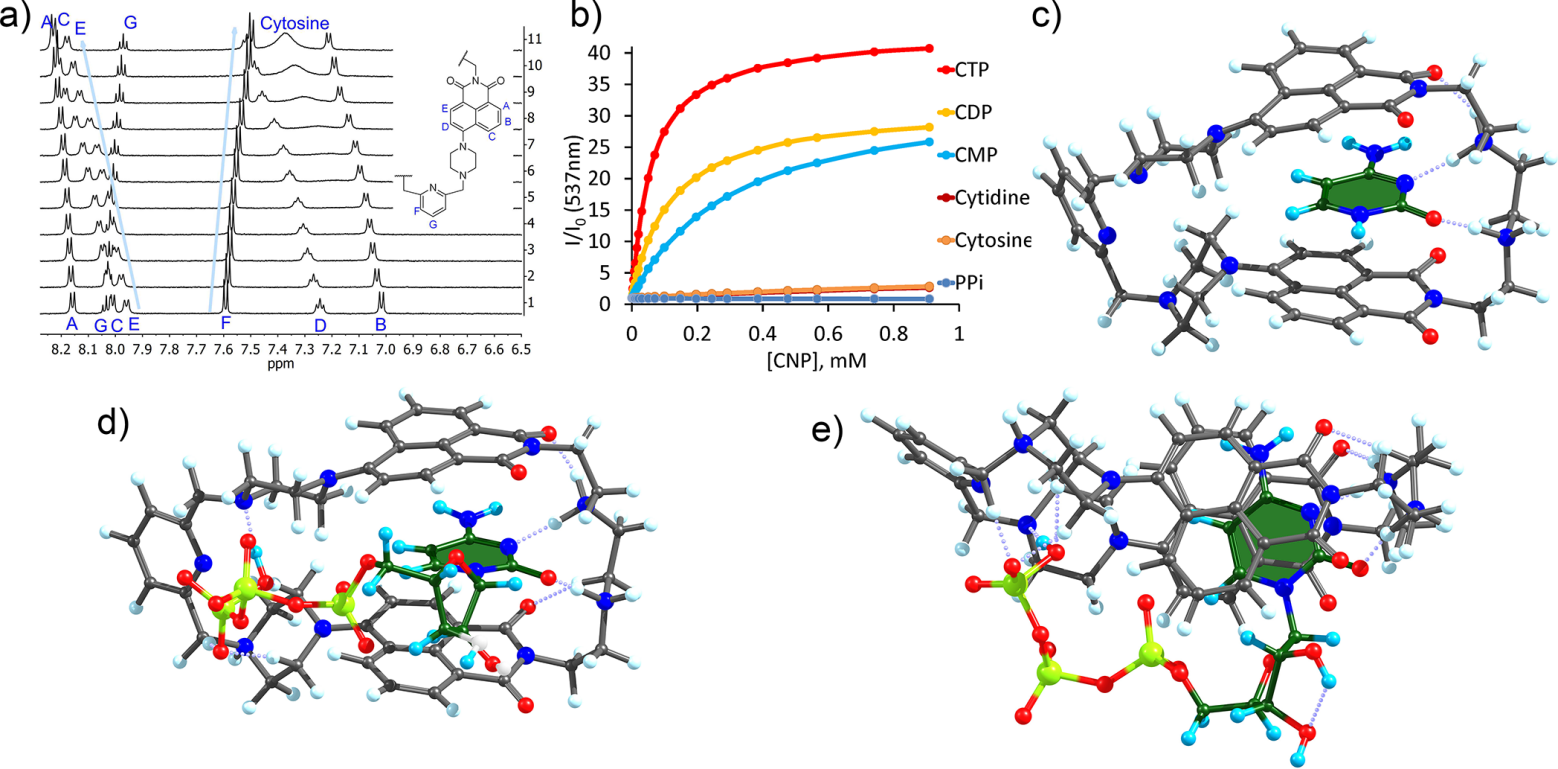
(a) ^1^H NMR titration of **6** with cytosine: 50 mM MOPSO buffer in D_2_O (pH 6.2. 10% DMSO-*d6*). (b) Fluorescence titration of **6** with different cytosine derivatives and pyrophosphate (PPi). DFT optimized complexes **6**H_2_^2+^·cytosine (c) and **6**H_4_^4+^·CTP^4–^ front and side views (d, e).

To understand the contributions stemming from the electrostatic interactions between protonated amines and phosphates, we determined the binding constants for mono-, di-, and triphosphates, including the pyrophosphate anion. Fluorescence and ^1^H NMR titrations were carried out to determine the binding constants. The results from both methods were in good agreement with each other. According to Table 2, the affinity of **6** drops CTP > CDP > CMP > cytosine > cytidine. Even cytosine was found to bind to **6** with relatively good affinity in aqueous solutions. According to the ^1^H NMR titrations (Figure 5a), the addition of cytosine leads to down-field shifted naphthalimide signals. Also, the signals of cytosine shift next to a broadening. Cytosine induces a small fluorescence enhancement, while pyrophosphate lacks any notable effects. The overall enhancement grows as the length of the phosphate residue is increased: triphosphate > diphosphate > monophosphate (Figure 5b). Longer phosphates provide stronger electrostatic interactions and more efficiently induce protonation of the piperazine subunit, which in turn leads to stronger fluorescence enhancement. Thus, the length of a nucleotide is important for a strong analytical answer. We also titrated **6** with CTP in the solutions of higher ionic strength by using a buffer with 1 M NaCl. Here, the binding constant of log *K* = 2.69 is similar to that of the value found for cytidine. Our results confirm that electrostatic interactions contribute more than one order of magnitude to the overall binding of cytidine phosphates.

**Table 2:** Binding Constants (log *K/*Fluorescence Enhancement, *I*/*I*_0_) for Receptor **6** Obtained by Using Fluorescence and ^1^H NMR Titration with Cytosine Derivatives

method	CDP	CMP	cytidine	cytosine	PPi
fluorescence	3.93/28 fold	3.49/25 fold	2.62/2.6 fold	2.80/2.8 fold	a
^1^H NMR	b	3.32	2.68	2.88	b

a The changes in fluorescence intensity were not detected.

b Precipitation occurs.

Considering that our binding experiments reveal the complexation of cytosine by **6**, we looked into possible coordination modes of cytosine in the cavity of twofold protonated **6** by using a parametrized model involving dispersion interactions.^75,76^ All structures were further optimized by using ORCA software to find the most favorable geometry. The calculations were performed at the BP86-def2-TZVP level of theory with a D3 model for dispersion corrections.^77^ As it is seen in Figure 5c, cytosine is located between two naphthalimides and forms two hydrogen bonds with the protonated amines of *spacer B*. The distances between the stacked π-systems are in the expected range of 3.1–3.2 Å. The ammonium sites serve as hydrogen bond donors to coordinate cytosine. In the optimized neutral structure, **6**H_4_^4+^·CTP^4–^, the conformation of **6** is almost identical to that observed in the crystal structure. Triphosphate locates from the outside of the cycle forming hydrogen bonding interactions with the protonated piperazines (Figure 5d,e). The latter structure shows a perfect match of the macrocycles’ length and the size of CTP.

^1^H-^1^H ROESY NMR data of the host–guest complex with CMP supported the proposed position of cytosine close to *spacer B*. In this experiment, we mixed CMP with **6** in a methanol–water mixture rather than CTP, because triphosphates form insoluble complexes at the NMR concentrations. NOE signals were detected between the CH_2_ groups of *spacer B* and the protons of the pentose ring (Figure S36).

**Figure 6:**
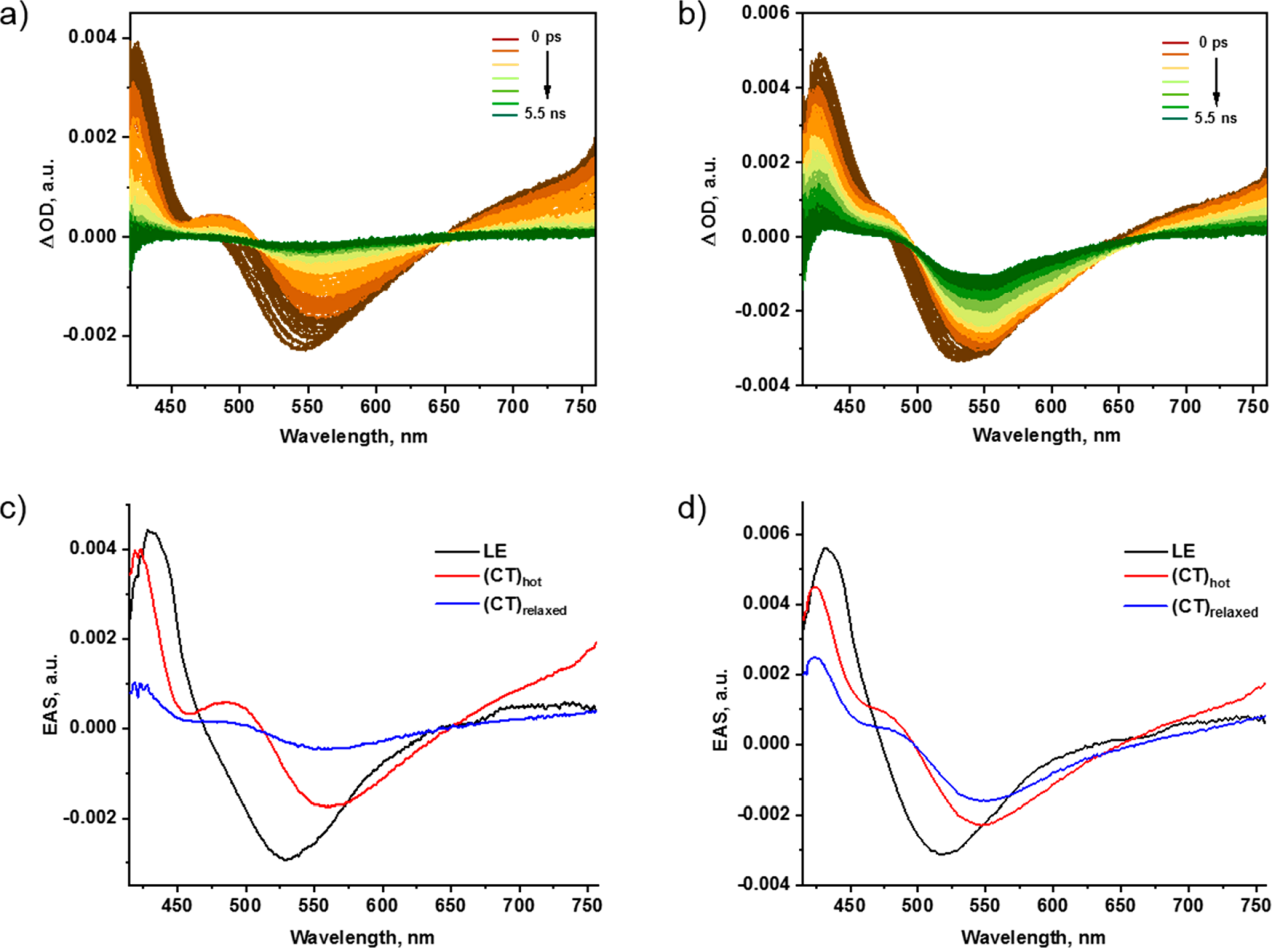
Femtosecond differential absorption spectra of (a) receptor **6** and (b) receptor **6** + CTP (100 equiv), at time delays between 0 and 5.5 ns after 387 nm photoexcitation at room temperature. Measurements were done in 50 mM MOPSO buffer (5% DMSO, pH 6.2). Evolution-associated spectra reconstructed from the sequential global analysis of femtosecond transient absorption (TA) spectra of (c) receptor **6** and (d) receptor **6** + CTP (100 equiv).

To further discern the origin of the fluorescence enhancement, we investigated the excited state dynamics of receptor **6** in the absence and presence of CTP as well as GTP by means of femtosecond TA spectroscopy. Upon photoexcitation at 387 nm, the TA spectrum of **6** shows differential changes, which include excited state absorptions (ESA) at 425, 490, and beyond 650 nm, next to stimulated emission (SE) at 540 nm (Figure 6a). Global sequential analysis of the TA data was performed with the GloTarAn program,^78^ using a kinetic model based on three species. The resulting evolution-associated spectra are shown in Figure 6c. The first excited species exhibits an ESA at 428 nm and a SE centered at around 530 nm, which leads us to attribute it to a locally excited (LE) state. Within a time span of 3 ps, the aforementioned LE state deactivates and transforms into a second species. It is characterized by a marked ESA at 490 nm, which corresponds to the naphthalimide radical anion fingerprint, in addition to SE that is centered around 560 nm.^79^ In line with our spectroscopic assignment, we assign this second species to a vibrationally hot charge transfer (CT) state, that is, (CT)_hot_. The CT nature involves the electron-accepting naphthalimide and electron-donating piperazine. Its lifetime is 40 ps. Relaxation of (CT)_hot_ leads to the formation of the third and final species. As it features the same fingerprints as (CT)_hot_ we rationalize that its nature is (CT)_relaxed_. (CT)_relaxed_ then repopulates via the fluorescence ground state in 577 ps.

Upon the addition of CTP to receptor **6** (Figure 6b,d), we noted that the initially formed LE state undergoes an even faster relaxation than in its absence. Now, it is within less than 2 ps that a second species is formed. However, for the second species, a 480 nm shoulder rather than a 490 nm ESA, as seen in the case of receptor **6** is noted. Hand-in-hand with the ESA shift is the fact that the SE is blue-shifted to 550 nm. We ascribe this species to (CT)_hot_, however, slightly higher energy than in the case of free **6**. Its higher energy results from binding CTP, which draws the electron density upon protonation of the electron-donating piperazine. It lives for 28 ps and relaxes to yield the third species, which features a remarkably intense SE. This is in sound agreement with the fluorescence enhancement, which was observed in the steady-state measurements upon CTP addition. We postulate that the third species is the fluorescent (CT)_relaxed_, which is subject to a radiative recovery of the ground state with a lifetime of 2 ns. Likewise, addition of GTP to receptor **6** (Figure S33) leads to similar excited state decay dynamics. For GTP, the fluorescence enhancement is weaker than that for CTP, which corroborates our findings that we gathered in the fluorescence measurements.

### Interaction with Oligoribonucleotides

**Figure 7:**
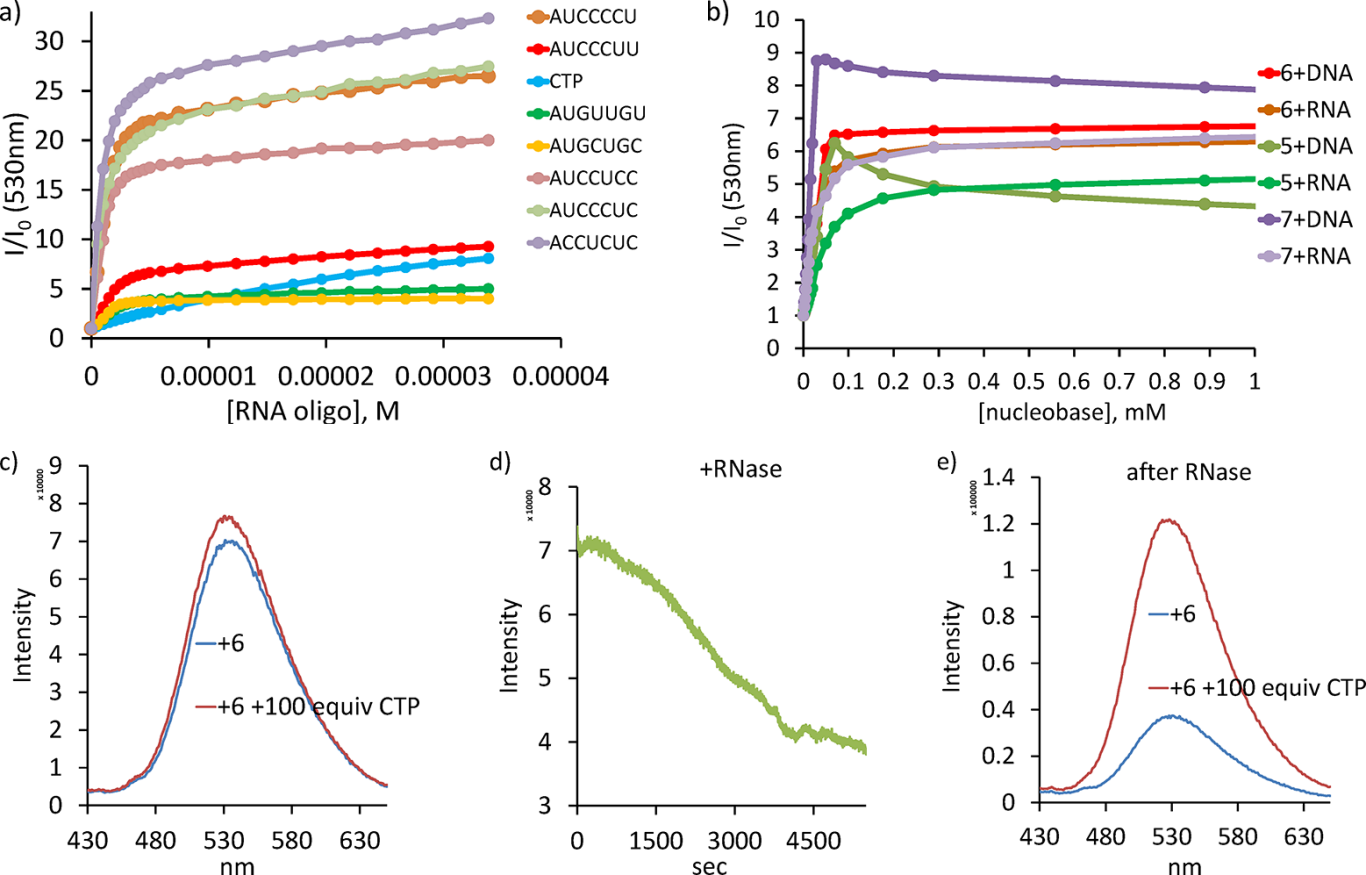
(a) Fluorescence changes observed for (a) **6** with heptanucleotides and (b) receptors **5**–**7** with DNA and RNA: 5 μM receptor, 0.1 mM oligo and 10 mg/mL nucleic acid, 50 mM MOPSO buffer (5% DMSO, pH 6.2). The concentration of nucleic acid is converted to an average concentration of nucleobases in solution. (c) Fluorescence of 5 μM **6** in cell lysate solution (4000 cells/μL, 50 mM MOPSO buffer, pH 6.2) before and after addition of 100 equiv of CTP. (d) Changes in intensity of **6** after the addition of RNase (70kU/mg, concentration in the cuvette 1 mg/mL) to the cell lysate (e) followed by addition of 100 equiv of CTP.

The ability of receptors to bind nucleotides and nucleobases prompts to their interactions with longer nucleotide sequences and nucleic acids. To this end, we carried out experiments with short oligoribonucleotides (RNAs) under the same conditions, which were used for the titration experiments. In the first set of experiments, we tested RNA heptanucleotides with different positions of cytosine in the sequences: internal and terminal positions, several in a raw, and without cytosine. The selected sequence AUCCC(*C*)U is present in the secondary structure of PV1.^65^ In other sequences, cytosine was included at internal and terminal positions: 5′-AUCCCCU-3′, 5′-AUCCCUU-3′, 5′-AUCCCUC-3′, 5′-AUCCUCC-3′, 5′-ACCUCUC-3′, 5′-AUGUUGU-3′, and 5′-AUGCUGC-3′. According to the binding studies, the fluorescence enhancement increases with the number of cytosines in the sequence indicating that the receptor binds preferably to cytosine. The “turn-on” response of receptor **6** for the AUCCCCU and AUCCCUC sequences was 25-fold, while ACCUCUC showed 30-fold enhancement (Figure 7). Interestingly, we found a 2:1 (receptor-oligo) binding stoichiometry with heptanucleotides with two or three cytosine, while sequences with four cytosines showed 3:1 binding stoichiometry. The interaction strength of **6** with AUGCUGC (log *K*_21_ = 12.80 ± 0.05) and with AUCCCUU (log *K*_21_ = 12.82 ± 0.03) were the same order of magnitude. In contrast, binding to AUGUUGU was almost two orders of magnitude weaker with a log *K*_21_ of 11.06 ± 0.03. According to the determined binding constants (Table S1), the ratio *K*_31_/*K*_21_ and the fluorescence enhancement is higher for those sequences in which cytosines are separated with uracil. For instance, the sequence ACCUCUC shows 30-fold enhancement and binding affinity log *K*_31_ = 17.47 ± 0.03 and log *K*_21_ = 8.20 ± 0.03, suggesting that the receptor is better bound to the separated cytosines as compared to those placed next to each other.

Finally, we studied the interactions of **5**–**7** with naturally occurring nucleic acids: calf thymus DNA (double-stranded) and yeast RNA (single-stranded) (Figure 7). The inflection points for RNA and DNA were observed at approximately 1:5 and 1:10 receptor–nucleobase ratios, respectively. The receptors bind to both of these nucleic acids. However, the inflection point in the fluorescence titrations depends on the hybridization state of the nucleic acid, that is, single- versus double-stranded. The fitting analysis shows that the apparent association constant at each binding site is 10^5^ M^–1^. The fluorescence response and affinity to nucleic acids were similar at pH 6.2 and pH 7.4. Overall, these three sets of experiments suggest that the receptors in the competitive environment (under cell-like conditions) would prefer association with nucleic acids rather than single nucleotides.

To provide additional evidence of this fact, we investigated the ability of **6** to detect CTP in cell lysate (human ovarian cancer cells A2780, diluted in MOPSO buffer pH 6.2). A solution of cells with a concentration of 1500 cells/μL and receptor **6** showed fivefold fluorescence increase after addition of 100 equiv of CTP (Figure S41). Then, we prepared more concentrated cell lysate (4000 cells/μL) and treated with **6**. Addition of 100 equiv of CTP to this solution resulted in almost no intensity change (1.1-fold increase), indicating a strong competition of cell content with CTP binding (Figure 7c). However, after addition of RNase and completion of hydrolysis, the addition of CTP induced fourfold fluorescence enhancement (Figure 7e). These experiments nicely show that **6** is bound to RNA even in the presence of large excess CTP; however, after RNA hydrolysis, **6** again is able to detect CTP. Notably, **6** functions as a fluorescence indicator of RNA hydrolysis: a steady decrease in the intensity over 2 h was observed (Figure 7d).

### Cellular Effects of Receptors

We investigated whether our receptors can bind C-containing biomolecules directly in live cells by using fluorescence imaging. Additionally, we explored whether this binding is cancer cell-specific and whether it affects the viability of cancer cells. First, we identified that all receptors with the exception of **2** affect the viability of human ovarian cancer cells A2780 with IC_50_ values from 0.8 ± 0.1 μM (for **5**) to 3.0 ± 0.4 μM (for **1**, Table 3). IC_50_ is a receptor concentration at which half of the cells remain viable as determined using the viable cell stain 3-[4,5-dimethylthiazol-2-yl]-2,5-diphenyltetrazolium bromide (MTT). Compounds **1**, **3**, and **5** bearing a xylylene linker showed the highest activity. It is highly likely that the more hydrophobic nature of these receptors as compared to those featuring a pyridine spacer enhances their uptake into cells.

**Table 3:** IC_50_ Values Determined for Receptors **1–7** by MTT Assay with A2780 Cells

compounds	48 h incubation IC_50_ (μM), A2780, *n* = 3
**1**	3.0 ± 0.4
**2**	>50
**3**	1.8 ± 0.3
**4**	3.1 ± 0.1
**5**	0.8 ± 0.1
**6**	2.3 ± 1.0
**7**	3.2 ± 0.9
FeHQ^a^	3.3 ± 0.3

a Ferric chloride/8-hydroxyquinoline complex, 1/2, eq/eq (positive control).

**Figure 8:**
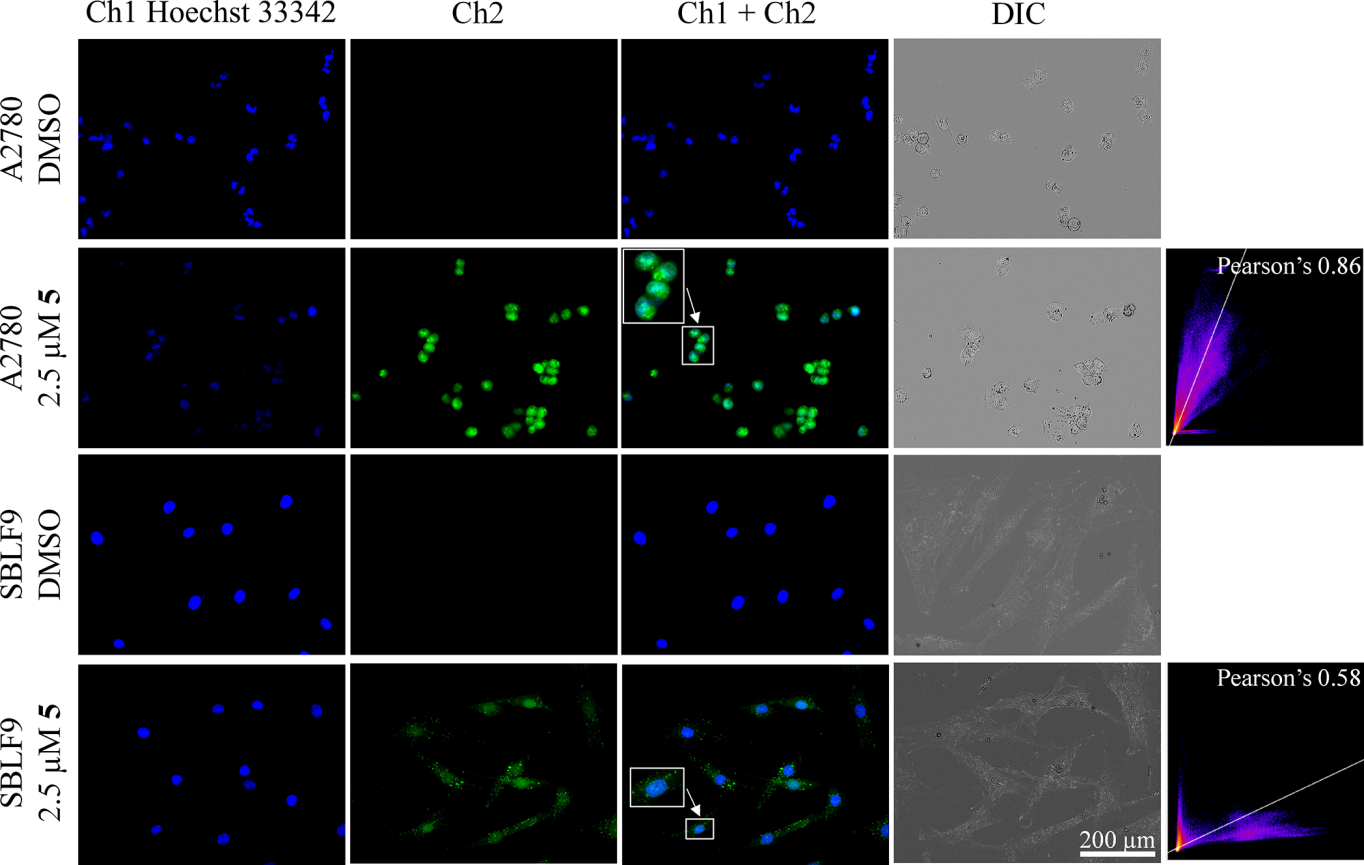
Localization of **5** in A2780 and SBLF9 cells. Cells were incubated with 2.5 μM **5** or just the carrier DMSO for 2 h before they were incubated with 1 μg/mL Hoechst 33342 (observed in Ch1) for 20 min. Fluorescence images were taken with a Zeiss Axio Observer fluorescence microscope with a 40×/1.30 Oil DIC objective. Channel 1 (blue): λ_ex_: 365 nm; λ_em_: 445/50 nm; Channel 2 (green): λ_ex_: 470/40 nm; λ_em_: 525/50 nm. The section in the frame is shown enlarged by a factor of two. In case of the SBLF9 cells, the signal was additionally increased to make the localization better visible.

**Figure 9:**
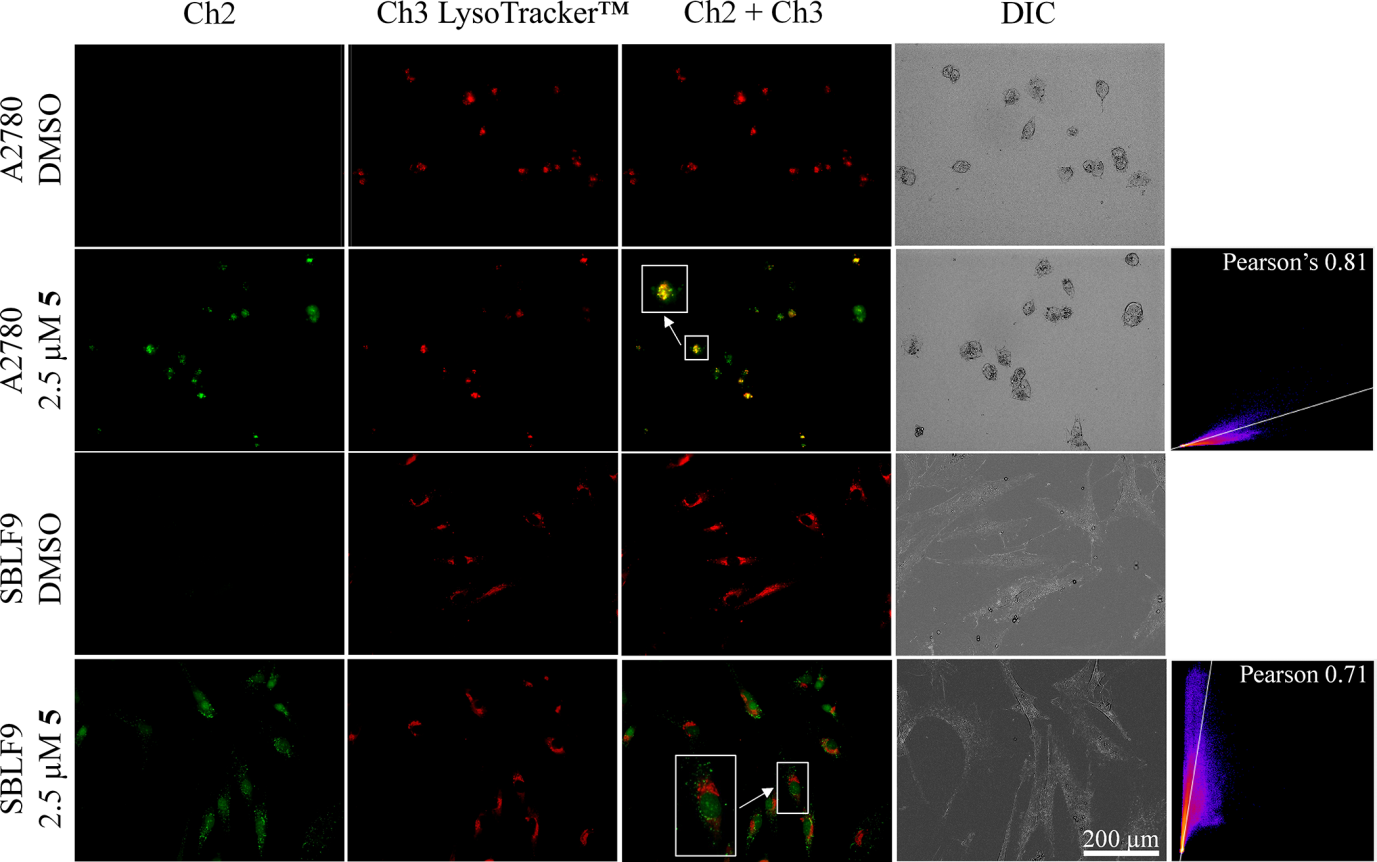
Localization of **5** in A2780 and SBLF9 cells. Cells were incubated with 2.5 μM **5** or just the carrier DMSO for 2 h before they were incubated for 20 min at 37 °C with LysoTracker Deep Red (500 nM in 2 mL of Hank’s Balanced Salt Solution, 0.1% DMSO). Channel 2 (green): λ_ex_: 470/40 nm; λ_em_: 525/50; Channel 3 (red): λ_ex_: 640/30 nm; λ_em_: 690/50 nm. The section in the frame is shown enlarged by a factor of two.

Next, we determined the cell specificity of the most active receptor, namely, **5**. This receptor appeared to be the only in the family that shows 20 nm red shift in absorption (Figure S43) upon interaction with RNA. Thus, it was possible to excite the receptor at 470 nm without excitation of Hoechst 33342. We observed that its uptake by representative cancer cells (A2780) is substantially more efficient than that by normal cells (SBLF9 fibroblasts) (Figures 8 and S40). Compound **5** is uptaken in the cancer cells and distributed throughout the whole cell including nucleus. It is also observed that the fluorescence of Hoechst 33342 is quenched, which can originate from either the competition process or by the ability of **5** to absorb the emitted light from the dye. For the normal cells, a different pattern of the receptor distribution is clearly visible. The overall intensity of the receptor is much less than in cancer cells. In normal cells, the receptor signal was only partially overlapping with the one of Hoechst 33342 (Pearson’s coefficient SBLF9: 0.58 versus A2780: 0.86) showing that the uptake of the compound into the nucleus is less efficient as compared to cancer cells. The receptor rather seems to be accumulated in distinct punctae which correspond to lysosomes and likely RNA distributed in cells. Colocalization studies with LysoTracker Deep Red indeed showed an overlap of the receptor and dye signals in both cancer and normal cells. (Figure 9).

**Figure 10:**
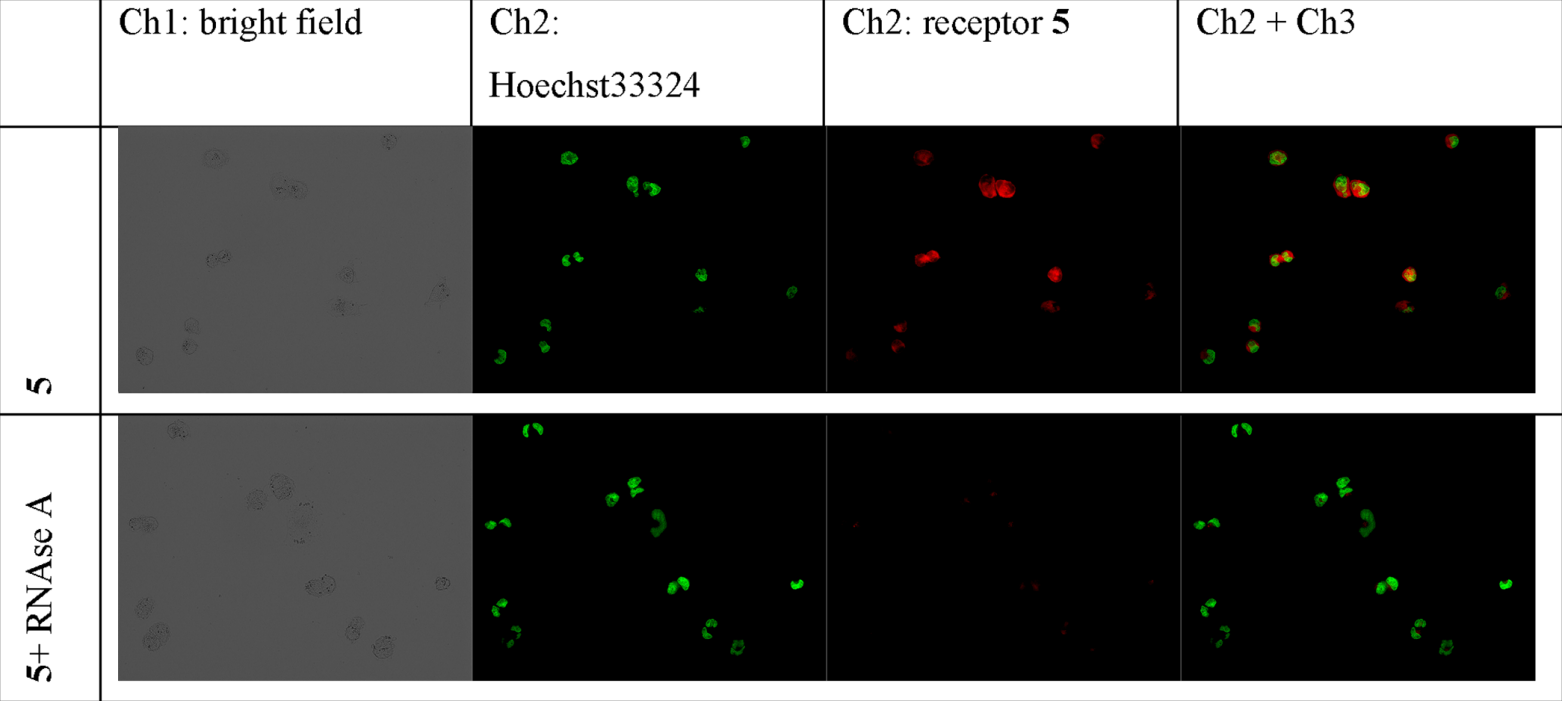
RNase A digestion assays. RNase A solution (0.1 mg/mL RNase A in DPBS) was added and incubated for 30 min at 37 °C. As a negative probe (probe B), cells were treated with 1 mL of DPBS solution for 30 min at 37 °C. Ch1: ex. 365 nm, em. 395–495 nm. Ch2 ex. 430–510 nm, em. 475–575 nm.

In the cytoplasm, **5** might bind either to nucleotides and to RNA. To find out, which scenario prevails, we conducted two additional experiments. Both experiments were done with A2780 cells, in which the membrane was disrupted by treating it with formaldehyde (4% in DPBS). All cells were washed thoroughly (2 × 2 mL DPBS) to remove all low-molecular-weight biomolecules, including nucleotide derivatives. One part of the cells was treated with RNase A to eliminate intracellular RNA (probe A) and another one was used as is (probe B). Afterward, the cells in probes A and B were stained with **5** and counterstained with Hoechst 33342. We observed that probe B is stained well with **5** and a similar pattern is realized as in live cells loaded with **5** (Figure 10). In contrast, RNase A-treated probe A is not stained at all by **5**. This observation confirms that **5** binds to intracellular RNAs rather than to low-molecular-weight biomolecules such as nucleotides and nucleotide mono-, di-, or triphosphates. The binding to RNA has the potential to inhibit RNA processing and RNA-mediated processes, such as protein synthesis, thereby leading to cancer cell death.

## Conclusions

We have developed a new family of receptors with good water solubility that bind nucleoside phosphates in water with affinities >10^2^–10^4^ M^–1^, depending on the structure of *spacers A* and *B*. We have shown that minor modifications of the spacers have a dramatic effect on the overall binding and sensing properties. To this end, we have tuned the structure to obtain receptor **6**, which binds and detects CTP in an aqueous buffered solution (50 mM MOPSO buffer, pH 6.2) with high selectivity.

Receptors **6** and **7** have demonstrated record 40- and 60-fold fluorescence enhancements in the presence of CTP, respectively. These values are at least one order of magnitude higher than the response for other nucleoside triphosphates. Experimental and theoretical investigations suggest that cytosine is coordinated to *spacer B* between the naphthalimide rings through hydrogen bonds, while the phosphate residue is bound to the piperazine moieties. As a matter of fact, the latter interactions are responsible for the strong fluorescence enhancement. This is caused by the protonation of the piperazine-naphthalimide subunits, which blocks PET.

The rational design shown in this work may help to understand the fundamental principles of constructing receptors that demonstrate high selectivity for nucleotides under biological conditions. To the best of our knowledge, receptor **6** is the first receptor that shows strong and selective fluorescence enhancement in the presence of CTP and cytosine-rich sequences. This is an essential step toward the recognition of specific RNA sequences to treat related diseases. Receptor **6** can also be considered the first functional model of poly(rC)-binding protein, which is known to show a high affinity for poly(C) in a sequence-specific manner. We have established micromolar cytotoxicity for receptors, which originates from their ability to specifically bind to RNA in cells. This property holds great promise for the development of new supramolecular hosts with the ability of sequence-specific RNA binding to treat a wide variety of diseases.

## Methods

### General Synthetic Methodology

All the solvents were dried according to standard procedures. Reactions were performed in an oven-dried round bottom flask. Crude products were purified by column chromatography on silica gel purchased from Macherey-Nagel. TLC plates were visualized by exposure to ultraviolet light and/or by exposure to acidic ethanolic solution of ninhydrin followed by heating (<1 min) on a heat gun (∼250 °C). Organic solutions were concentrated on a rotary evaporator at 35–50 °C. NMR spectra were measured on a Brucker Avance 400 and 600 MHz and in various deuterated solvents. The chemical shifts are reported in δ [ppm] relative to external standards (solvent residual peak). Mass spectra were recorded on Bruker micrOTOF II focus (BRUKER Daltonics GmbH) and SHIMADZU Axima Confidence maXis 4G instruments (nitrogen UV laser, 50 Hz, 337 nm). Absorption spectra were measured in 1 cm quartz cuvettes with a Varian Cary 50 BIO UV/VIS/NIR Spectrometer. Emission spectra were recorded with aqueous buffered solution in 1 cm quartz cuvettes (Hellma) on a FluoroMax 4 Plus (Horiba, CA, USA) with a temperature control. pH-measurements were carried out on a Mettler Toledo G20 Titrator equipped with a DG115-SC pH-electrode. The electrode was calibrated with standard calibrating solutions from Mettler Toledo. The starting compounds were purchased from TCI (Germany), Sigma-Aldrich (Germany), and Acros Chemicals (Belgium).

Full synthetic details are outlined in the SI.

### Fluorescence and UV–vis Titrations

Stock solutions of receptors with concentrations of 10^–5^ M in a 50 mM MOPSO buffer (5% DMSO, pH 6.2) were prepared for spectroscopic titrations. The titrant (0.01 M) was sequentially added to a 2 mL sample of the host stock solution in the spectrometric cell and the changes in the spectral features were monitored. The total number of data points was 20–40, depending on the stoichiometry of complexation; for a presumed 1:1 complex, 20 points were usually measured. The following setup parameters were used for fluorescence titration experiments: ex. 400 nm, slit 2/2, em: 410–650 nm; for UV–vis measurements 330–495 nm.

### Femtosecond TA Spectroscopy

Measurements were performed with a Helios (0–5500 ps) pump/probe setup from Ultrafast Systems. The samples were excited at a 387 nm wavelength and the laser source was a CPA-2110 titanium:sapphire amplifier (1 kHz repetition rate, 150 fs pulse width, 1000 nJ laser energy) from Clark-MXR Inc. White light was generated using a 2 kHz continuous white light laser. Global analyses of the TA data were performed with GloTarAn software.

### Studies with Live Cells

The fluorescence of live cells was quantified using CytoFLEX S (Beckman Coulter, USA). The microscopy images were taken with a Zeiss Axio Vert.A1 microscope in 35 mm μ-Dish Imaging dishes (ibidi GmbH, Germany). Human ovarian cancer cell line A2780 was purchased from Sigma-Aldrich and cultivated in Roswell Park Memorial Institute 1640 (RPMI), supplemented with 10% (v/v) fetal bovine serum (FBS), 1% (v/v) penicillin/streptomycin (Pen/Strep), and 1% (v/v) l-glutamine (l-Glu). SBLF9 primary human fibroblasts were isolated via skin biopsy after local anesthesia from a healthy 20-year-old Caucasian male and subsequently cultivated in F-12 medium supplemented with 15% (v/v) FBS, 2% nonessential amino acid mix, and 1% Pen/Strep. Cells were cultivated to around 80% confluence.

## Supporting Information Available

Experimental details and procedures, optimization details, fluorescence, NMR and UV–vis data for all new compounds (PDF)
