# Supplementary material for: Supramolecular Recognition of Cytidine Phosphate in Nucleotides and RNA Sequences
Source: JACS Au. 2023 Feb 13;3(3):964–77. doi: 10.1021/jacsau.2c00658 (PMC10052242; doi:10.1021/jacsau.2c00658)
Supplement: Supplementary file 1 — au2c00658_si_001.pdf [file au2c00658_si_001.pdf]

Supporting Information

Supramolecular Recognition of Cytidine Phosphate in Nucleotides and RNA Sequences

Boris S. Morozov,[a] Aleksandr S. Oshchepkov,[b] Insa Klemt,[a] Aleksandr M. Agafontsev,[a] Swathi Krishna,[c] Frank Hampel,[a] Hong-Gui Xu,[a] Andriy Mokhir,[a] Dirk Guldi[c]* and Evgeny Kataev[a]*

[a]Department of Chemistry and Pharmacy, Friedrich-Alexander Universität Erlangen-Nürnberg, Nikolaus-Fiebiger-Str. 10, 91058 Erlangen (Germany)

*E-mail: evgeny.kataev@fau.de

[b]Max-Planck-Institut für die Physik des Lichts, Staudtstraße 2, 91058 Erlangen (Germany)

[c] Department of Chemistry and Pharmacy, Interdisciplinary Center for Molecular Materials (ICMM), Friedrich-Alexander-Universität Erlangen-Nürnberg, Egerlandstr. 3, 91058 Erlangen (Germany)

*E-mail: dirk.guldi@fau.de

Content

Synthesis of compounds 2

Fluorescence-pH relationship 27

Potentiometric titrations 28

Fluorescence and UV-Vis titrations 29

  Competition experiment 41

  The limit of detection (LOD) and limit of quantitation (LOQ) 42

  Femtosecond transient absorption spectroscopy 43

Electrospray ionization (ESI) studies 43

^{1}H NMR studies 45

Studies with live cells 47

Synthesis of compounds

Synthesis of starting materials

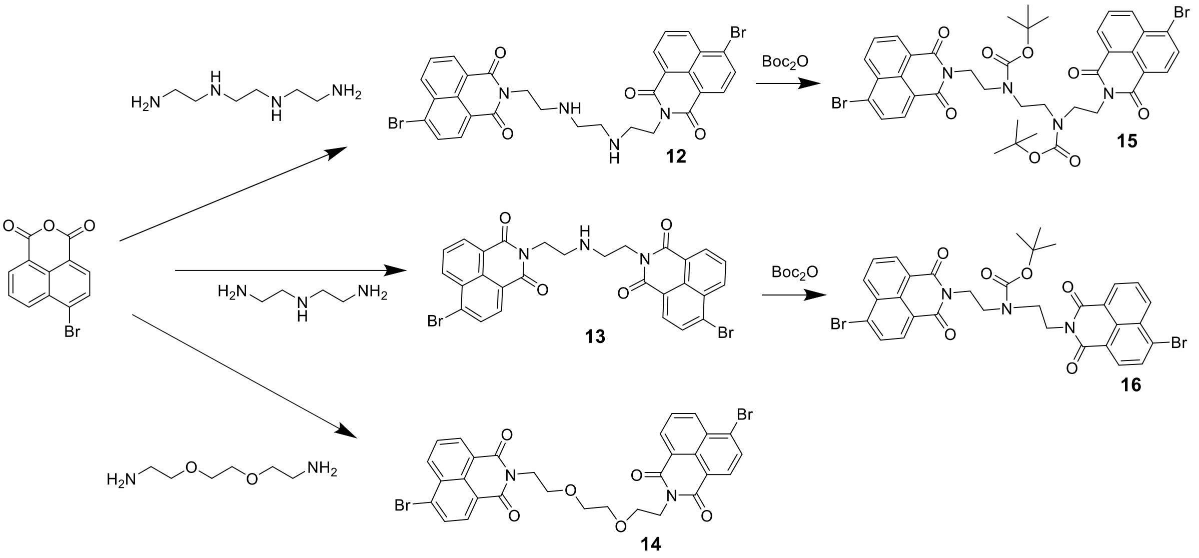

General procedure for the synthesis of compounds 13 and 14.

The solution of the corresponding amine (7.5 mmol) in 5 ml of ethanol was added to the suspension of 1,8-naphthalic anhydride (4.16 g, 15 mmol) in 120 ml of ethanol. The obtained mixture was refluxed for 10 h and poured in 200 ml of water. The precipitate was filtered off and dried under vacuum.

Compound 12 and 15.
To a stirred suspension of 1,8-Naphthalic anhydride (6.2g, 22.38mmol) in ethanol (200ml) was added triethylenetetramine (1.64g, 11.19mmol, counting on a pure amine from a 60% solution) at once. Then the mixture was refluxed for 5 hours. The reaction mixture was then poured into 300ml of water and the precipitate was filtered off and dried. As the starting material (Triethylenetetramine) exists in a mixture of different amines, the obtained reaction mixture was put in the reaction with di-tert-butyl dicarbonate without purification. The reaction mixture was dissolved in THF (400ml), cooled down to 0°C with the ice bath, and to a resulting suspension di-tert-butyl dicarbonate (9.81g, 45mmol) and potassium carbonate (6.9g, 50mmol) were added. After this the ice bath was removed and the reaction mixture was stirred overnight. Then the solvent was evaporated, the crude mixture was dissolved in chloroform and washed with water 3x100ml, dried over Na_2SO_4. Column chromatography: $CHCl_3$/EtOAc=4/1; Yield 40%

Compound 13. Yield 85%. ^{1}H NMR (500 MHz, $CDCl_3$) δ 8.44 (dd, *J* = 8.5, 1.0 Hz, 1H), 8.28 (dd, *J* = 7.3, 1.1 Hz, 1H), 8.03 (d, *J* = 7.8 Hz, 1H), 7.84 (d, *J* = 7.8 Hz, 1H), 7.65 (dd, *J* = 8.4, 7.3 Hz, 1H), 4.22 (t, *J* = 6.0 Hz, 2H), 3.02 (t, *J* = 6.0 Hz, 2H). ^{13}C NMR (101 MHz, $CDCl_3$) δ 163.4, 131.5, 131.1, 130.2, 129.7, 128.6, 127.6, 122.7, 121.8, 46.9, 39.4. HRMS (ESI): m/z Calc. for $[M+H]^+$: $C_{28}H_{20}Br_2N_3O_4$ 619.9815, found 619.9820. M.p. 240.6 – 245.0°C with decomposition.
Compound 14. Yield 87%. ^{1}H NMR (600 MHz, $CDCl_3$) δ 8.54 (d, *J* = 7.2 Hz, 1H), 8.45 (d, *J* = 8.4 Hz, 1H), 8.29 (d, *J* = 7.8 Hz, 1H), 7.93 (d, *J* = 7.8 Hz, 1H), 7.76 – 7.69 (m, 1H), 4.28 (t, *J* = 6.1 Hz, 2H), 3.69 (t, *J* = 6.1 Hz, 2H), 3.59 (s, 2H). ^{13}C NMR (101 MHz, $CDCl_3$) δ 163.6, 163.5, 133.2, 132.0, 131.2, 131.1, 130.5, 130.2, 128.9, 128.0, 123.0, 122.2, 70.2, 67.9, 39.2. HRMS (ESI): m/z Calc. for $[M+H]^+$: $C_{30}H_{23}Br_2N_3O_6$ 664.9923, found 664.9922. M.p. 215.8 – 217.5°C.
Compound 15. Yield 75%. ^{1}H NMR (400 MHz, DMSO-*d*$_6$) δ 8.55 (m, 4H), 8.31 (dd, *J* = 15.1, 7.9 Hz, 2H), 8.21 (dd, *J* = 11.5, 7.8 Hz, 2H), 7.99 (m, 2H), 4.19 (m, 4H), 3.48 (m, 4H), 3.29 (m, 4H), 1.03 – 0.86 (m, 18H). ^{13}C NMR (101 MHz, DMSO-*d*$_6$) δ 163.5, 132.3, 132.0, 131.8, 131.6, 131.4, 130.3, 129.4, 129.2,

128.9, 78.8, 27.9, 27.9, 21.2, 14.6. HRMS (ESI): m/z Calc. for $[M+H]^+$: $C_{40}H_{41}Br_2N_4O_8$ 863.1291, found 863.1296. M.p. 128.3 – 131.5°C.

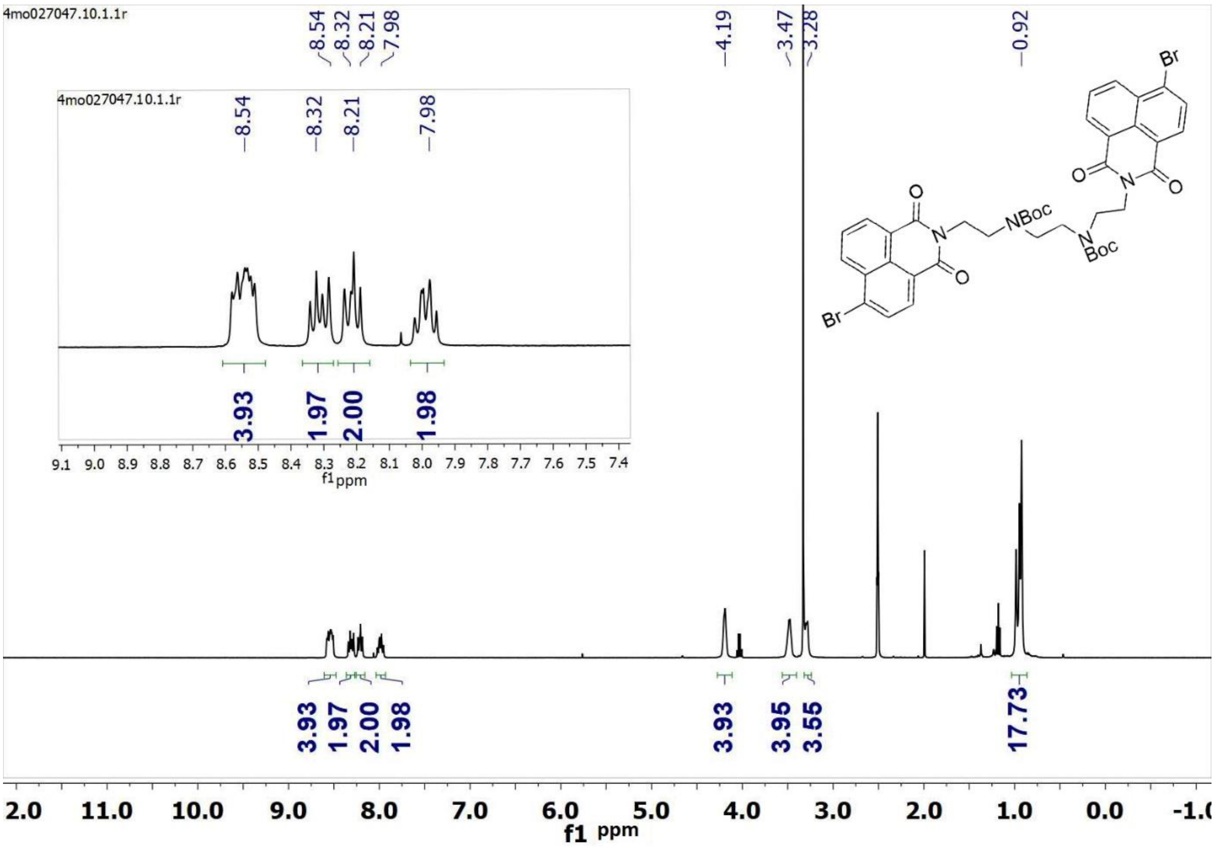

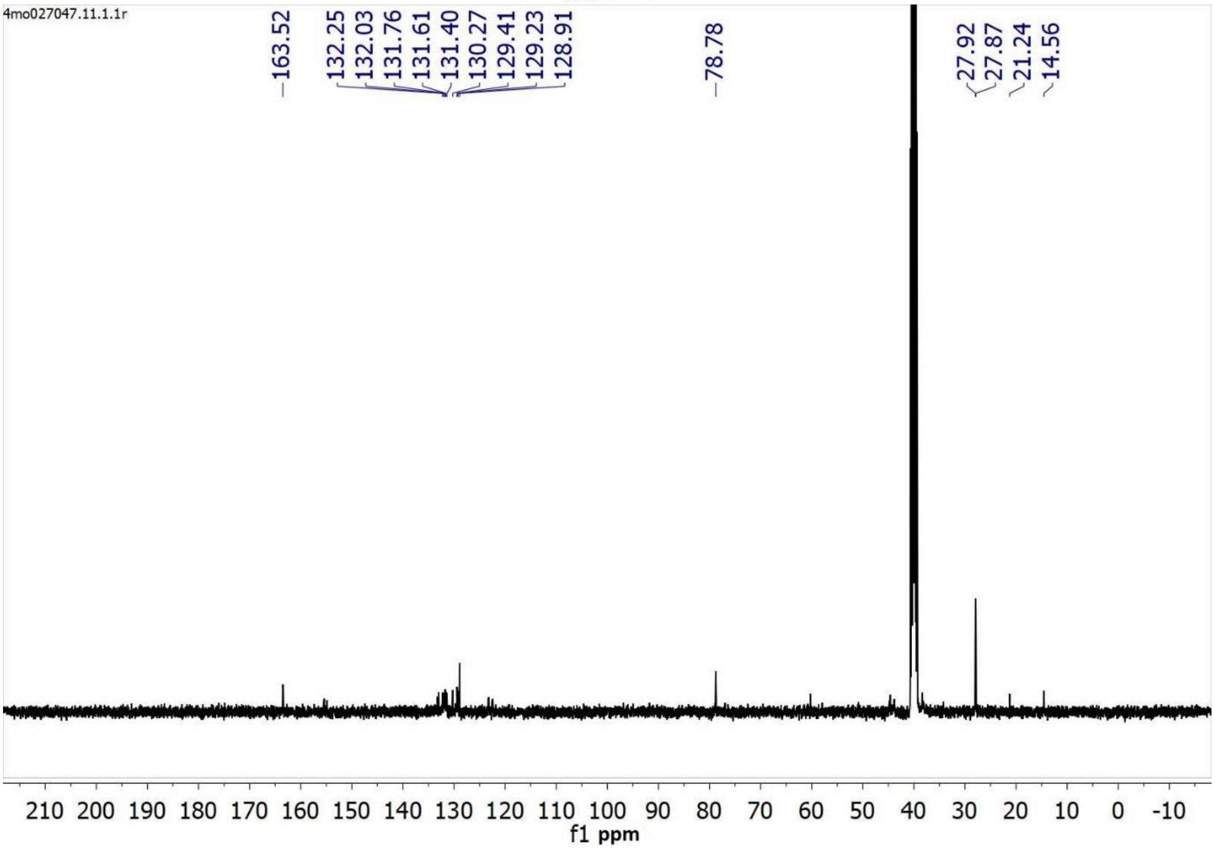

Figure S1. ^{1}H and ^{13}C NMR for **15**.

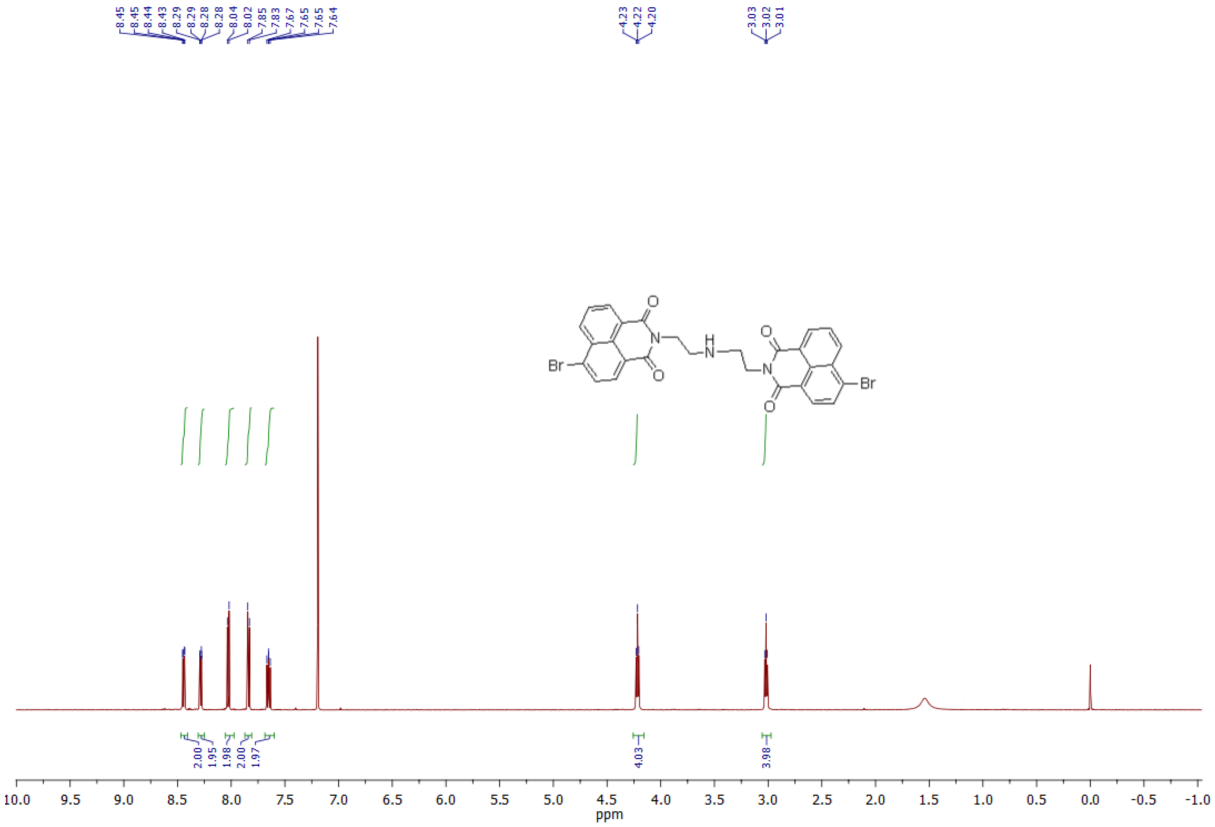

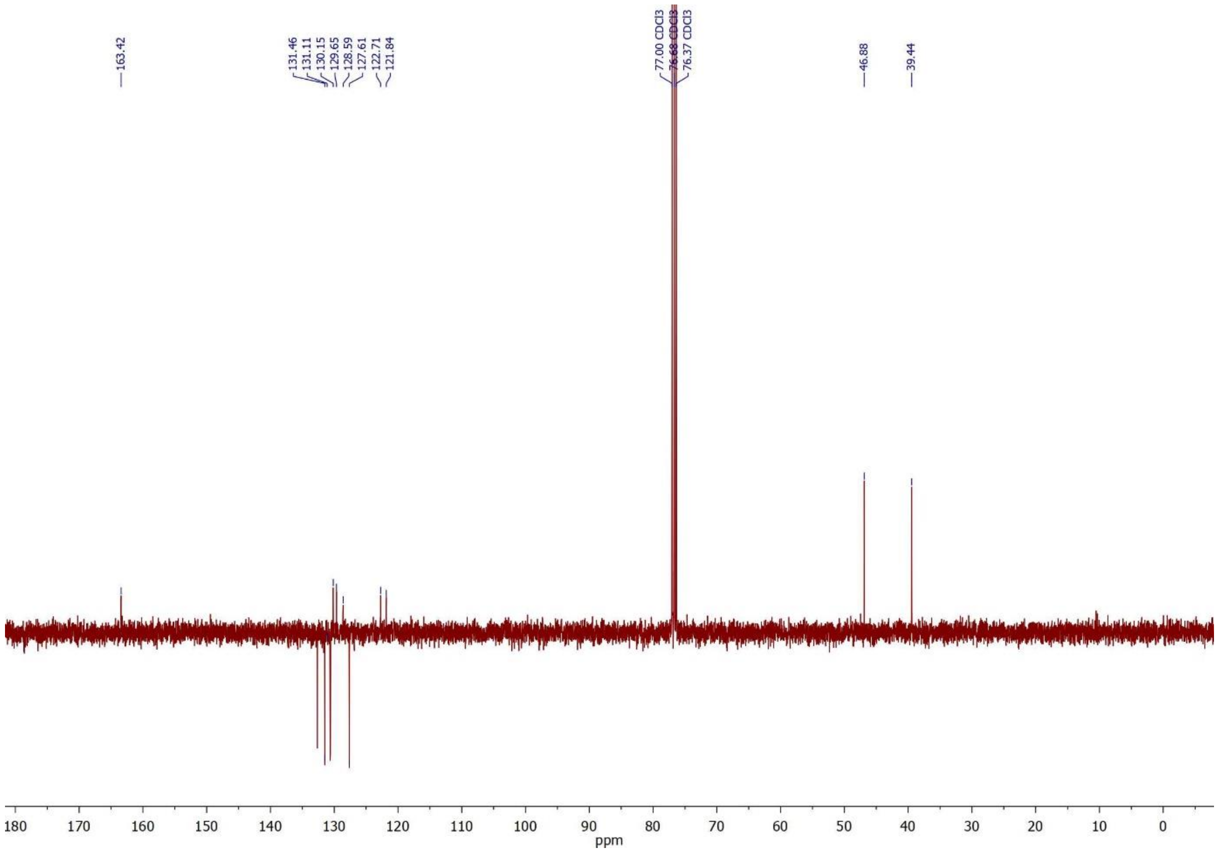

Figure S2. ^{1}H and ^{13}C APT NMR for **13**.

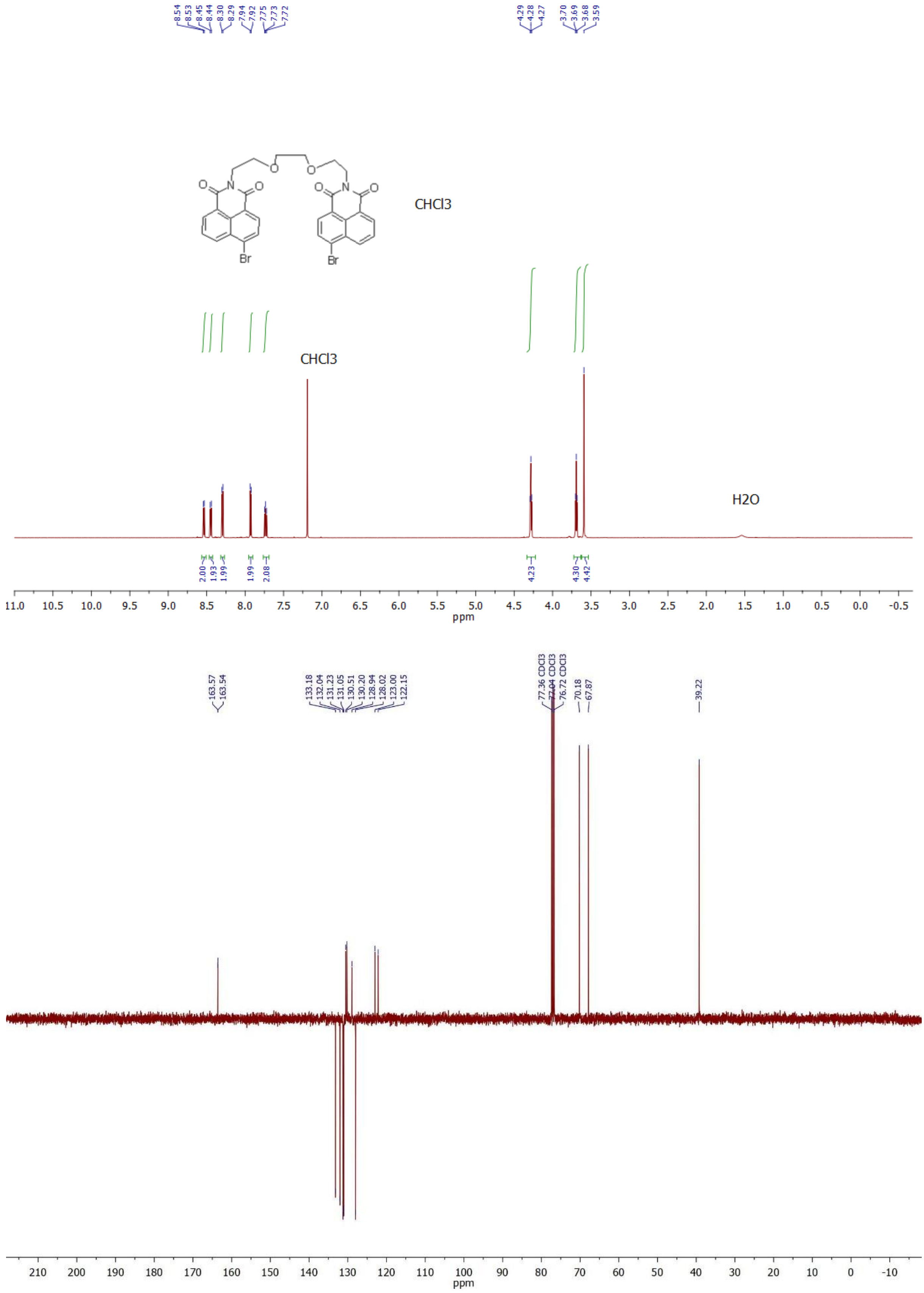

Figure S3. ^{1}H and ^{13}C APT NMR for **14**.

Synthesis of compound 16.

Starting material (5.76 mmol) was dissolved in chloroform (300 ml) – THF (100 ml) mixture and cooled with ice bath. Boc_2O in approx. 1.3 equiv pro amine group was slowly added to the stirring solution. Then the cooling was removed and the mixture was stirred for 2-3 h and followed by TLC until the complete disappearance of the starting material. The solution was evaporated and the product was purified by column chromatography by using chloroform-ethylacetate mixture 4:1 (v/v).

Compound 16. Yield 64%. ^{1}H NMR (600 MHz, $CDCl_3$) δ 8.62 (d, J = 7.2 Hz, 2H), 8.54 (dd, J = 21.8, 8.4 Hz, 2H), 8.38 (d, J = 7.9 Hz, 2H), 8.01 (dd, J = 14.0, 7.9 Hz, 2H), 7.82 (dd, J = 14.6, 7.5 Hz, 2H), 4.39 (d, J = 24.3 Hz, 4H), 3.68 (d, J = 38.6 Hz, 4H), 0.82 (s, 9H). ^{13}C NMR (101 MHz, CDCl3) δ 163.8, 163.5, 155.7, 133.4, 133.1, 132.2, 131.9, 131.3, 131.1, 130.9, 130.6, 130.3, 129.9, 129.1, 129.0, 128.1, 127.9, 123.2, 123.0, 122.4, 122.1, 79.4, 79.3, 44.6, 44.2, 38.1. HRMS (ESI): m/z Calc. for $[M+H]^+$: $C_{33}H_{28}Br_2N_3O_6$ 720.0345, found 720.0340. M.p. 139.3 – 142.5°C.

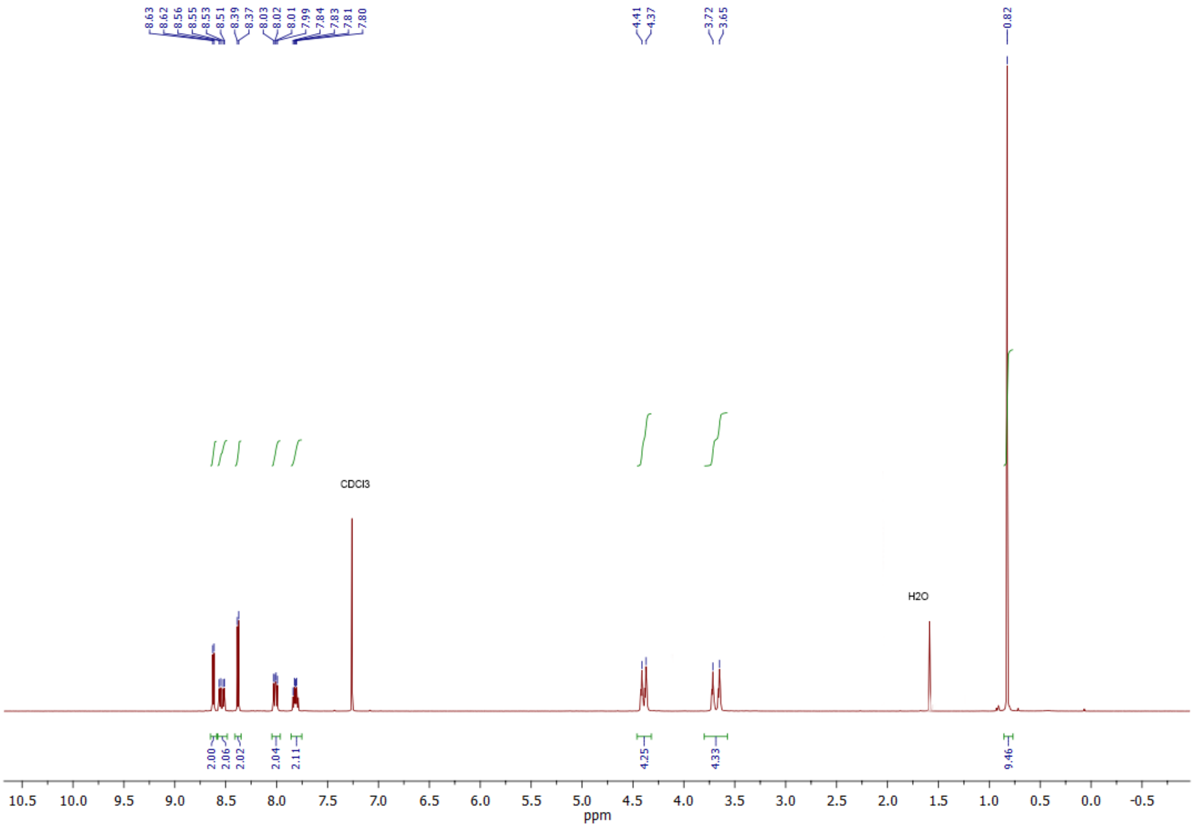

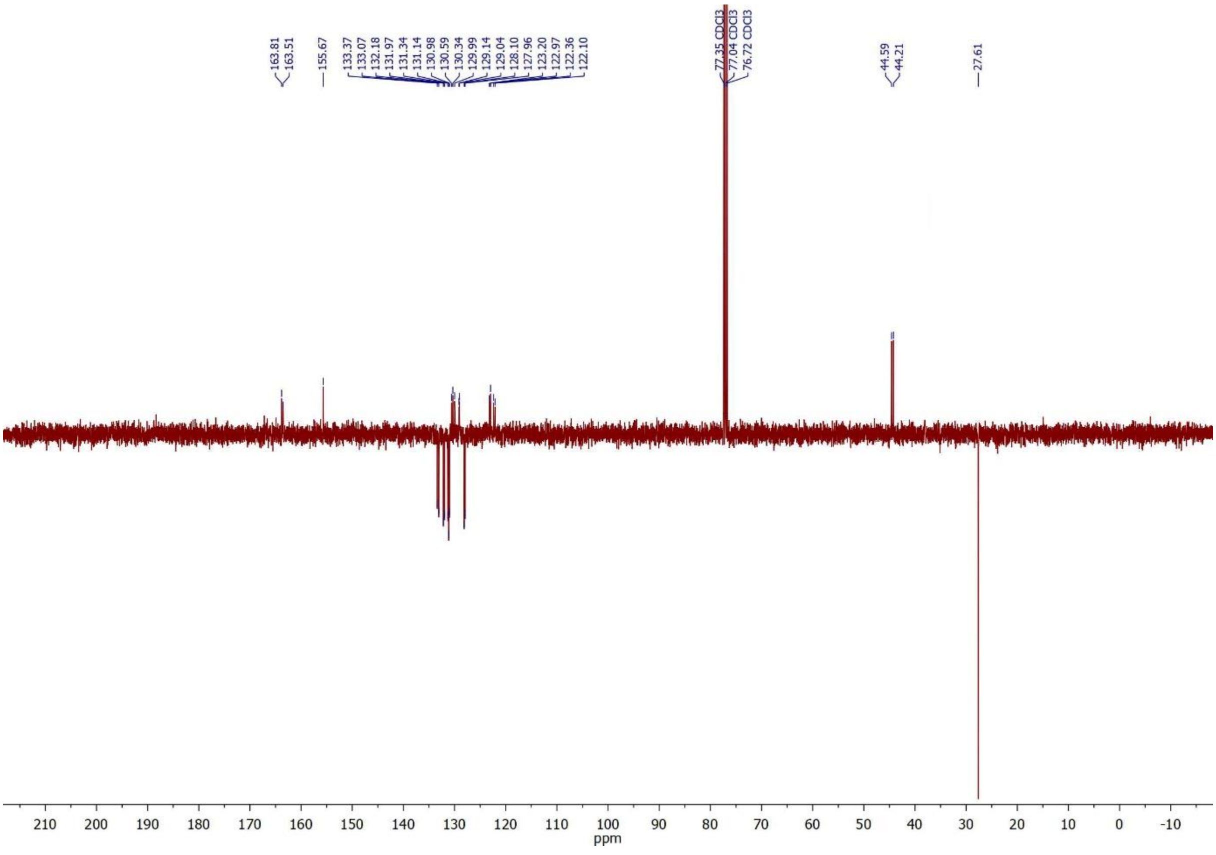

Figure S4. ^{1}H and ^{13}C NMR for **16**.

General procedure for the synthesis of compounds 17-19.

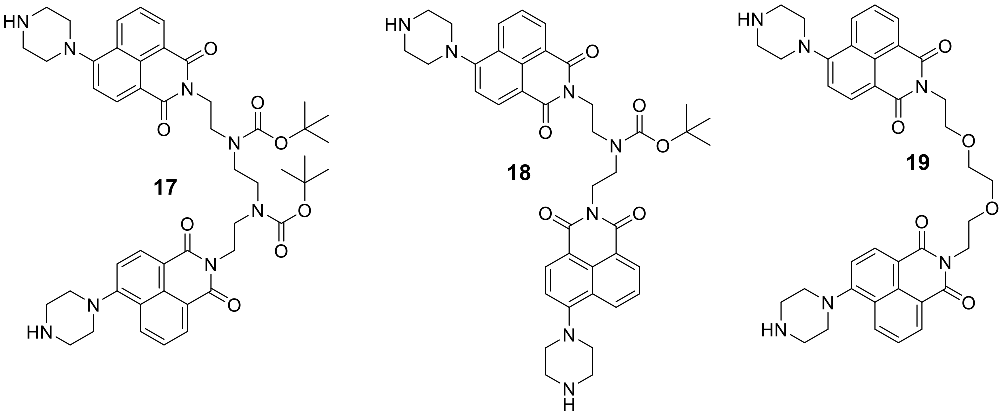

In a 100 ml flask piperazine (821 mg, 9.53 mmol), naphthyl-bromide derivative (0.79 mmol) and 30 ml of 2-methoxyethanol were placed together. The reaction was stirred at 90°C overnight until the starting material disappeared (the reaction was controlled by TLC). The solvent was removed under reduced pressure, then dried in vacuum and the product was purified by column chromatography by using chloroform-ethanol mixture 1:1 to wash off the minor byproducts. The product stayed on the column and then was washed with chloroform-ethanol-aqueous ammonia solution (100:100:4 vol.). After the column chromatography the product was dried in vacuum to complete dryness.

Compound 17. Yield 44%. ^{1}H NMR (400 MHz, DMSO-d_6) δ 8.48-8.30 (m, 6H), 7.79 (p, *J* = 7.5 Hz, 2H), 7.32 (dd, *J* = 8.2, 5.8 Hz, 2H), 4.18 (s, 1H), 3.45 (s, 5H), 3.28 (s, 5H), 3.14 (d, *J* = 31.8 Hz, 16H), 0.96 (dd, *J* = 30.9, 8.2 Hz, 18H). ^{13}C NMR (101 MHz, DMSO-d_6) δ 164.2, 163.6, 132.8, 132.6, 131.2, 129.8, 126.5,

125.8, 115.5, 78.7, 53.5, 45.5, 27.9, 27.8. HRMS (ESI): m/z Calc. for $[M+H]^+$: $C_{48}H_{59}N_8O_8$ 875.4450, found 875.4446. M.p. 146.5 – 147.0°C.

Compound 18. Yield 75%. ^{1}H NMR (600 MHz, $CDCl_3$) δ 8.52 (d, *J* = 7.2 Hz, 1H), 8.46 (d, *J* = 8.0 Hz, 1H), 8.37 (dd, *J* = 14.2, 8.4 Hz, 1H), 7.63 (dd, *J* = 16.5, 8.5 Hz, 1H), 7.16 (dd, *J* = 7.8, 6.1 Hz, 1H), 4.36 (d, *J* = 26.5 Hz, 2H), 3.65 (d, *J* = 37.2 Hz, 2H), 3.18 (d, *J* = 17.1 Hz, 7H). ^{13}C NMR (101 MHz, $CDCl_3$) δ 164.5, 164.3, 164.0, 163.8, 156.2, 156.0, 155.6, 129.9, 126.1, 125.6, 123.3, 123.1, 116.9, 116.6, 79.2, 54.1, 54.1, 52.8, 47.0, 46.0, 44.7, 44.3, 37.8, 37.7, 27.6. HRMS (ESI): m/z Calc. for $[M+H]^+$: $C_{41}H_{46}N_7O_6$ 732.3504, found 732.3504. M.p. 175.4 – 178.1°C.

Compound 19. ^{1}H NMR (600 MHz, $CDCl_3$) δ 8.53 (dd, *J* = 7.3, 1.5 Hz, 1H), 8.48 – 8.45 (m, 1H), 8.38 (dd, *J* = 8.4, 1.4 Hz, 1H), 7.67 – 7.61 (m, 1H), 7.19 – 7.13 (m, 1H), 4.38 – 4.31 (m, 2H), 3.78 – 3.72 (m, 2H), 3.66 (d, *J* = 6.5 Hz, 2H), 3.26 – 3.12 (m, 8H). ^{13}C NMR (101 MHz, DMSO) δ 162.6, 162.1, 154.8, 130.9, 129.4, 129.0, 128.3, 124.5, 124.2, 121.4, 114.7, 113.5, 68.5, 66.2, 52.3, 45.4, 44.3, 37.3. HRMS (ESI): m/z Calc. for $[M+H]^+$: $C_{41}H_{46}N_7O_6$ 677.3082, found 677.3085. M.p. 222.3 – 225.5°C.

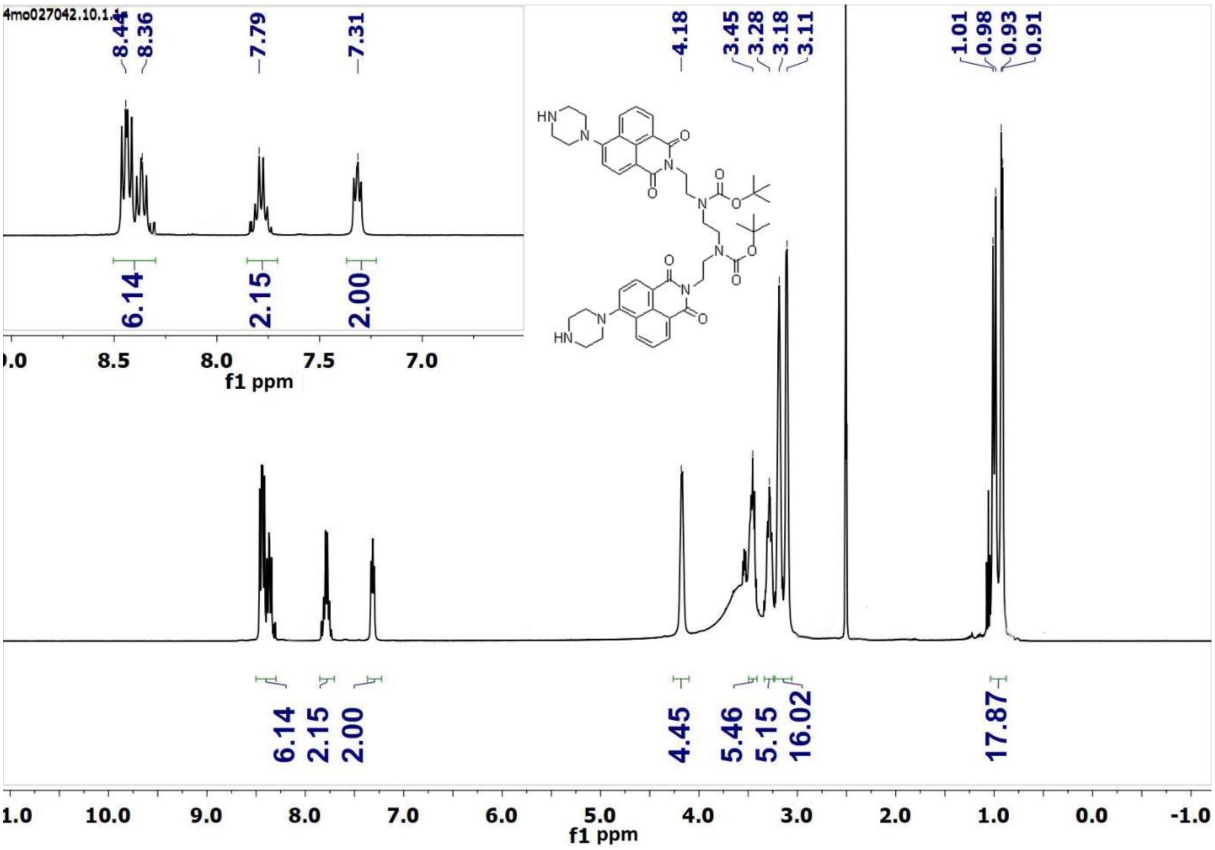

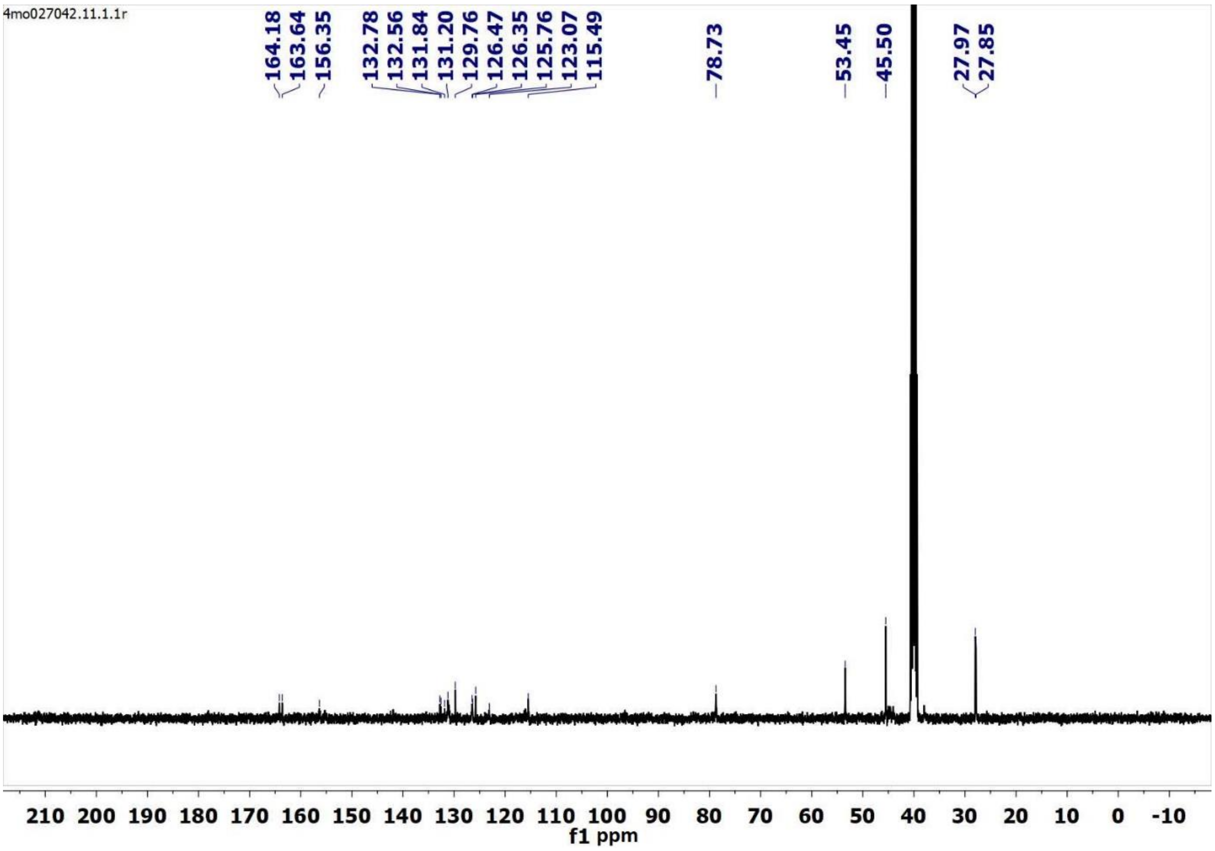

Figure S5. ^{1}H NMR ^{13}C of **17**

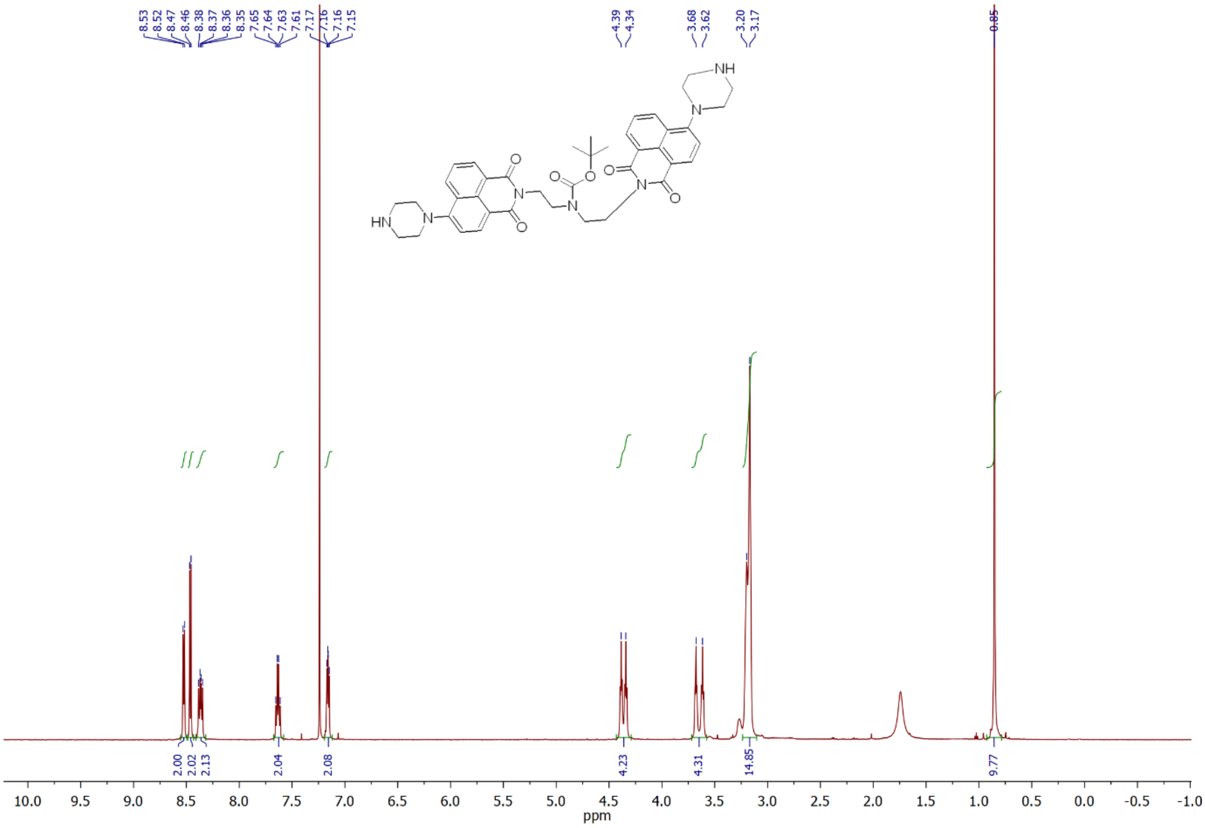

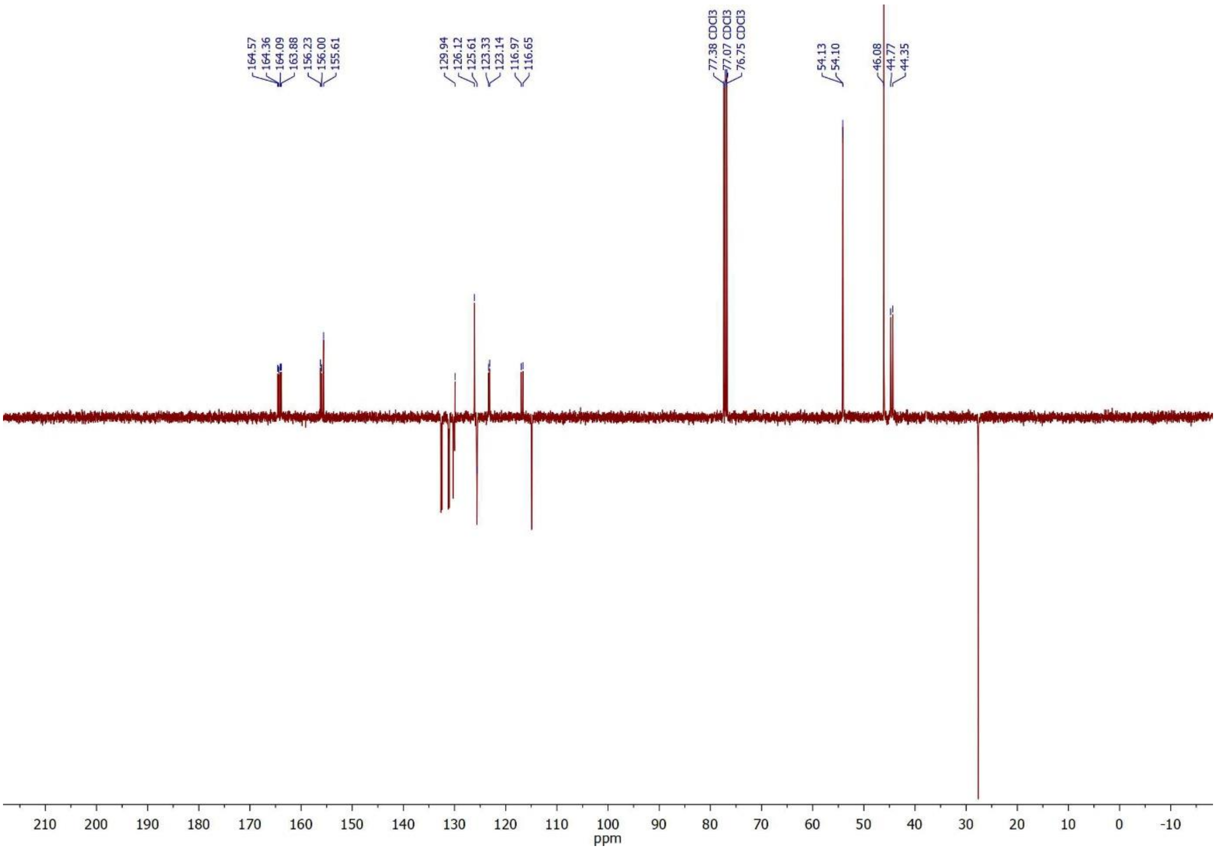

Figure S6. ^{1}H NMR ^{13}C APT of **18.**

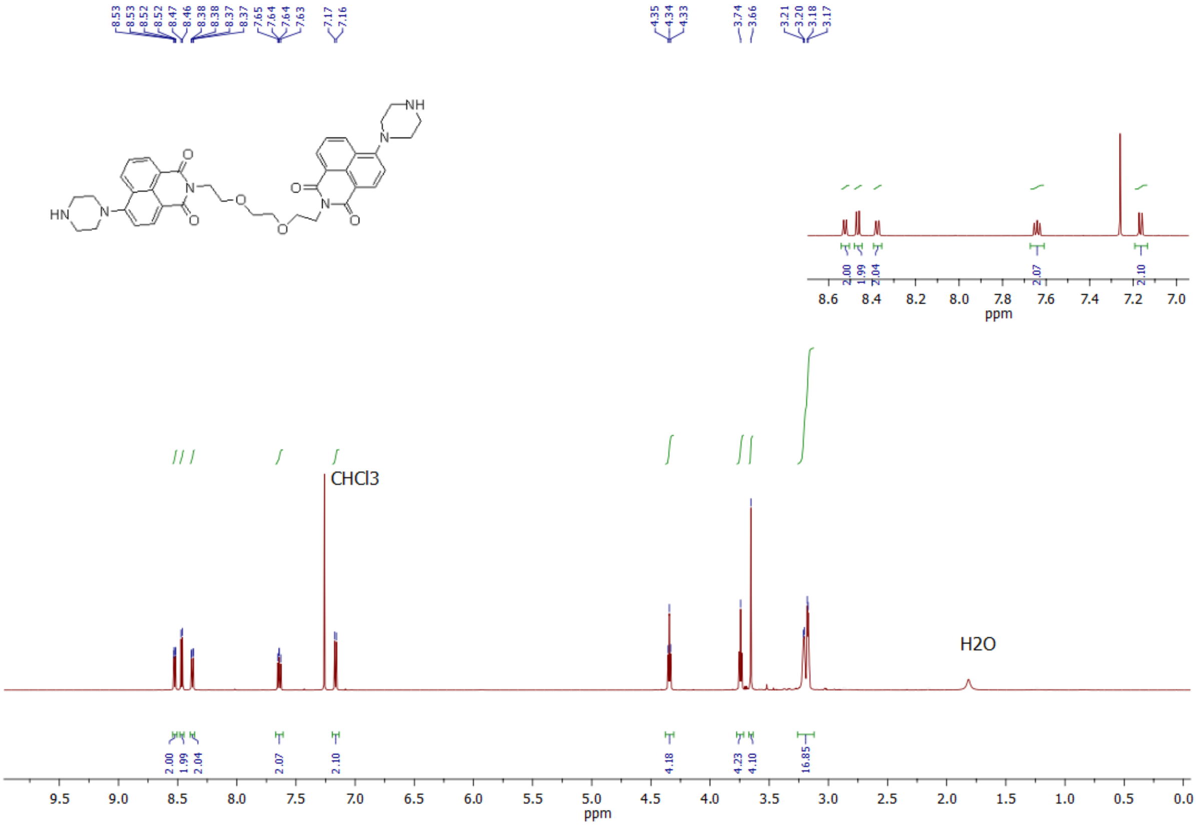

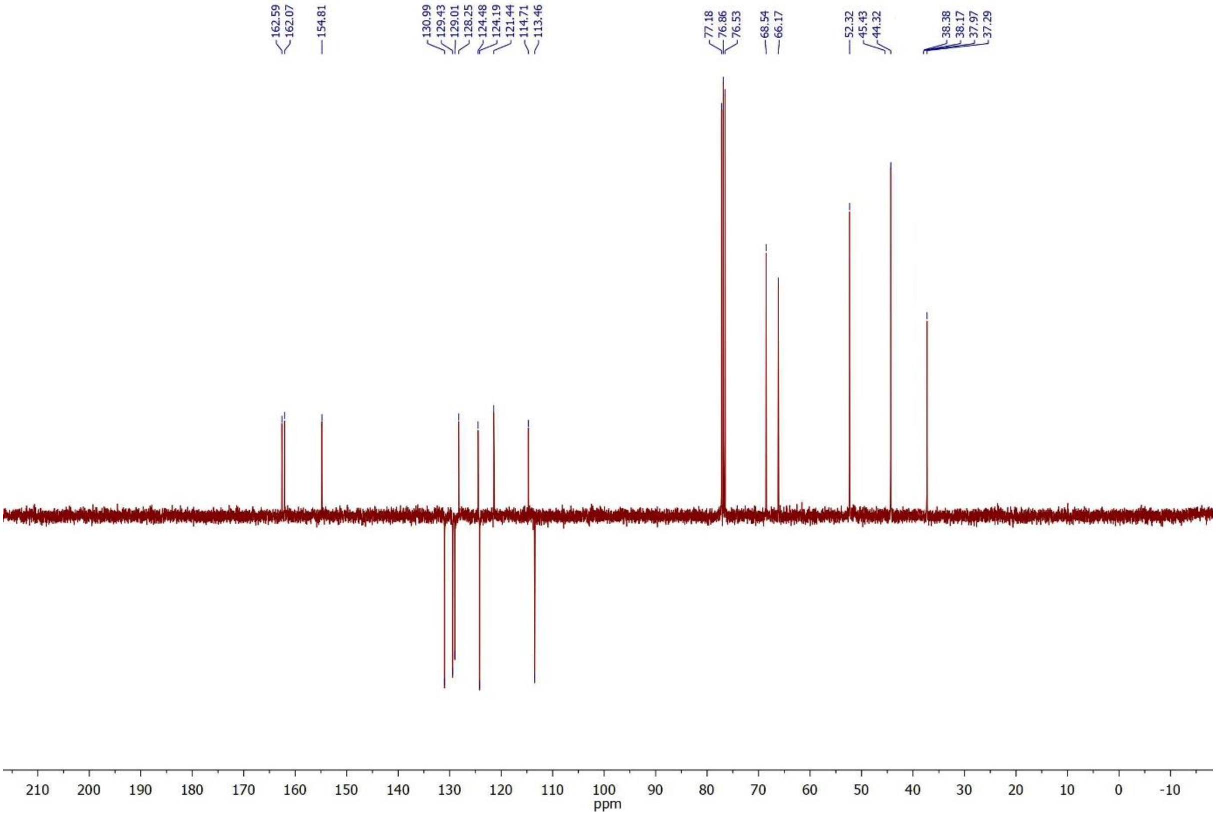

Figure S7. ^{1}H NMR and ^{13}C APT of **19**.

General procedure for the synthesis of receptors 1-6.

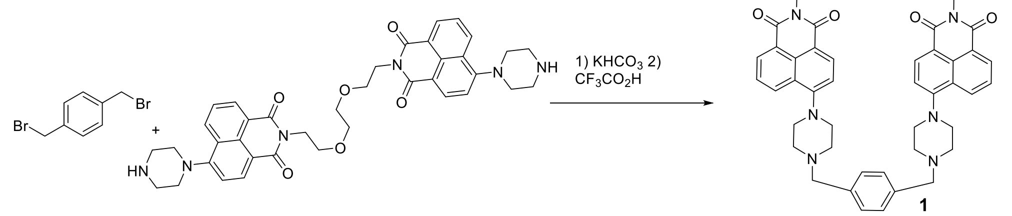

1

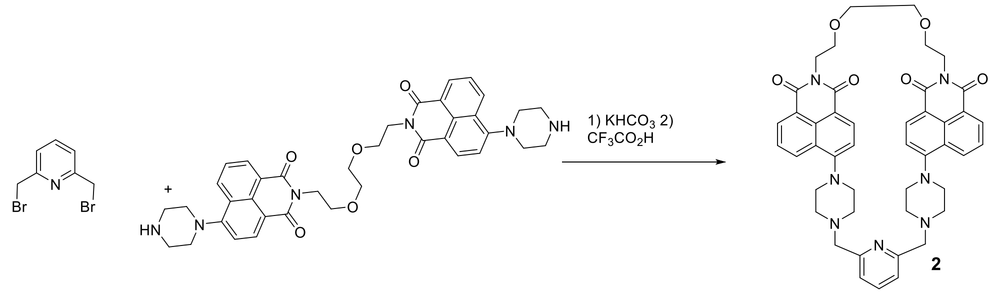

2

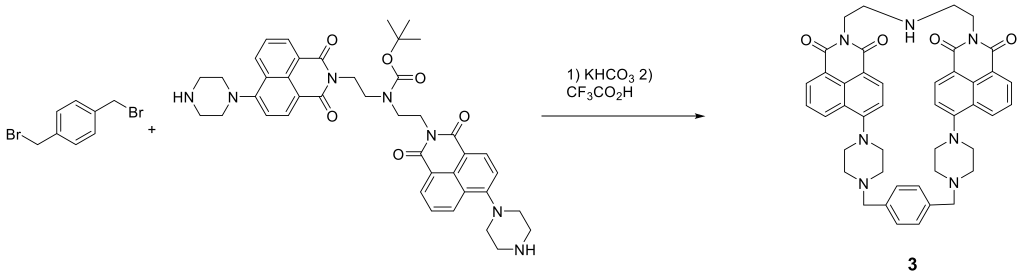

3

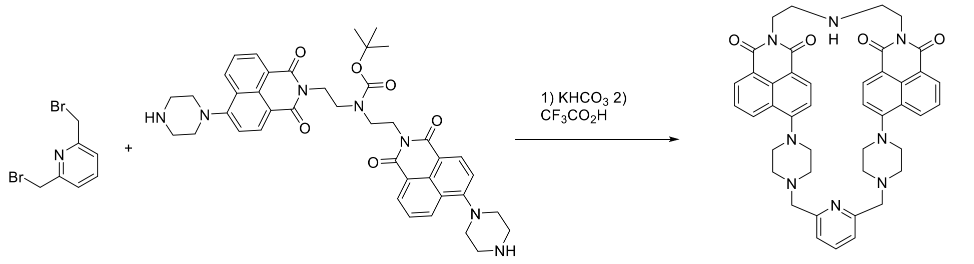

4

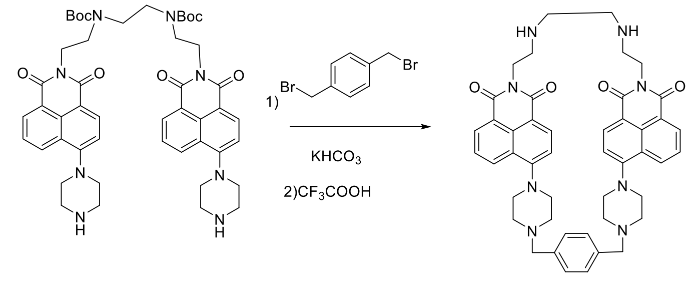

5

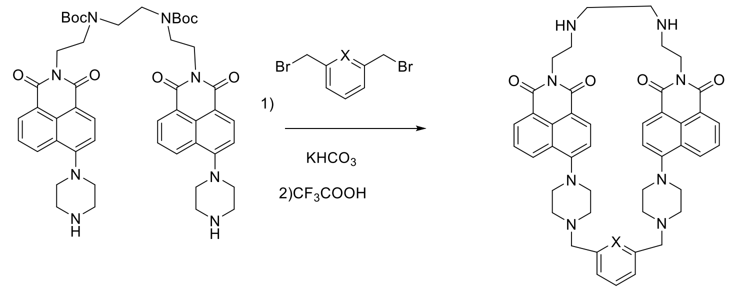

6, X=N
7, X=C

The diamine component (1 mmol) was dissolved in a mixture of acetonitrile (250 ml) and toluene (250 ml) at heating under nitrogen atmosphere and stirring. The solution was cooled to room temperature and K_2CO_3 (20 mmol) was added to the solution. Then the solution was heated at 90°C and during the next 5-6 hours corresponding dibromide (1 mmol) dissolved in 100 ml of toluene was added dropwise. Then the solution was heated further overnight. Acetonitrile was evaporated under vacuum. To the toluene solution in an extracting funnel, water (200 ml) and chloroform (200 ml) were added and shaked. The organic phase was separated, while the aqueous phase was extracted with chloroform. All organic phases were combined, dried over Na_2SO_4 and evaporated and dried in vacuum. The obtained solid was dissolved in 50 ml of chloroform and 5 ml of trifluoroacetic acid. The solution was left stirring overnight. After that the solution was poured into ice and neutralized with aqueous ammonia to pH 10-11. The obtained solution was extracted with a mixture of chloroform (40 ml) and methanol (10 ml) several times. The organic phase was dried over Na_2SO_4 and evaporated. The product was separated by column chromatography by using gradient elution $CHCl_3$-EtOH 200:5 to 200:50.

Receptor 1. Yield 39%. ^{1}H NMR (600 MHz, DMSO) δ 8.18 (d, *J* = 7.2 Hz, 2H), 8.12 (d, *J* = 8.1 Hz, 2H), 8.06 (d, *J* = 8.3 Hz, 2H), 7.49 – 7.44 (m, 2H), 7.40 (s, 4H), 7.13 (d, *J* = 8.2 Hz, 2H), 3.77 (s, 4H), 3.74 (s, 4H), 3.44 (t, *J* = 6.0 Hz, 4H), 3.40 (s, 4H), 3.31 (s, 8H), 2.44 (s, 8H). ^{13}C NMR (151 MHz, $CDCl_3$) δ 164.3, 163.5, 155.1, 131.9, 130.9, 130.2, 130.0, 130.0, 125.7, 125.0, 123.1, 115.7, 114.8, 70.3, 67.7, 62.3, 52.6, 50.5, 38.9. HRMS (ESI): m/z Calc. for $[M+H]^+$: $C_{46}H_{47}N_6O_6$ 779.3552, found 779.3550. Anal. Calcd for $C_{46}H_{46}N_6O_6$: C, 70.93; H, 5.95; N, 10.79. Found C, 70.71; H, 6.08; N, 10.77. M.p. 202.5 – 206.0°C.

Receptor 2. Yield 35%. ^{1}H NMR (600 MHz, $CDCl_3$) δ 8.34 (d, *J* = 7.2 Hz, 2H), 8.25 (d, *J* = 8.0 Hz, 2H), 8.08 (d, *J* = 8.3 Hz, 2H), 7.61 (s, 1H), 7.36 (t, *J* = 7.8 Hz, 2H), 7.14 (s, 2H), 6.90 (d, *J* = 8.0 Hz, 2H), 4.22 (t, *J* = 5.1 Hz, 4H), 3.86 (s, 4H), 3.64 (t, *J* = 5.2 Hz, 4H), 3.46 (s, 4H), 3.12 (s, 8H), 2.75 (s, 8H). ^{13}C NMR (101 MHz, $CDCl_3$) δ 164.2, 163.6, 155.5, 137.1, 132.3, 130.9, 129.8, 129.7, 125.7, 125.2, 123.2, 122.9, 116.5, 114.7, 70.3, 68.6, 63.3, 52.7, 52.5, 39.3.HRMS (ESI): m/z Calc. for $[M+H]^+$: $C_{45}H_{46}N_7O_6$ 780.3504, found 780.3505. Anal. Calcd for $C_{45}H_{45}N_7O_6$: C, 69.30; H, 5.82; N, 12.57. Found C, 69.12; H, 6.02; N, 12.52. M.p. 235.5 – 240.8°C.

Receptor 3. Yield 23%. ^{1}H NMR (600 MHz, $CDCl_3$) δ 8.22 (d, *J* = 7.9 Hz, 1H), 7.58 (d, *J* = 7.0 Hz, 1H), 7.41 (d, *J* = 8.2 Hz, 1H), 7.24 (s, 2H), 6.91 (d, *J* = 8.0 Hz, 1H), 6.63 (s, 1H), 4.15 (s, 2H), 3.96 (s, 2H), 3.06 (s, 6H), 2.55 (s, 4H), 1.50 (s, 3H). ^{13}C NMR (101 MHz, DMSO-d_6) δ 13C NMR (101 MHz, CDCl3) δ 155.8, 154.9, 146.3, 123.2, 122.0, 121.1, 120.8, 120.2, 119.6, 115.9, 115.1, 112.2, 106.6, 105.5, 68.5, 68.2, 67.9, 54.2, 52.0, 41.2, 38.9, 36.7, 29.6, 20.5, 8.2. HRMS (ESI): m/z Calc. for $[M+H]^+$: $C_{44}H_{44}N_7O_4$ 734.3455, found 734.3450. Anal Calcd for $C_{44}H_{43}N_7O_4$: C, 72.01; H, 5.91; N, 13.36. Found C, 71.88; H, 6.12; N, 13.28. M.p. 179.3 – 181.5°C.

Receptor 4. Yield 17%. ^{1}H NMR (600 MHz, $CDCl_3$) δ 8.27 (d, *J* = 7.1 Hz, 7H), 8.01 (t, *J* = 7.8 Hz, 14H), 7.63 (t, *J* = 7.7 Hz, 4H), 7.34 (t, *J* = 7.8 Hz, 7H), 7.15 (d, *J* = 7.7 Hz, 7H), 6.85 (d, *J* = 8.0 Hz, 7H), 4.31 – 4.20 (m, 15H), 3.96 (s, 14H), 3.68 (q, *J* = 7.0 Hz, 12H), 3.07 (s, 29H), 3.00 – 2.94 (m, 16H), 2.91 (s, 28H). ^{13}C NMR (101 MHz, $CDCl_3$) δ 168.7, 168.5, 161.1, 159.8, 141.4, 136.2, 134.9, 134.1, 133.3, 129.4, 129.3, 127.1, 126.2, 119.8, 118.5, 66.1, 56.9, 56.0, 53.1, 52.9, 52.7, 52.5, 52.2, 52.0, 51.8, 51.2, 33.5. HRMS (ESI): m/z Calc. for $[M+H]^+$: $C_{43}H_{43}N_8O_4$ 736.3485, found 734.3488. Anal. Calcd for $C_{43}H_{42}N_8O_4$: C, 70.28; H, 5.76; N, 15.25. Found C, 70.01; H, 5.54; N, 15.20. M.p. 186.0 – 190.0°C.

Receptor 5. Yield 20%. ^{1}H NMR (601 MHz, DMSO-d_6) δ 8.21 (d, *J* = 8.0 Hz, 2H), 8.11 (t, 4H), 7.50 (t, 2H), 7.20 (s, 4H), 7.12 (d, *J* = 8.2 Hz, 2H), 3.79 – 3.67 (m, 8H), 2.65 (s, 4H), 2.42 (s, 7H). ^{13}C NMR (151 MHz, DMSO-d_6) δ 164.1, 163.3, 155.4, 134.1, 132.0, 130.8, 130.8, 130.1, 129.7, 125.8, 125.4, 122.9, 115.3, 114.9, 61.7, 52.8, 50.5, 48.4,47.0. HRMS (ESI): m/z Calc. for [M+H]+: $C_{46}H_{49}N_8O_4$ 777.3877, found 777.3880. Anal. Calcd for $C_{46}H_{48}N_8O_4$: C, 71.11; H, 6.23; N, 14.42. Found C, 70.96; H, 5.98; N, 14.39. M.p. 198.5 – 201.0°C with decomposition.

Receptor 6. Yield 45%. ^{1}H NMR (400 MHz, DMSO-d6) δ 8.46 – 8.10 (m, 1H), 7.98 (t, J = 7.8 Hz, 0H), 7.76 – 7.39 (m, 1H), 7.16 (d, J = 8.1 Hz, 0H), 4.66 (s, 1H), 3.78 – 2.87 (m, 5H). ^{13}C NMR (101 MHz, DMSO-d6) δ 165.4, 164.9, 154.2, 149.9, 133.0, 131.9, 130.9, 129.0, 125.2, 122.0, 116.5, 115.8, 52.3, 52.2, 52.2, 49.6, 49.6, 44.4, 39.0, 38.8, 38.6, 38.3, 38.1, 37.9, 37.7. HRMS (ESI): m/z Calc. for $[M+H]^{+}$: $C_{45}H_{48}N_{9}O_{4}$ 778.3829, found 778.3830. Anal. Calcd for $C_{45}H_{48}N_{9}O_{4}$: C, 69.48; H, 6.09; N, 16.20. Found C, 69.33; H, 5.93; N, 16.12. M.p. 215.0 – 218.0°C.

Receptor 7: Yield 12%. ^{1}H NMR (601 MHz, DMSO-d_6) δ 8.31 – 8.21 (m, 6H), 7.68 (s, 1H), 7.61 – 7.55 (m, 2H), 7.28 (t, *J* = 7.6 Hz, 1H), 7.23 (d, *J* = 8.1 Hz, 2H), 7.13 (d, *J* = 7.3 Hz, 2H), 4.19 (s, 4H), 3.71 (s, 4H), 3.05 (d, *J* = 47.3 Hz, 12H), 2.81 (s, 4H), 2.64 (s, 9H). ^{13}C NMR (151 MHz, DMSO-d_6) δ 164.0, 163.4, 157.8, 155.6, 137.9, 132.0, 130.5, 129.0, 128.0, 127.7, 127.4, 126.0, 124.9, 122.6, 118.3, 116.4, 115.7, 114.7, 69.8, 60.9, 52.9, 52.0, 47.6. HRMS (ESI): m/z Calc. for [M+H]+: $C_{46}H_{49}N_{8}O_{4}$ 777.3877, found 777.3874. Anal. Calcd for $C_{46}H_{48}N_{8}O_{4}$: C, 71.11; H, 6.23; N, 14.42. Found C, 70.96; H, 5.98; N, 14.39. M.p. 201 – 202°C with decomposition.

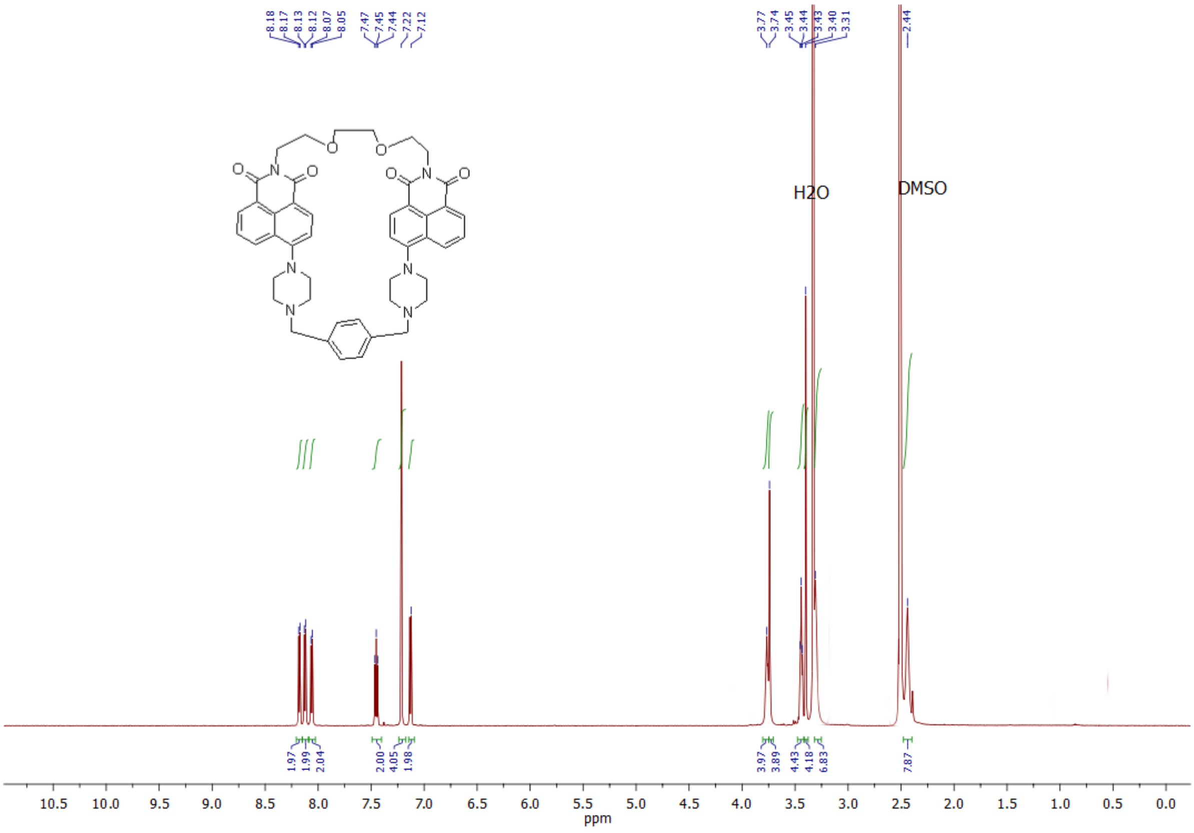

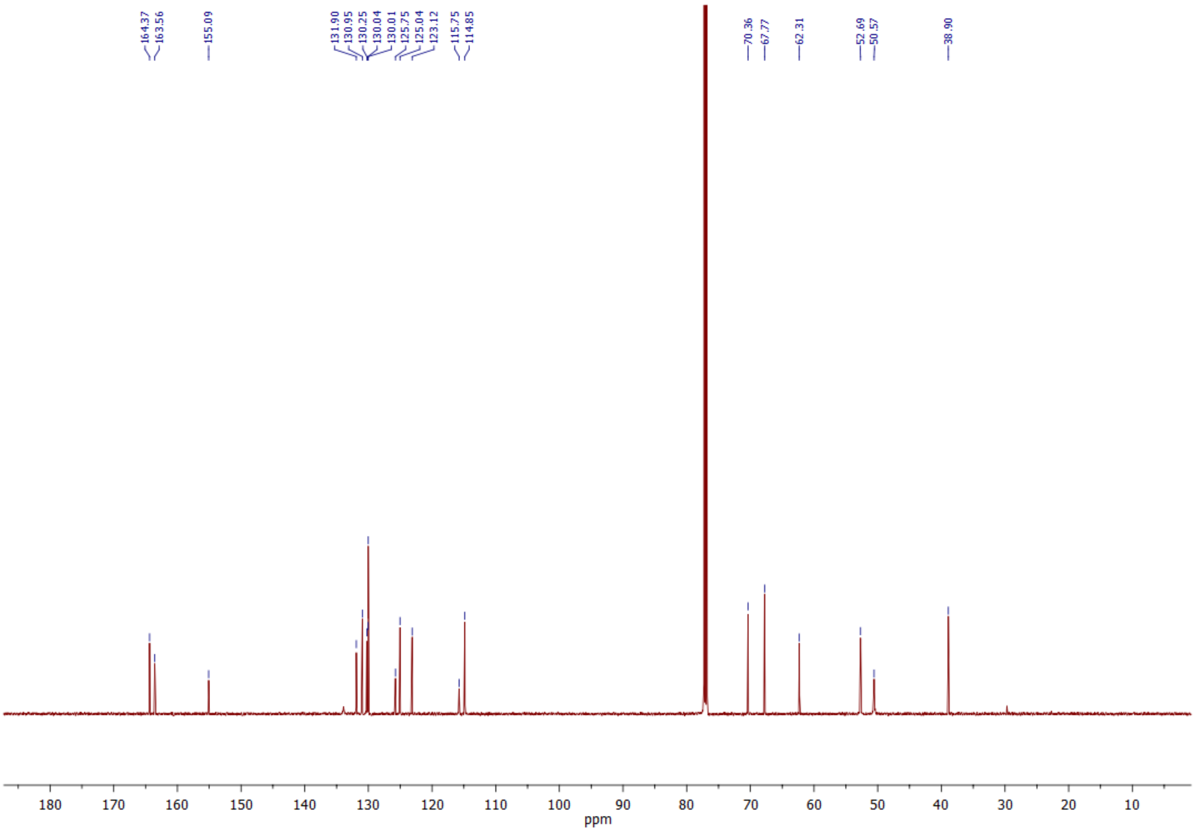

Figure S8. ^{1}H and ^{13}C NMR for **1**.

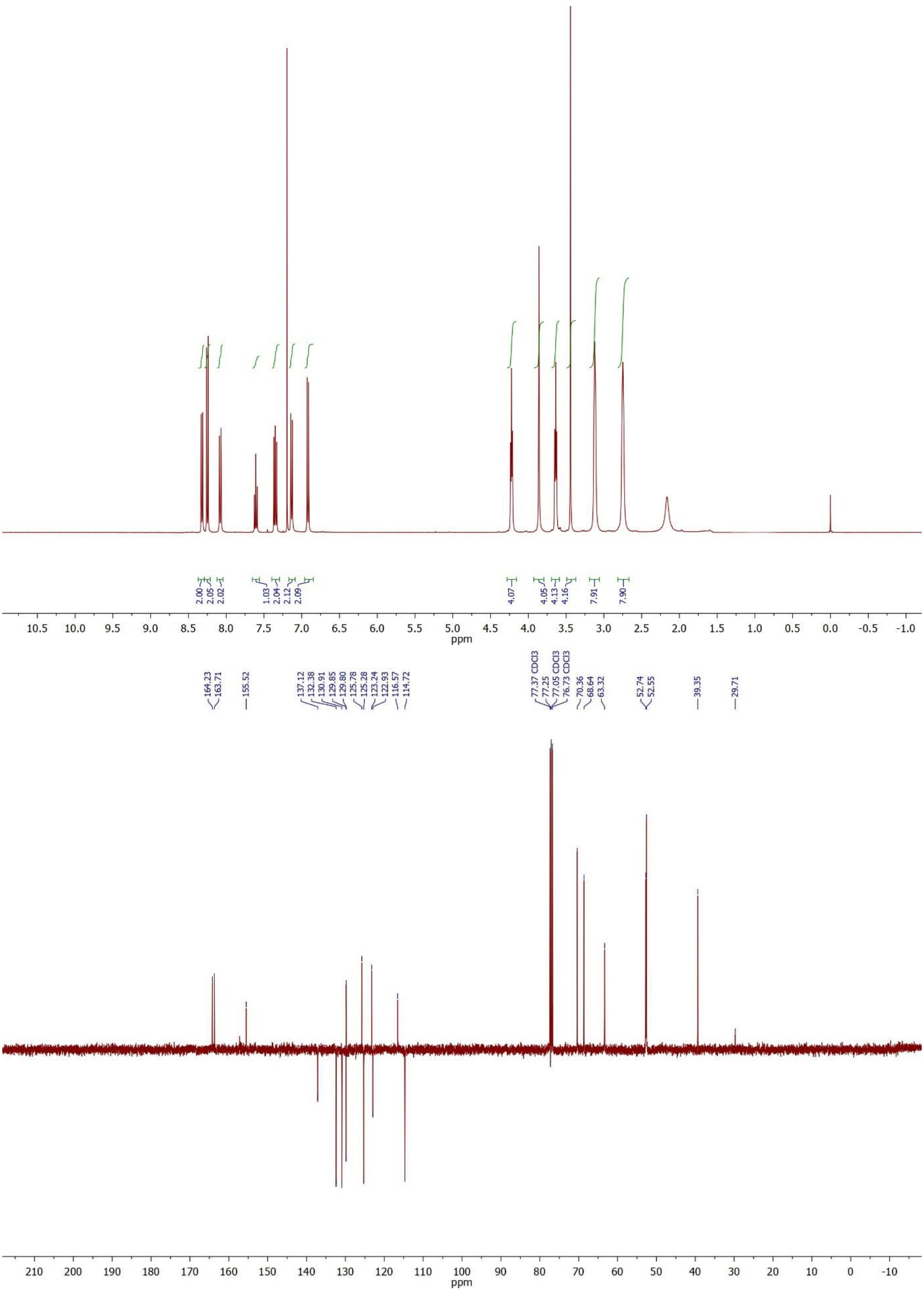

Figure S9. ^{1}H and ^{13}C APT NMR for **2**.

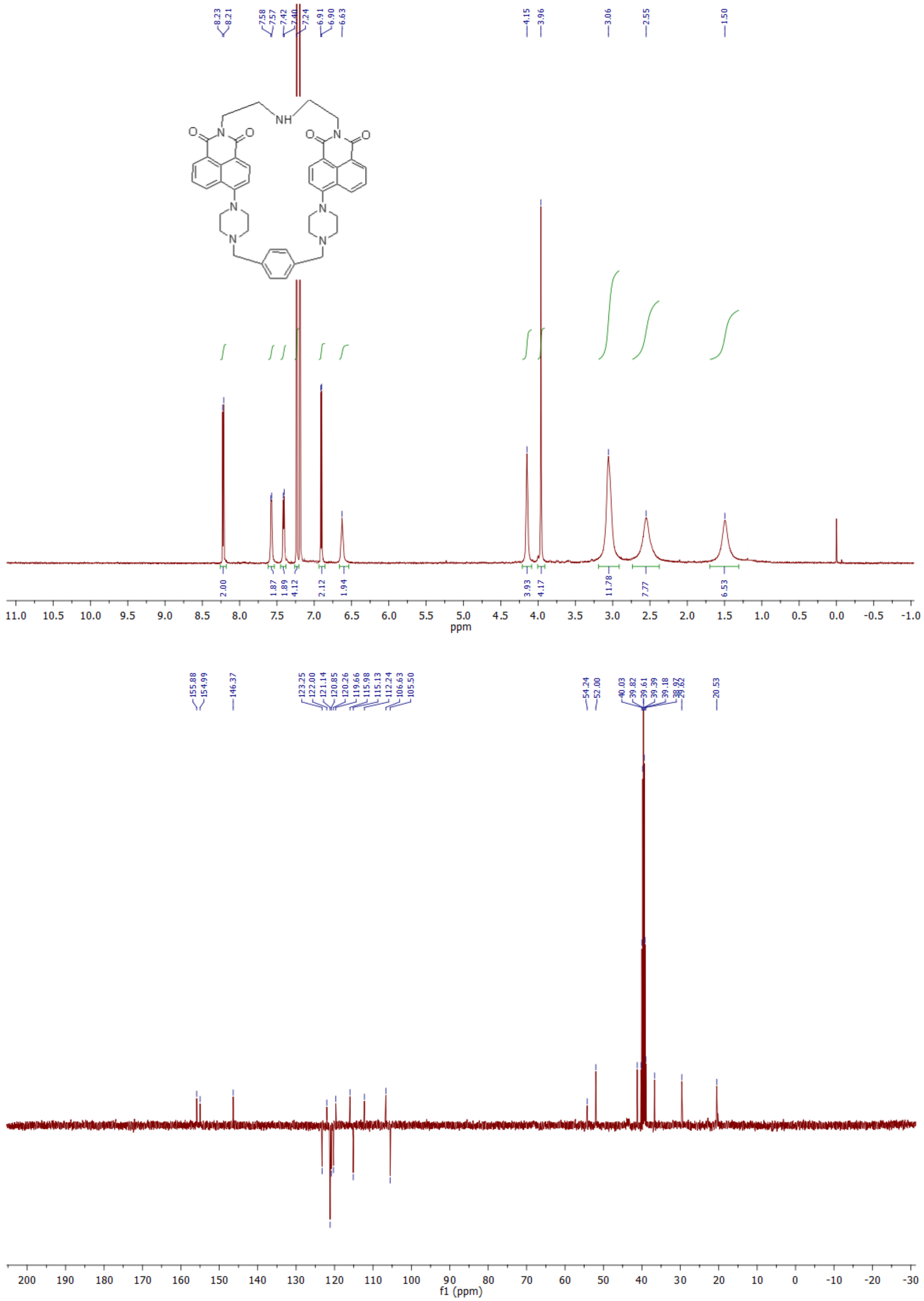

Figure S10. ^{1}H and ^{13}C NMR for **3**.

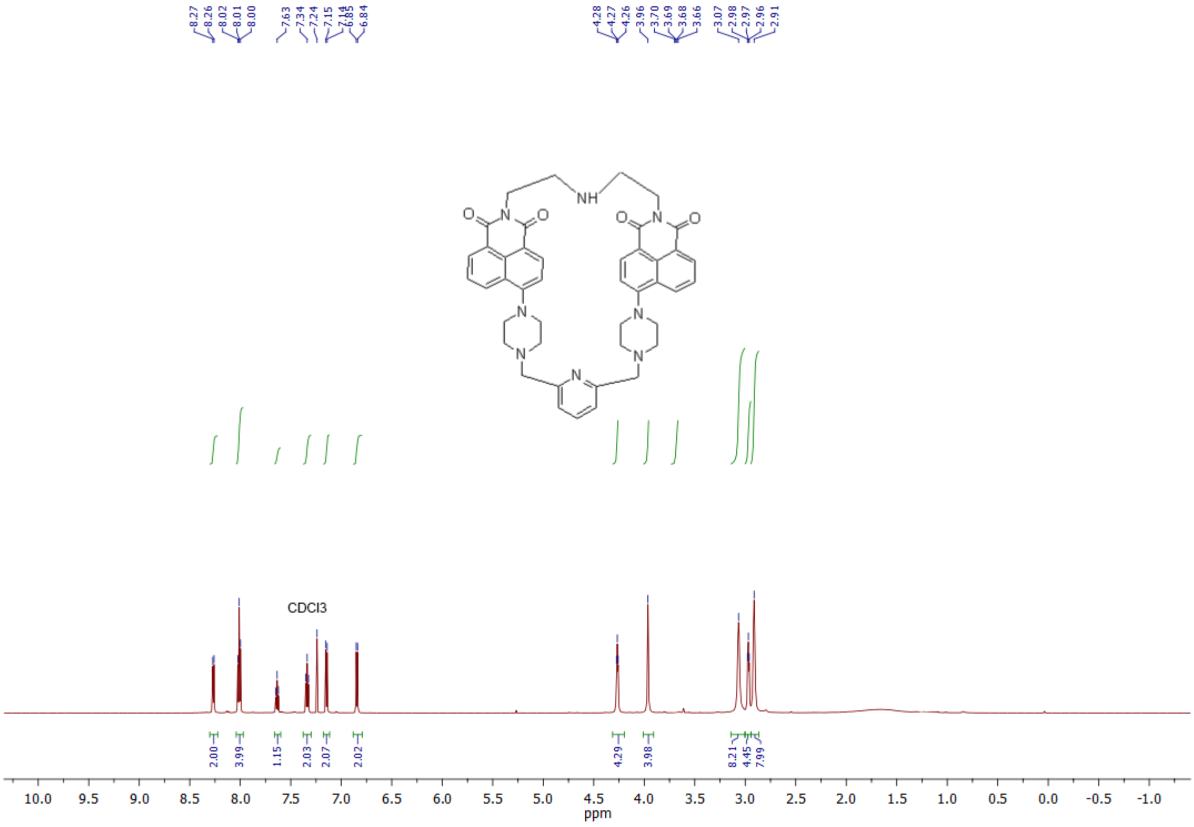

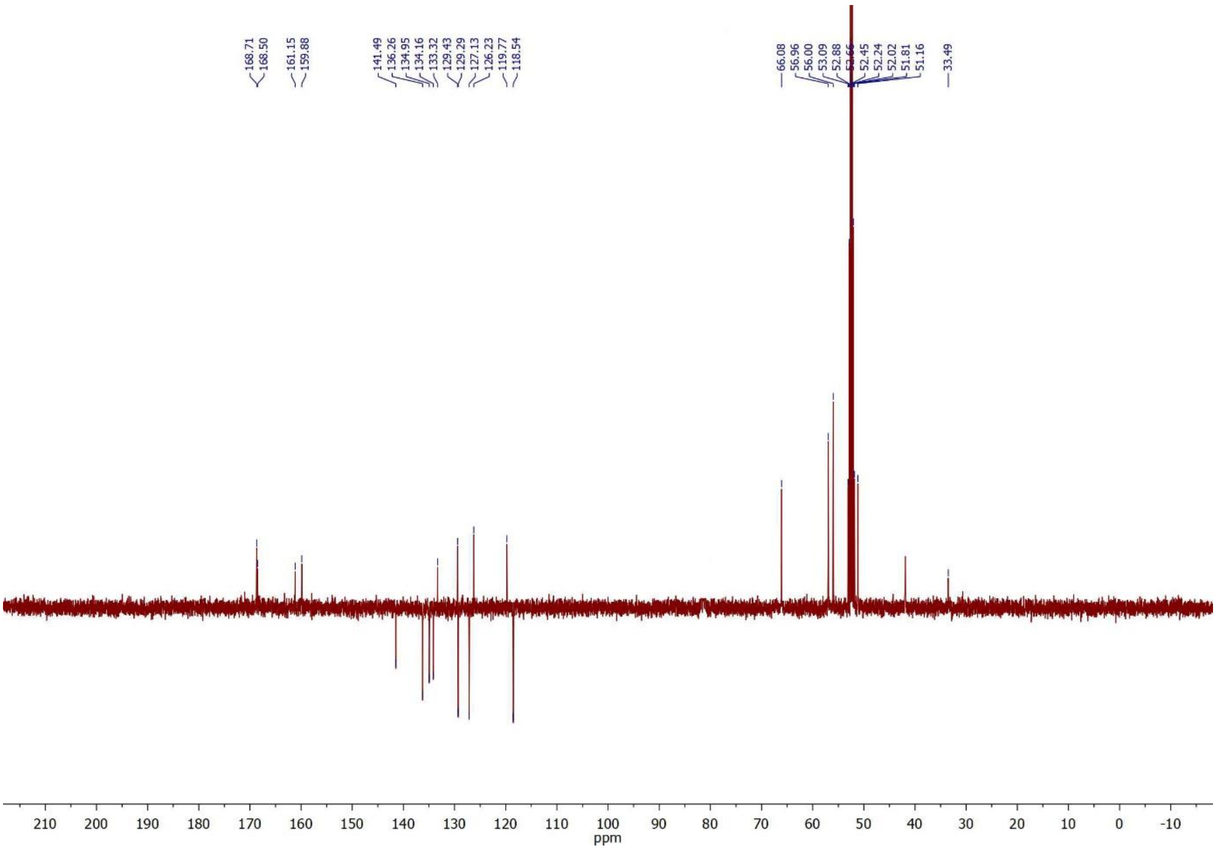

Figure S11. ^{1}H and ^{13}C NMR for **4**.

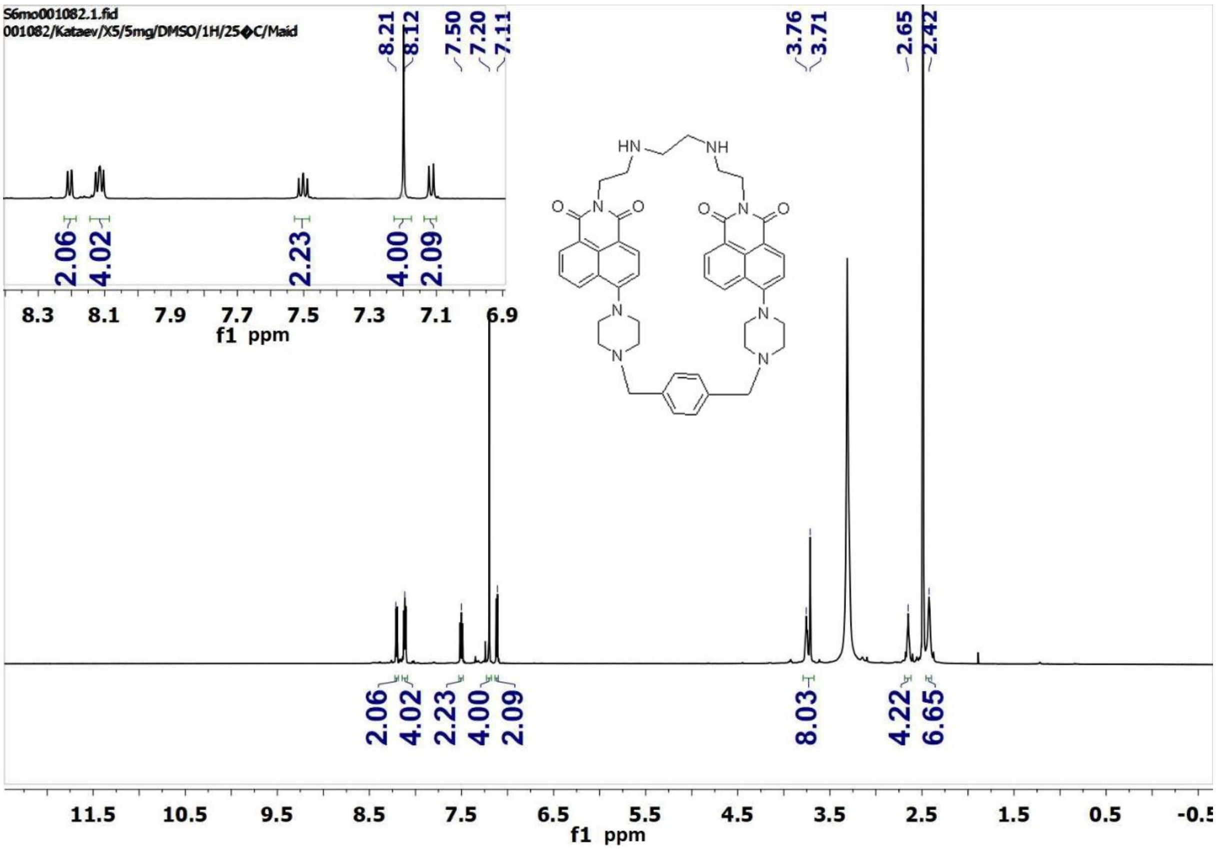

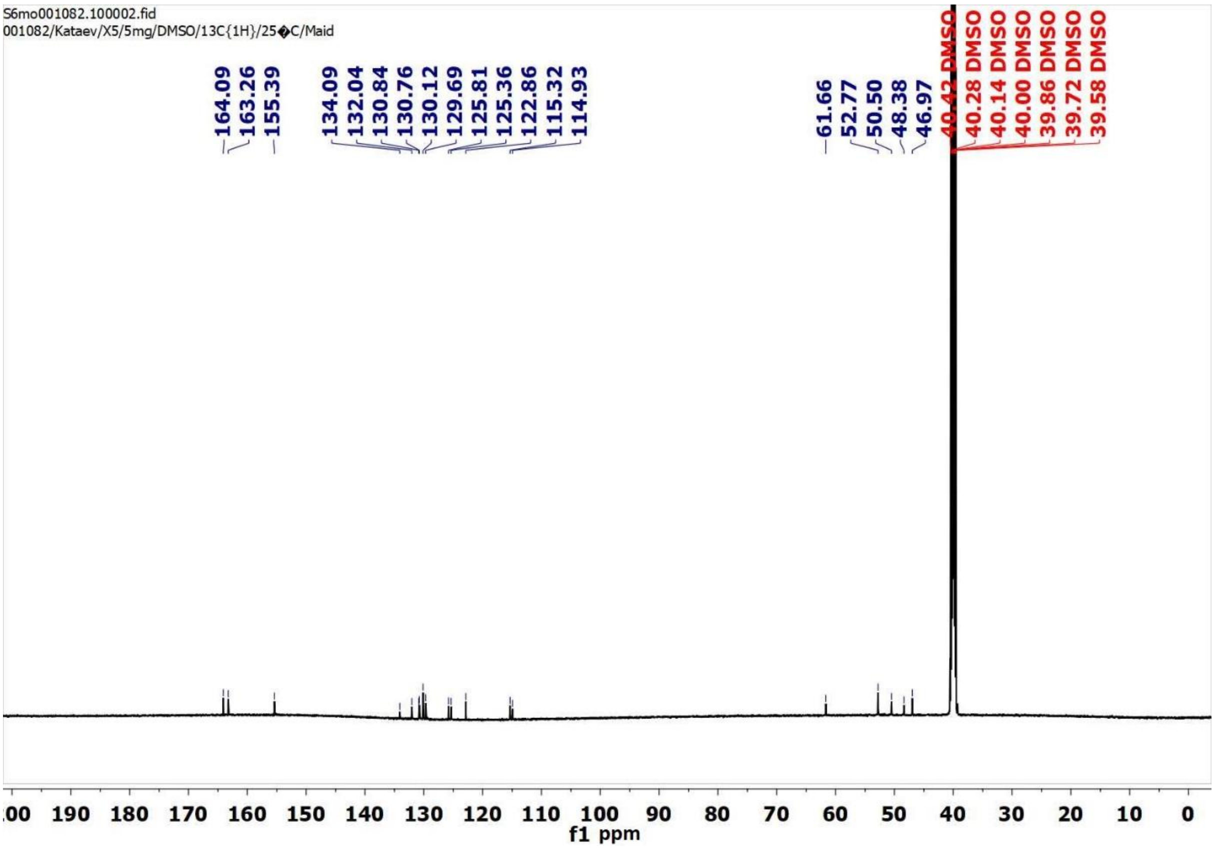

Figure S12. ^{1}H and ^{13}C NMR for **5**

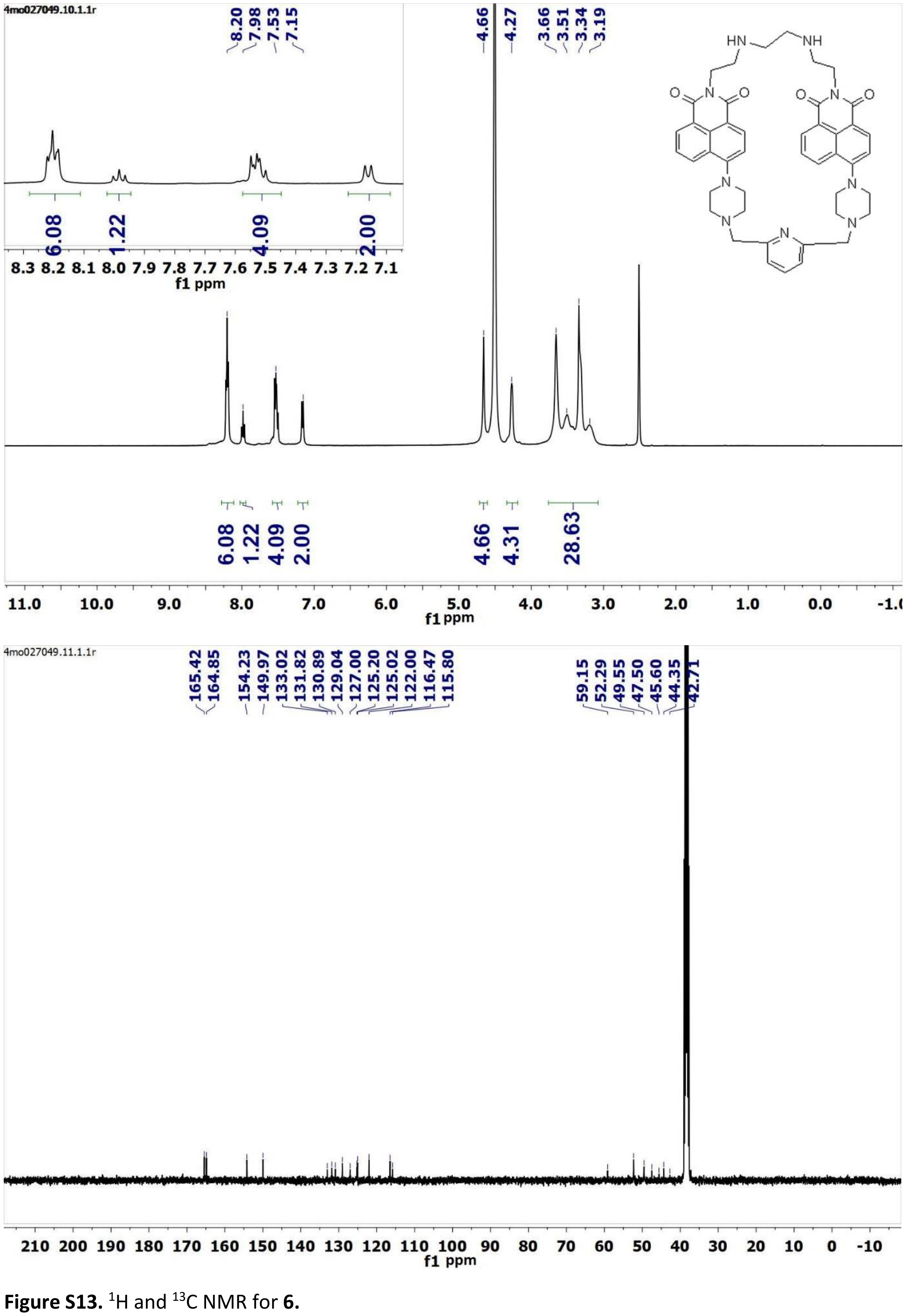

Figure S13. ^{1}H and ^{13}C NMR for **6.**

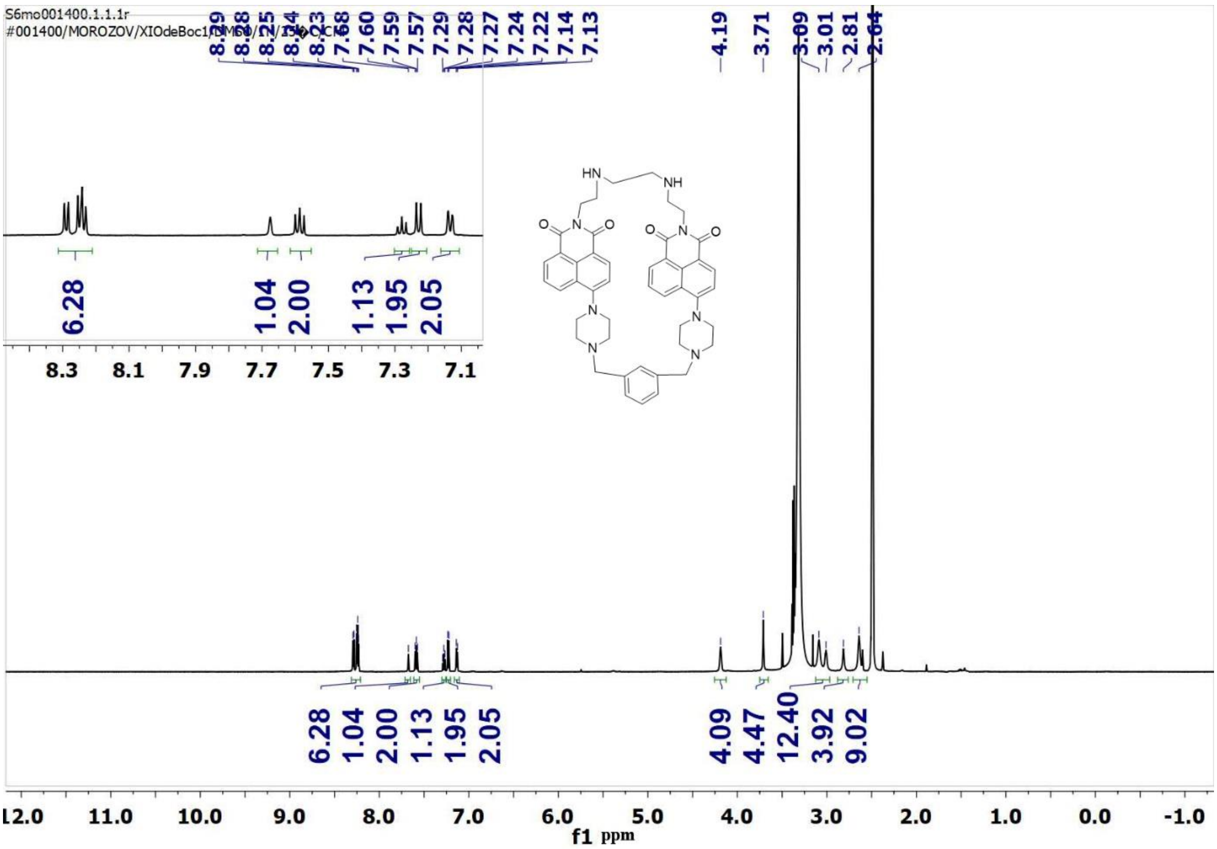

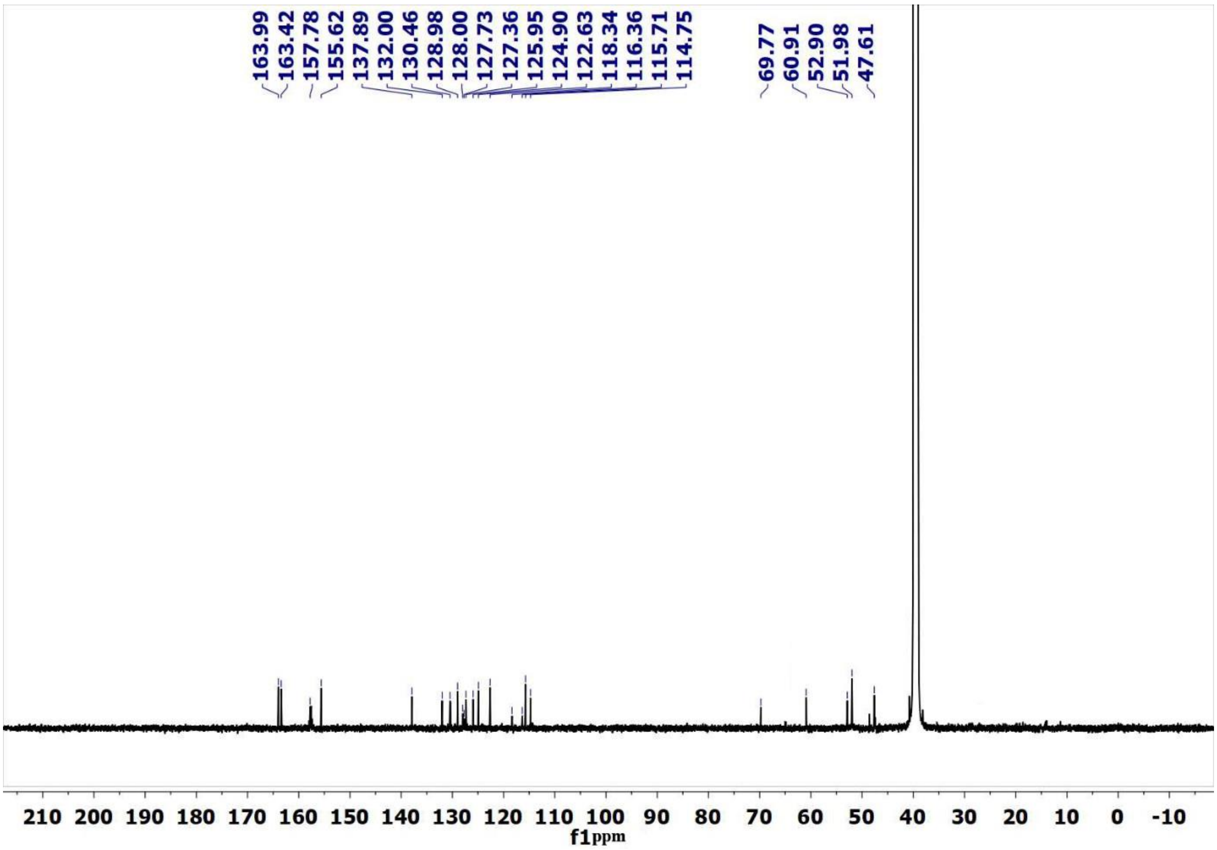

Figure S14. ^{1}H and ^{13}C NMR for **7.**

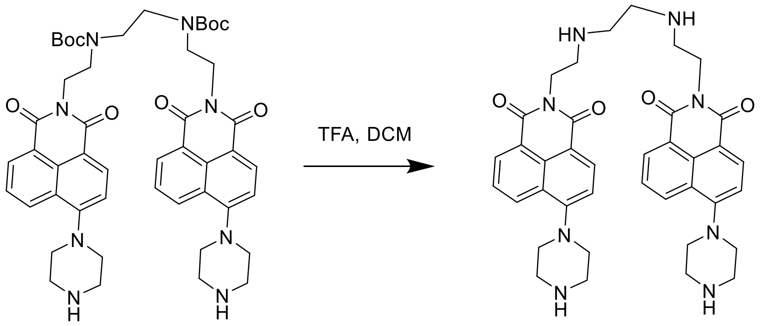

10

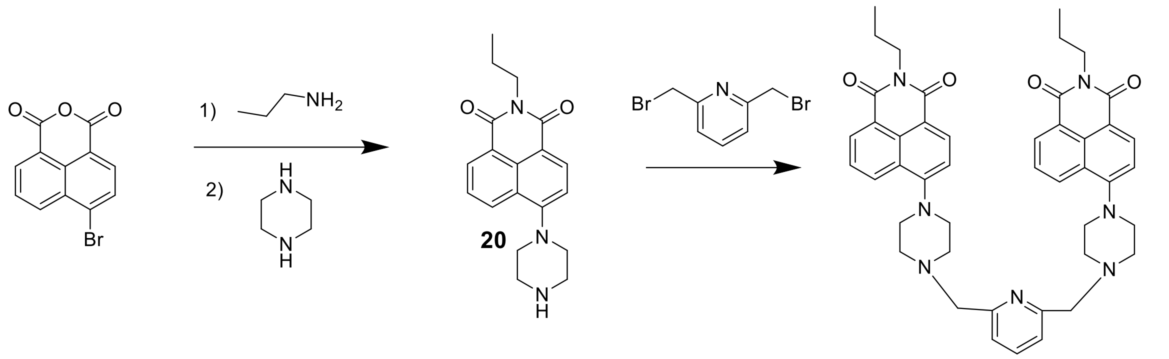

11

Compound **10**. Compound **17** (0,1 mmol) was diluted in 10 ml of dry DCM and concentrated trifluoroacetic acid (1mmol, 76mkl) was added to the solution at once. The mixture was stirred overnight and then the solvent with TFA were evaporated on vacuum. The solid residue was again diluted and evaporated on vacuum in order to remove the remaining TFA. Yield 95%. ^{1}H NMR (400 MHz, DMSO-d_6) δ 8.50 – 8.44 (m, 4H), 8.40 (d, J = 8.1 Hz, 2H), 7.80 (t, J = 7.9 Hz, 2H), 7.37 (d, J = 8.2 Hz, 2H), 4.34 (s, 4H), 3.52 – 3.24 (m, 25H). ^{13}C NMR (101 MHz, DMSO-d_6) δ 165.4, 164.9, 155.3, 133.2, 132.0, 131.4, 129.4, 127.1, 125.6, 122.2, 116.4, 116.2, 66.6, 49.6, 46.9, 43.5, 43.2, 38.3, 38.0, 37.8, 36.8. HRMS (ESI): m/z Calc. for $[M+H]^+$: $C_{38}H_{43}N_8O_4$ 675.3407, found 675.3411. M.p. 311.0 – 315.0^{o}C (TFA salt).

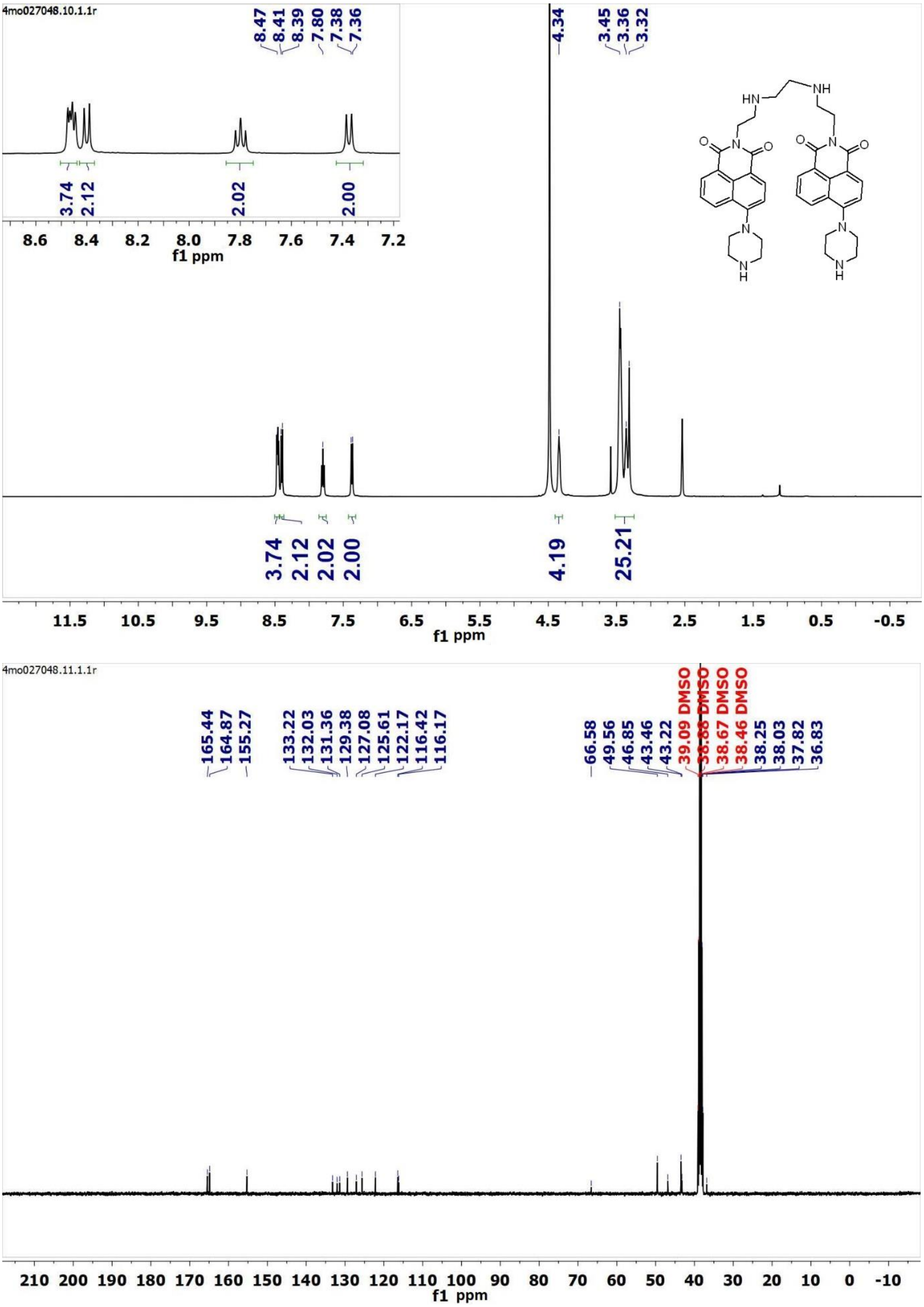

Figure S15. ^{1}H and ^{13}C NMR for **10**.

Compound **20**. The solution of the propyl amine (8 mmol) in 5 ml of ethanol was added to the suspension of 1,8-naphthalic anhydride (2,08 g, 7,5 mmol) in 120 ml of ethanol. The obtained

mixture was refluxed for 10 h and poured in 200 ml of water. The precipitate was filtered off and dried under vacuum. The obtained precipitate was placed in a 100 ml flask together with piperazine (821 mg, 9.53 mmol) and 30 ml of 2-methoxyethanol. The reaction was stirred at 90°C overnight until the starting material disappeared (the reaction was controlled by TLC). The solvent was removed under reduced pressure, then dried in vacuum and the product was purified by column chromatography by using chloroform-ethanol mixture 1:1 to wash off the minor byproducts. The product stayed on the column and then was washed with chloroform-ethanol-aqueous ammonia solution (100:100:5 vol.). After the column chromatography the product was dried in vacuum to complete dryness. Yield 78%. ^{1}H NMR (400 MHz, DMSO-d6) δ 8.46 (d, J = 7.8 Hz, 2H), 8.39 (d, J = 8.1 Hz, 1H), 7.80 (t, J = 7.9 Hz, 1H), 7.37 (d, J = 8.1 Hz, 1H), 4.00 – 3.93 (m, 3H), 3.37 – 3.25 (m, 8H), 1.62 (q, J = 7.4 Hz, 2H), 0.89 (t, J = 7.4 Hz, 3H). ^{13}C NMR (101 MHz, DMSO-d6) δ 172.9, 164.1, 163.6, 155.4, 132.6, 131.3, 130.9, 129.4, 126.8, 125.8, 122.9, 116.7, 116.0, 51.0, 43.8, 41.5, 21.3, 21.3, 21.1, 20.9, 20.7, 20.5, 20.3,11.8, (additional signals appear due to addition of AcOD-d4 to the sample). HRMS (ESI): m/z Calc. for $[M+H]^{+}$: $C_{19}H_{22}N_{3}O_{2}$ 324.1712, found 324.1715. M.p. 286.0 – 289.0°C.

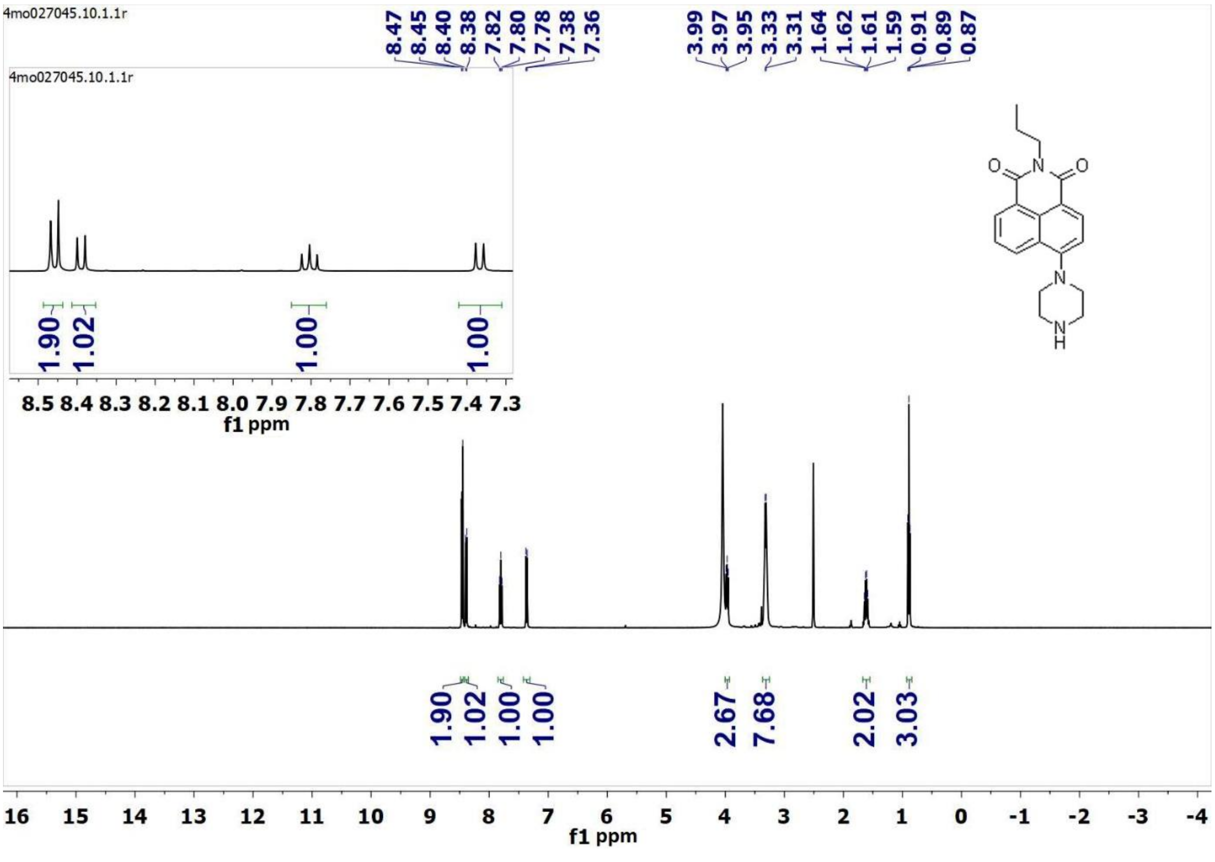

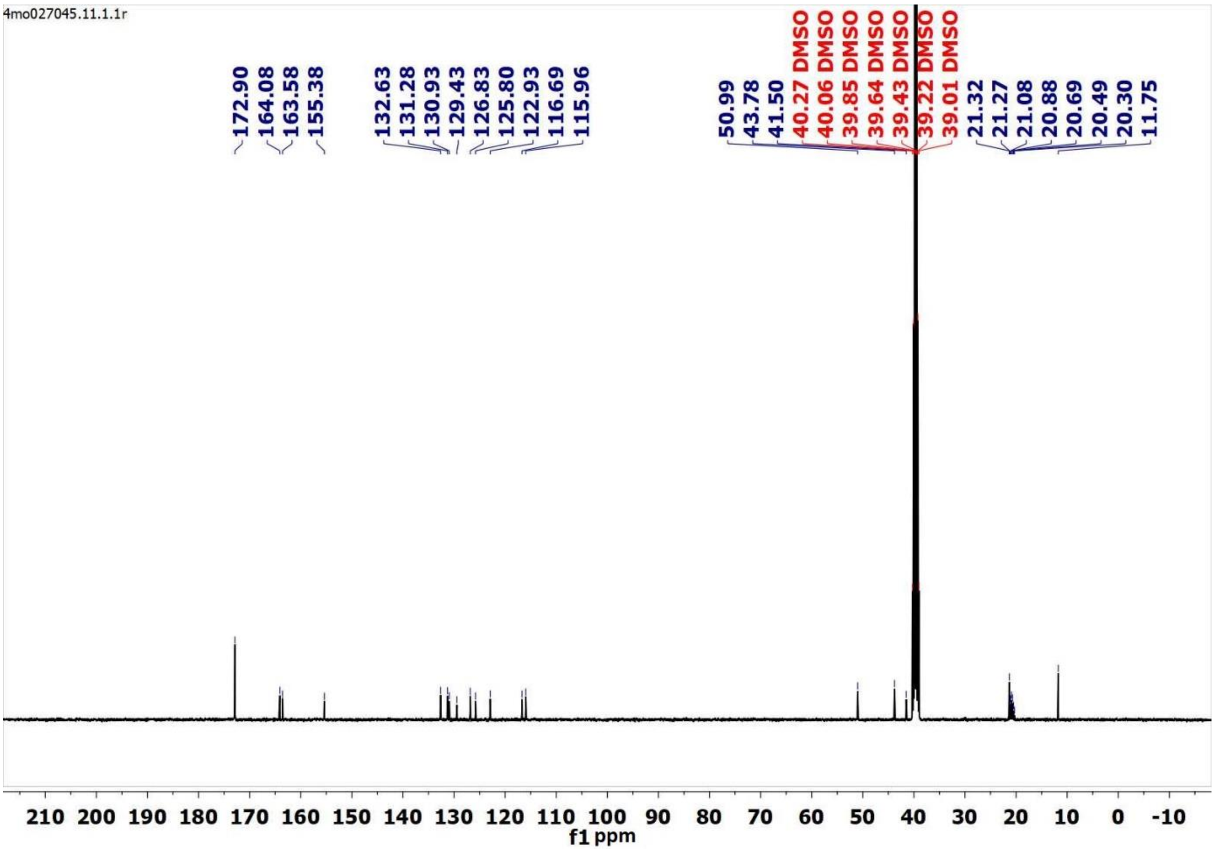

Figure S16. ^{1}H and ^{13}C NMR for **21.**

Compound **11**. Compound **20** (2,1 mmol) was dissolved in a mixture of acetonitrile (150 ml) and toluene (100 ml) at heating under nitrogen atmosphere and stirring. Then K_2CO_3 (20 mmol) was added to the solution. Then the solution was heated at 90°C and pyridine dibromide compound (1 mmol) dissolved in 30 ml of toluene was added at once. Then the solution was heated further overnight. Acetonitrile was evaporated under vacuum. To the toluene solution in an extracting funnel, water (200 ml) and chloroform (200 ml) were added and shaked. The organic phase was separated, while the aqueous phase was extracted with chloroform. All organic phases were combined, dried over Na_2SO_4 and evaporated and dried in vacuum. The product was separated by column chromatography by using gradient elution CH_2Cl_2-MeOH 200:1 to 200:6. Yield 70%. ^{1}H NMR (400 MHz, DMSO-d6) δ 8.48 – 8.37 (m, 6H), 7.85 – 7.74 (m, 3H), 7.42 (d, J = 7.7 Hz, 2H), 7.34 (d, J = 8.2 Hz, 2H), 4.05 – 3.94 (m, 4H), 3.77 (s, 4H), 3.28 (s, 8H), 2.80 (s, 8H), 1.64 (q, J = 7.4 Hz, 4H), 0.91 (t, J = 7.4 Hz, 6H). ^{13}C NMR (101 MHz, DMSO-d6) δ 165.4, 164.9, 154.2, 149.8, 133.3, 131.8, 130.9, 129.0, 127.0, 125.2, 125.0, 122.0, 116.5, 115.8, 59.2, 52.3, 52.3, 49.6, 49.6, 47.5, 47.5, 45.6, 44.3,44.1. HRMS (ESI): m/z Calc. for $[M+H]^+$: $C_{45}H_{48}N_7O_4$ 750.3768, found 750.3774. M.p. 139.2 – 143.5°C.

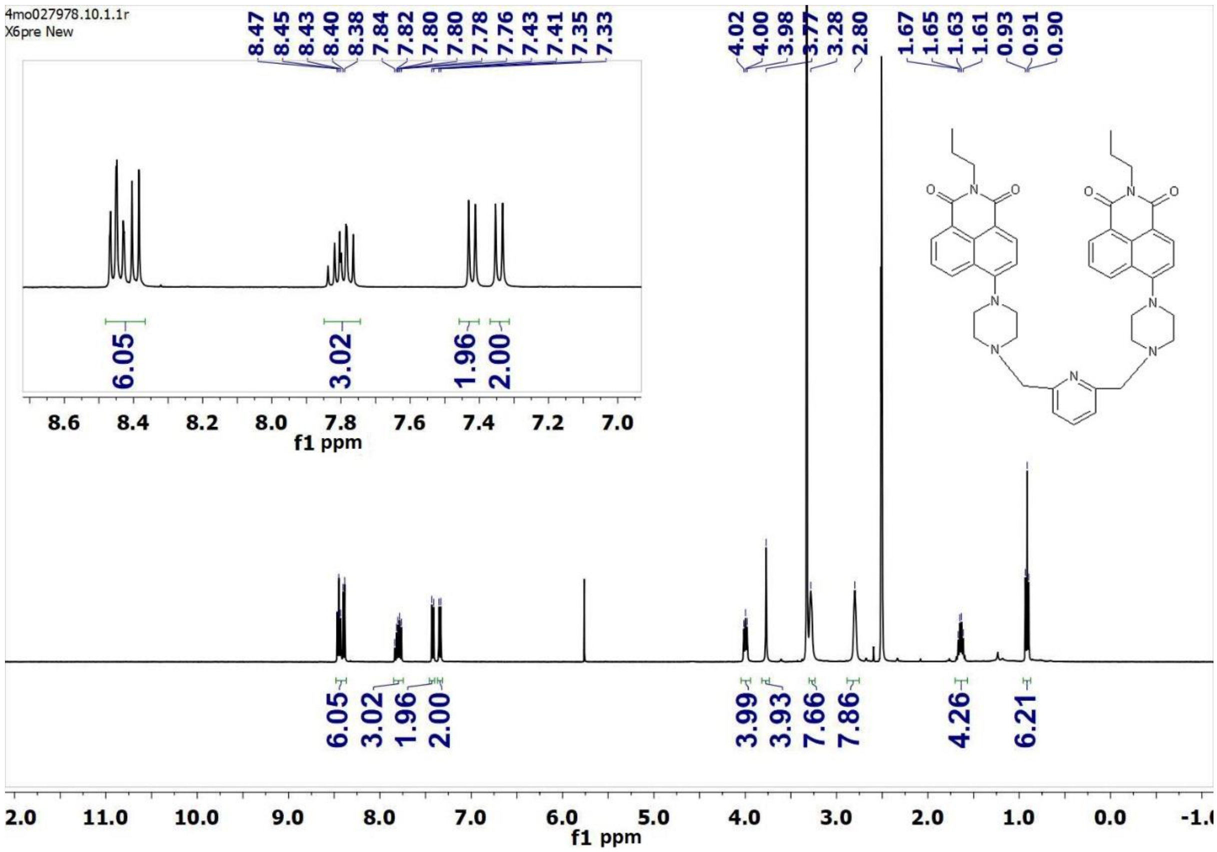

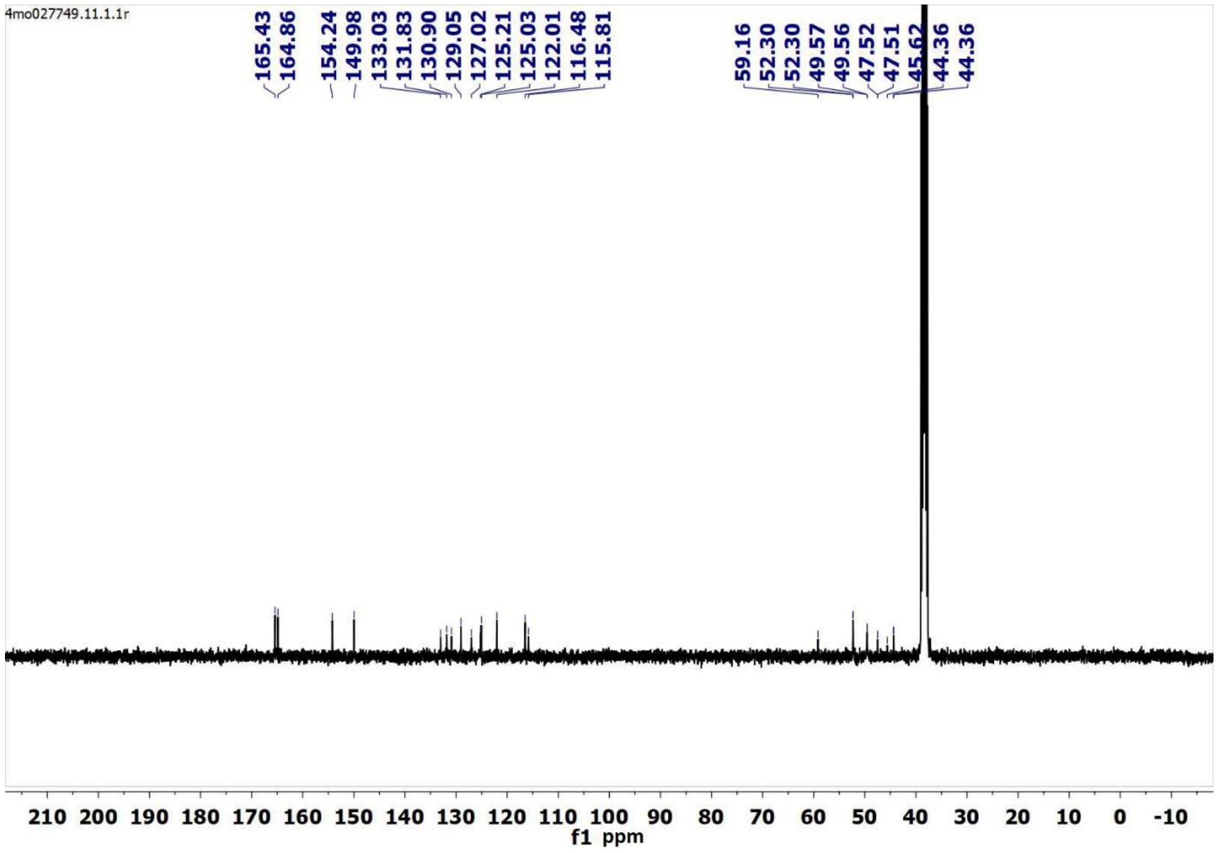

Figure S17. ^{1}H and ^{13}C NMR for **11.**

Fluorescence-pH relationship

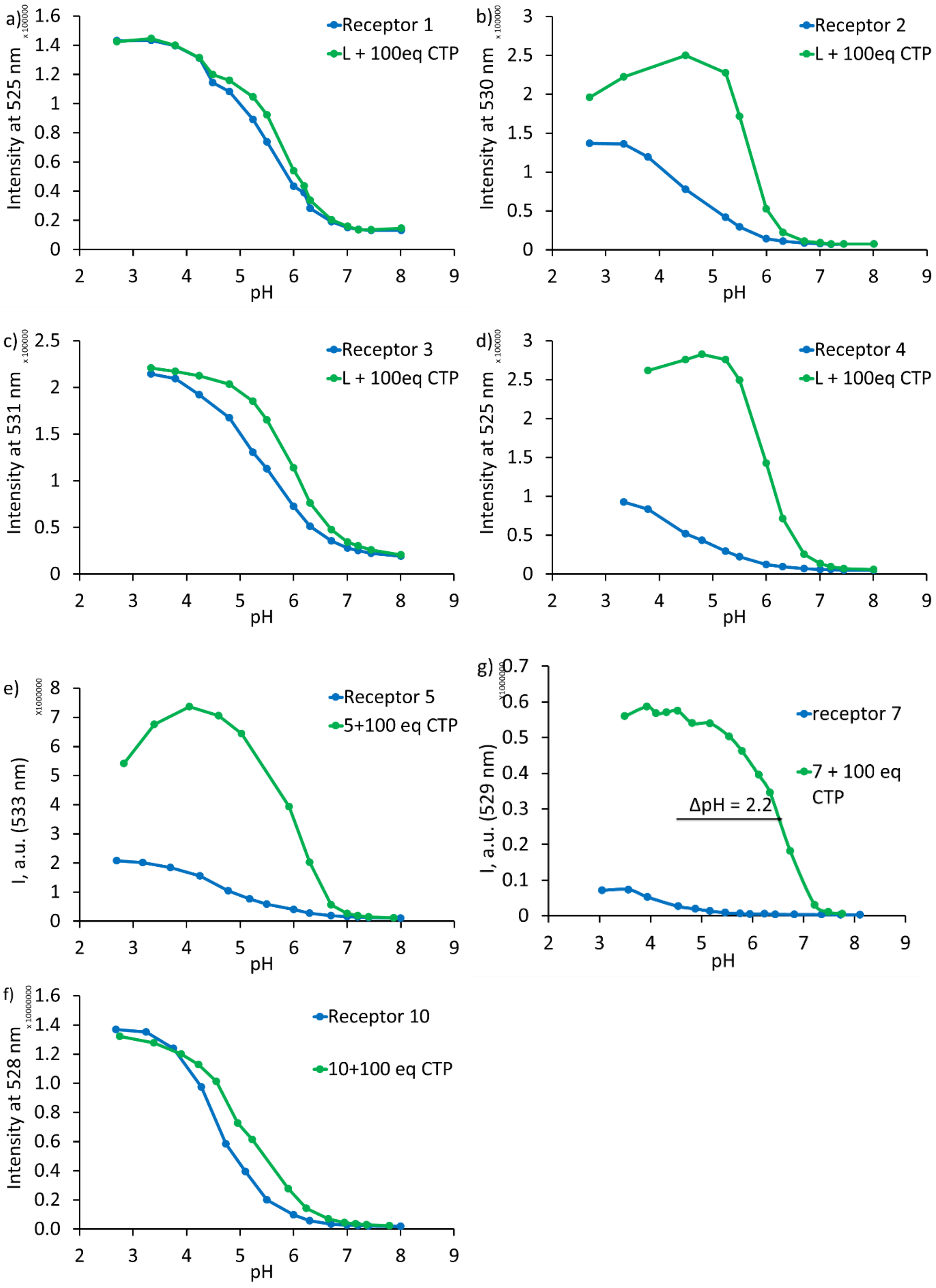

Figure S18. Relationship between fluorescence intensity of a receptor and pH in the absence and in the presence of CTP; a)-f) correspond to the receptors **1-5 and 7** and reference receptor **10**, respectively. Solutions with fixed pH values were prepared from 50 mM MOPSO adjusted with NaOH or HCl to the desired pH.

Potentiometric titrations

All solutions for potentiometric titrations were prepared as 0.05 M NaCl solution with 0.5 mM concentration of receptors in deionised water. For titrations standard 0.1M NaOH solution was used. The potentiometric titrations were carried out on a Mettler Toledo G20 Titrator equipped with a DGi 102-Mini pH-electrode. The electrode was calibrated with standard calibrating solutions from Mettler Toledo. The reaction vessel was kept at constant temperature 23°C. The value of K'w was determined from the data obtained in the alkaline range of the titration, and found to be equal to 13.88 in our experimental conditions. The titration experiment was carried out as follows: in the reaction vessel was placed a solution of a compound (and calculated amount of HCl$_{)}$; after stirring the solution for 5 minutes the titrations was started. The experiment was repeated 3-5 times. For the experiment in the presence of an anion, the corresponding amount of its solution was added prior to the titrations. The obtained data was imported to the HYPEQUAD 2008 program and fitted to obtained protonation constants.

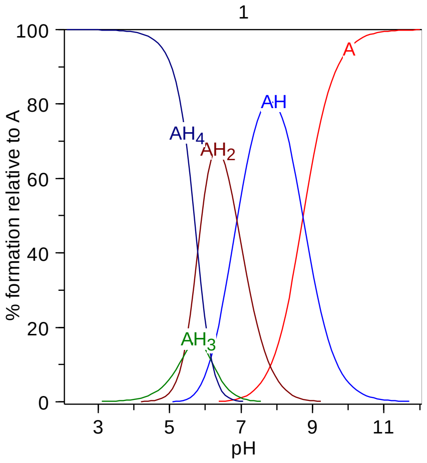

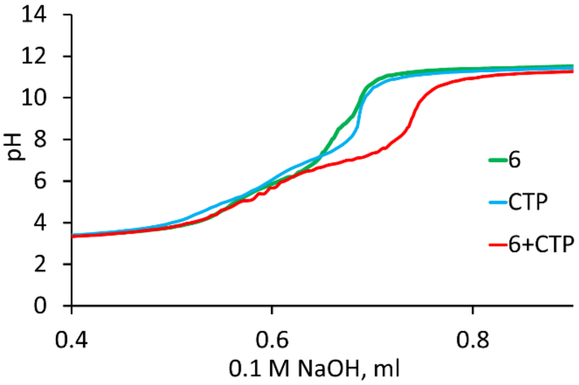

Protonation constants for receptor **6:**

Complex	log*K* *stepwise (standard deviation)*
**6**H^+	8.74(2)
**6**H_2^{2+}	6.89(1)
**6**H_3^{3+}	5.39(3)
**6**H_4^{4+}	4.98(3)

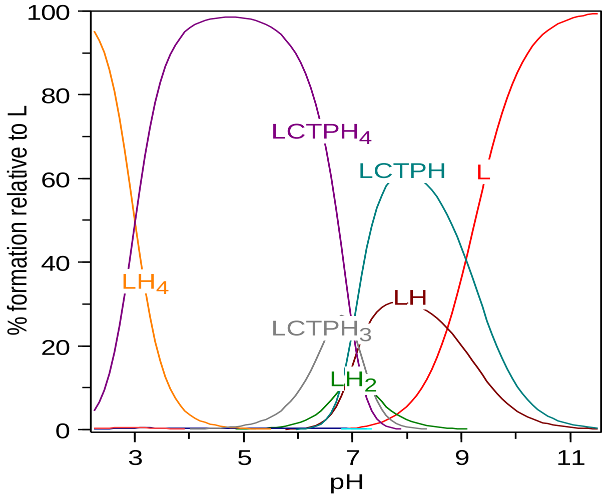

Protonation constants for complex formation **6** with CTP (log*β*):

Complex	Logβ
**6**$(CTP^{4-})H^{+}$	12.02(3)
**6**$(CTP^{4-})H_2^{2+}$	16.69(4)
**6**$(CTP^{4-})H_3^{3+}$	26.03(3)
**6**$(CTP^{4-})H_4^{4+}$	32.98(4)
**6**$(CTP^{4-})H_5^{5+}$	34.04(5)

Figure S19. Species distribution obtained from potentiometric titration of receptor **6**. Potentiometric titration of receptor **6**, CTP and their mixture (right). Species distribution for titration of **6** (0.0032 M)+CTP (0.00975 M).

Fluorescence and UV-Vis titrations

The resulting data was imported in HypSpec program[1] and the data was fitted to obtain stability constants.

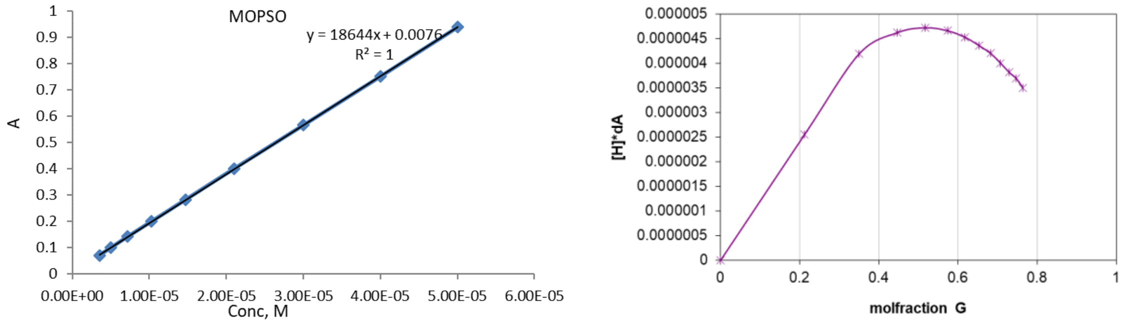

Figure S20. Absorption vs. concentration of receptor **6**. The observed linear relationship prove the absence of aggregation under the chosen conditions 50 mM MOPSO buffer (5% DMSO, pH 6.2). Job's plot obtained according to UV-Vis measurements of **6** and CTP in different ratios.

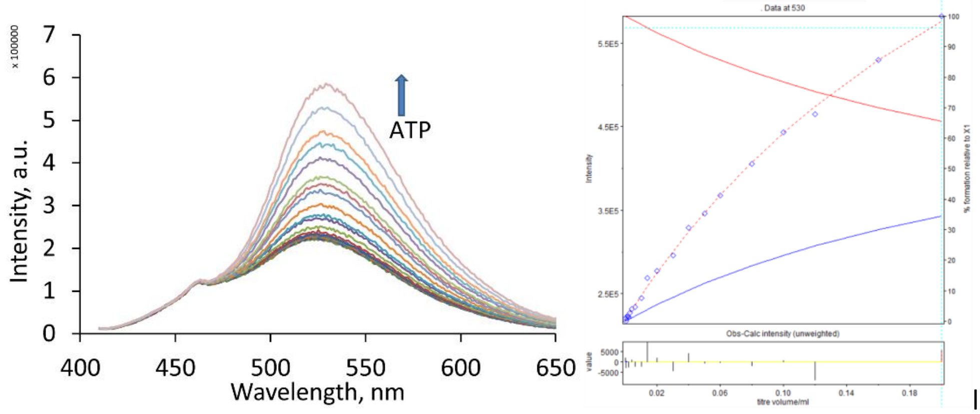

log*K* = 2.76±0.01

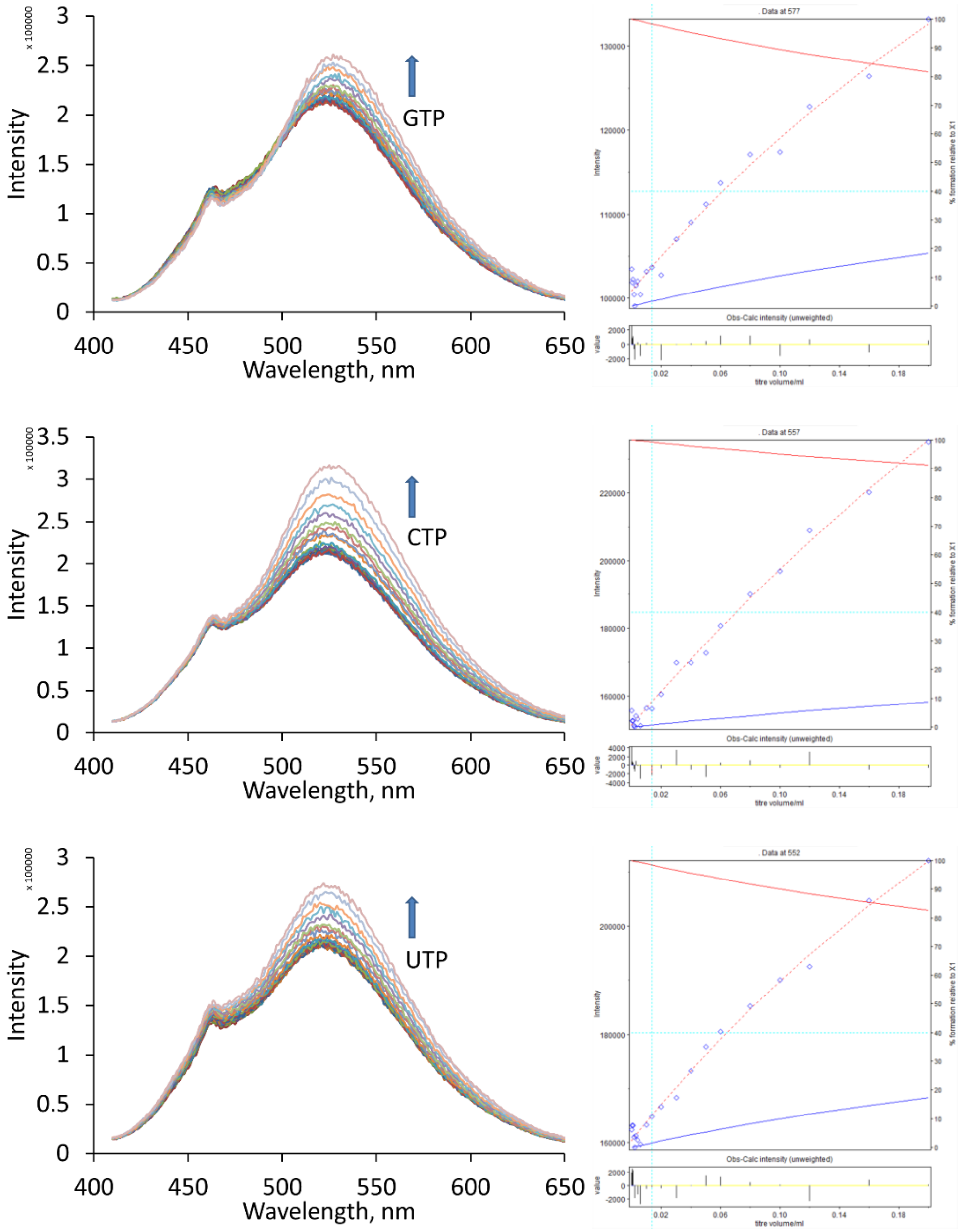

log*K* = 2.39±0.01

log*K* = 2.02±0.01

log*K* = 2.36±0.02

Figure S21. Fluorescence titrations together with the fitting curves (shown as red dotted line) obtained for receptor **1**. Conditions: 50 mM MOPSO buffer (5% DMSO, pH 6.2).

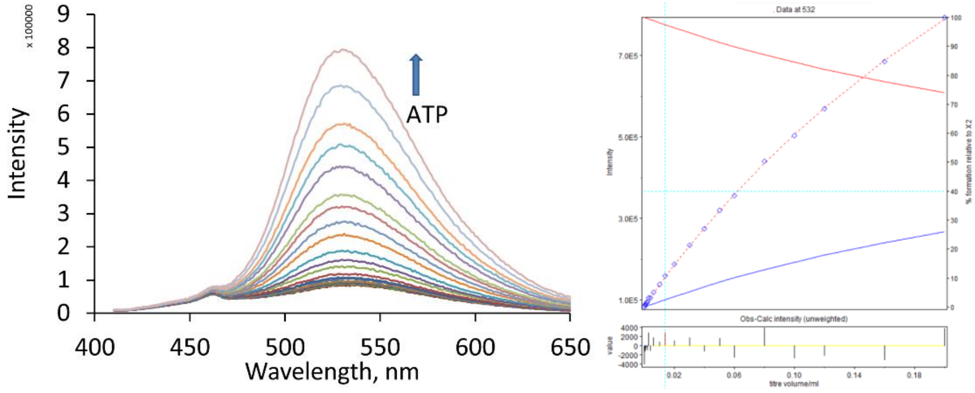

log*K* = 2.58±0.01

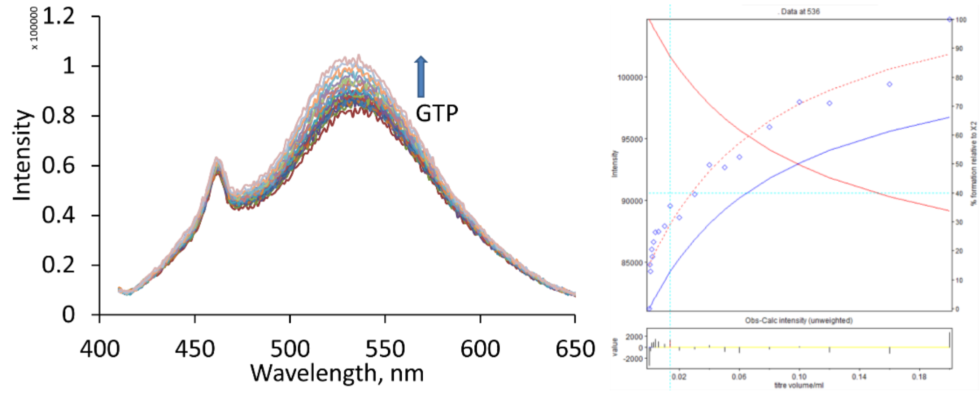

logK = 3.34±0.02

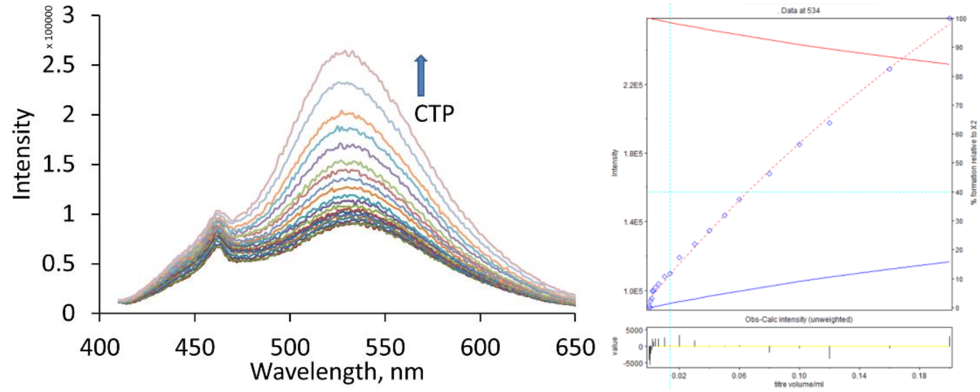

logK = 2.31±0.01

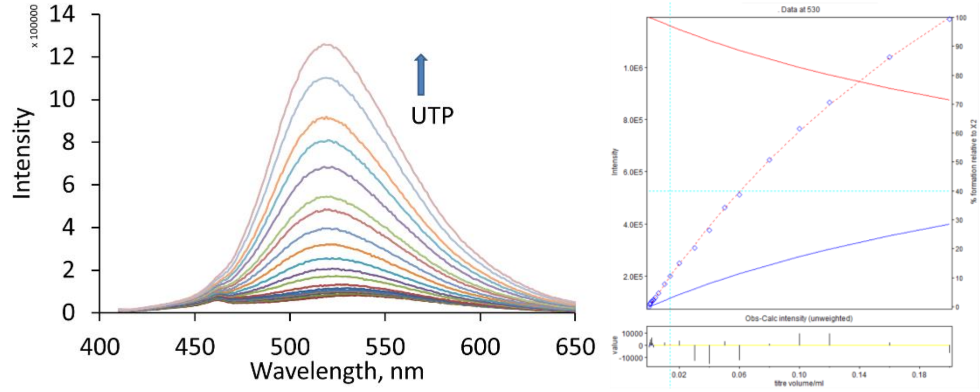

logK = 2.64±0.01

Figure S22. Fluorescence titrations together with the fitting curves (shown as red dotted line) obtained for receptor **2**. Conditions: 50 mM MOPSO buffer (5% DMSO, pH 6.2)

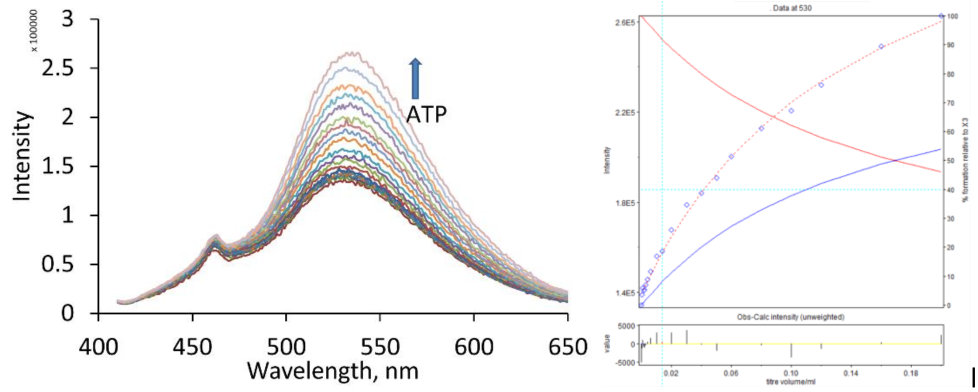

logK = 3.31±0.01

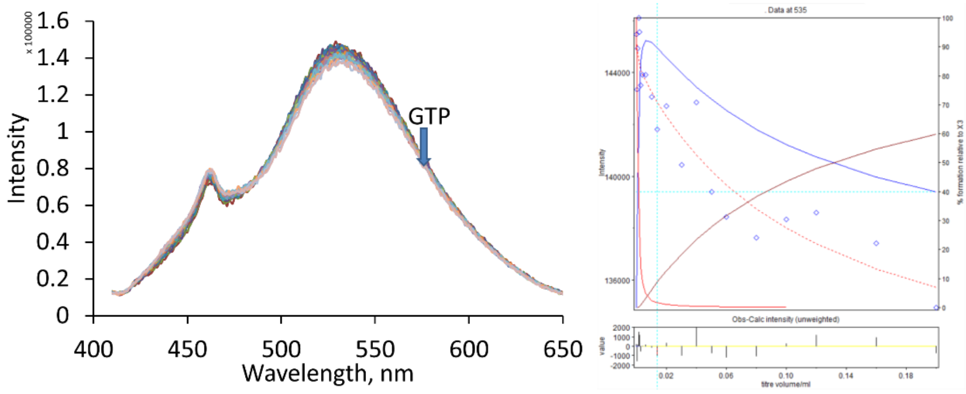

exceeding error

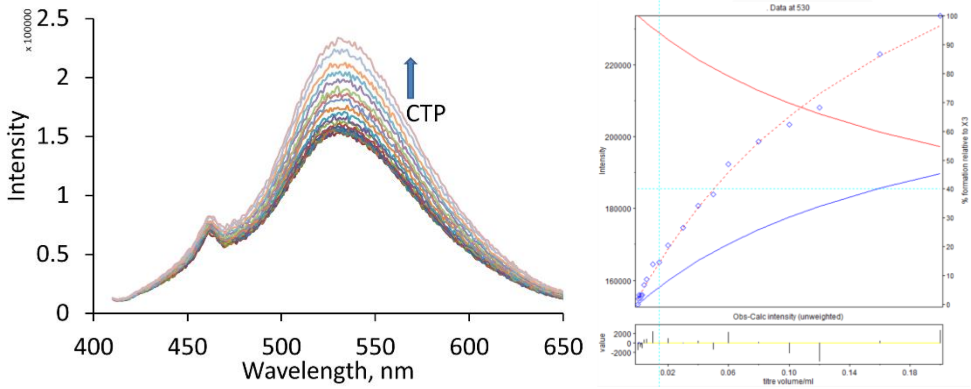

log*K* = 2.96±0.01

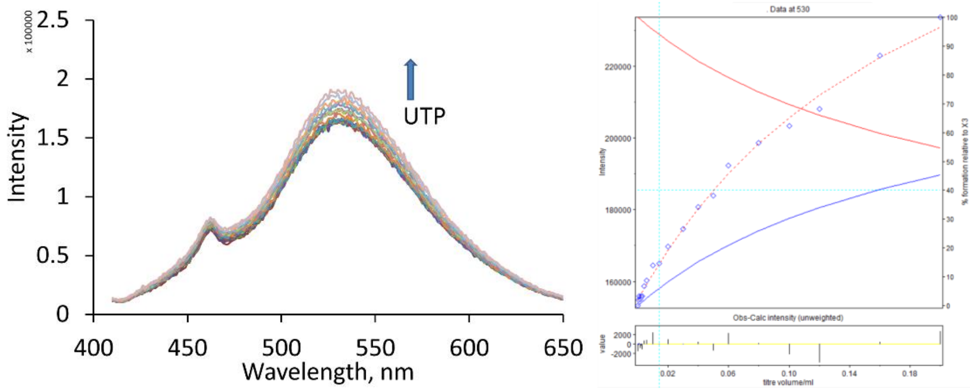

log*K* = 2.88±0.01

Figure S23. Fluorescence titrations together with the fitting curves (shown as red dotted line) obtained for receptor **3**. Conditions: 50 mM MOPSO buffer (5% DMSO, pH 6.2).

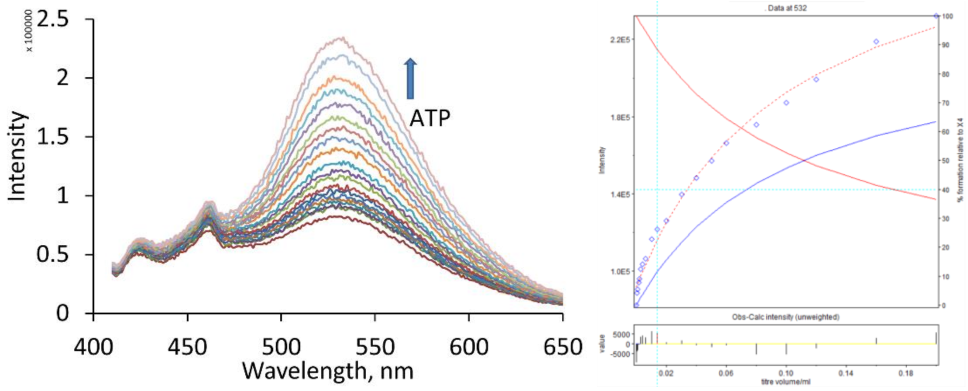

log*K* = 3.28±0.01

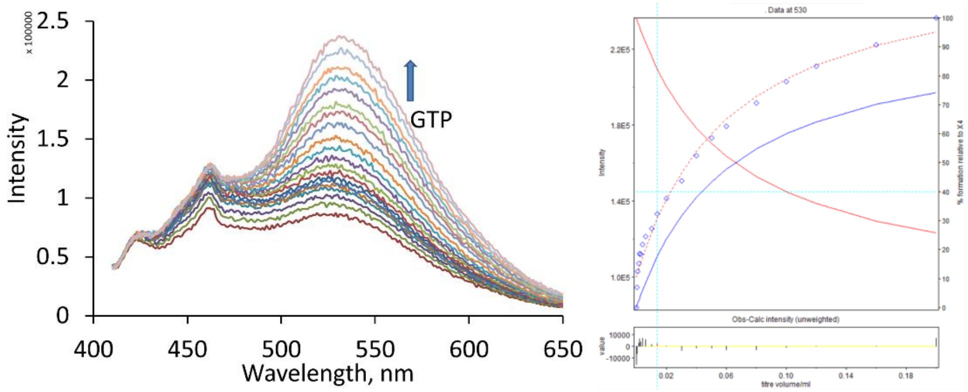

logK = 3.50±0.03

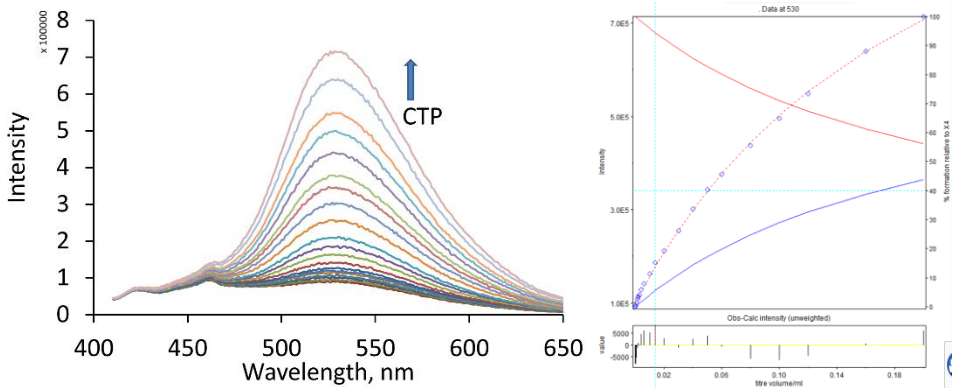

logK = 2.93±0.01

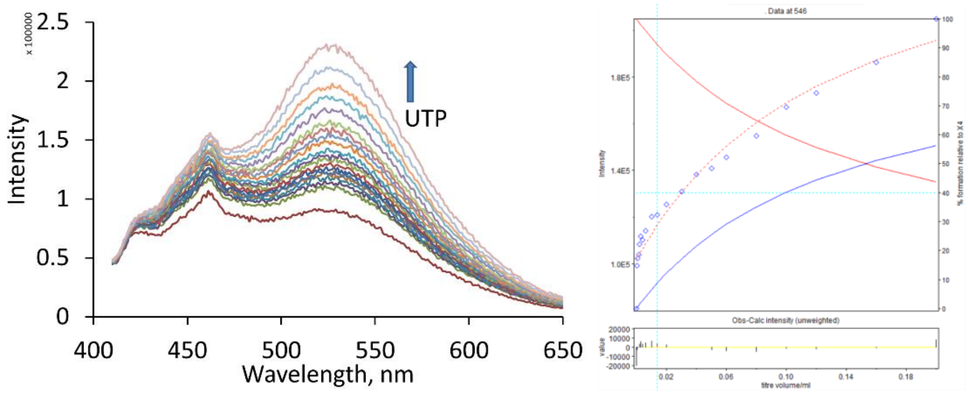

logK = 3.15±0.02

Figure S24. Fluorescence titrations together with the fitting curves (shown as red dotted line) obtained for receptor **4**. Conditions: 50 mM MOPSO buffer (5% DMSO, pH 6.2)

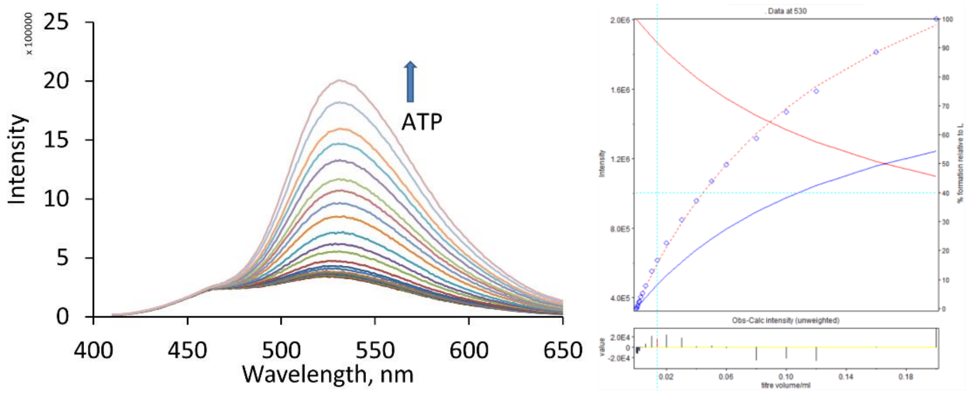

logK = 3.33±0.01

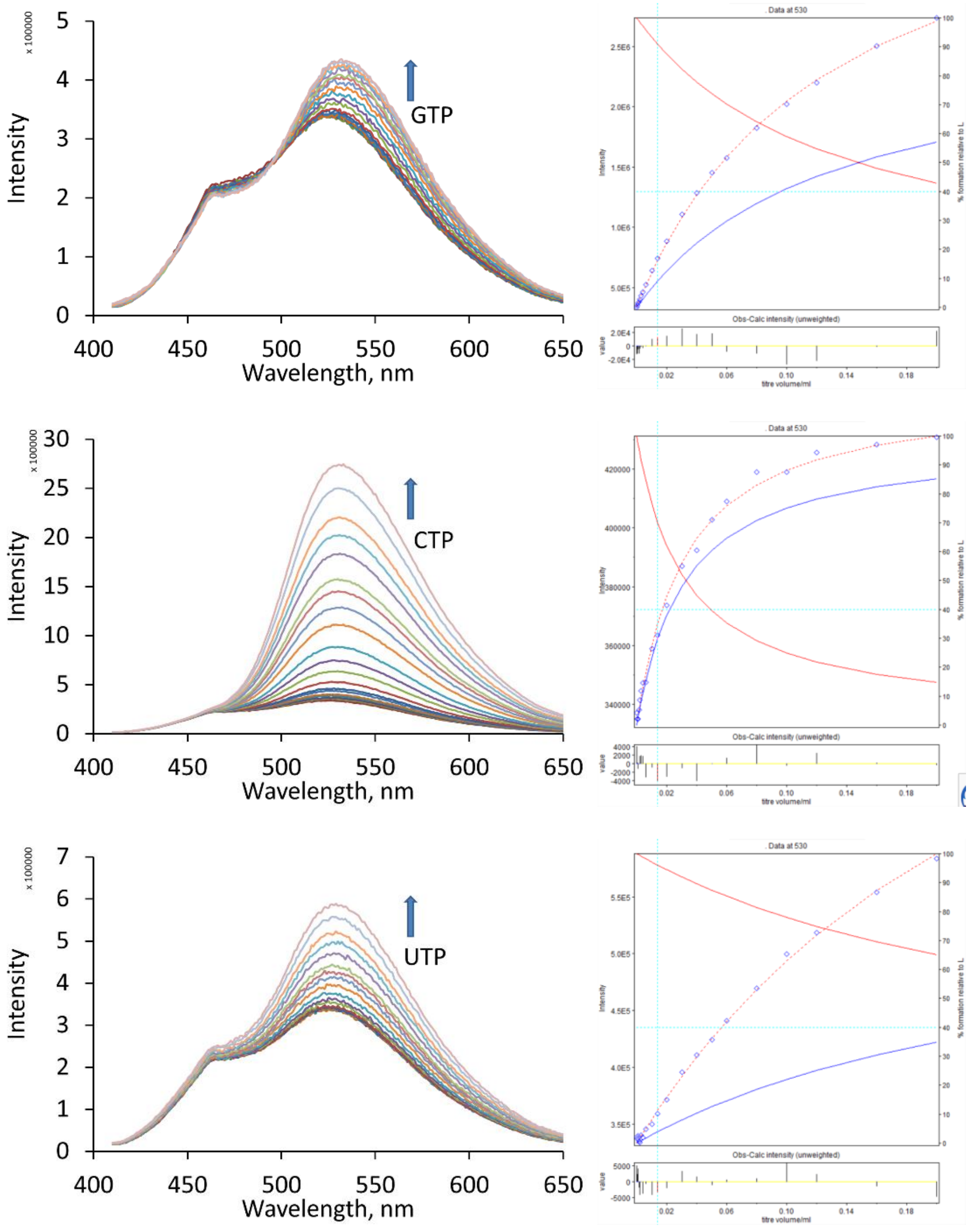

logK = 3.43±0.01

logK = 3.78±0.01

logK = 2.27±0.01

Figure S25. Fluorescence titrations together with the fitting curves (shown as red dotted line) obtained for receptor **5**. Conditions : 50 mM MOPSO buffer (5% DMSO, pH 6.2).

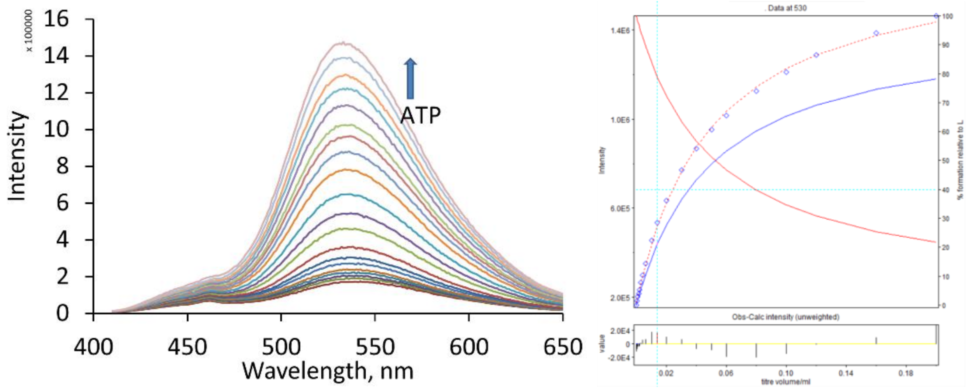

6 with ATP at pH 6.2, log*K* = 3.36±0.01

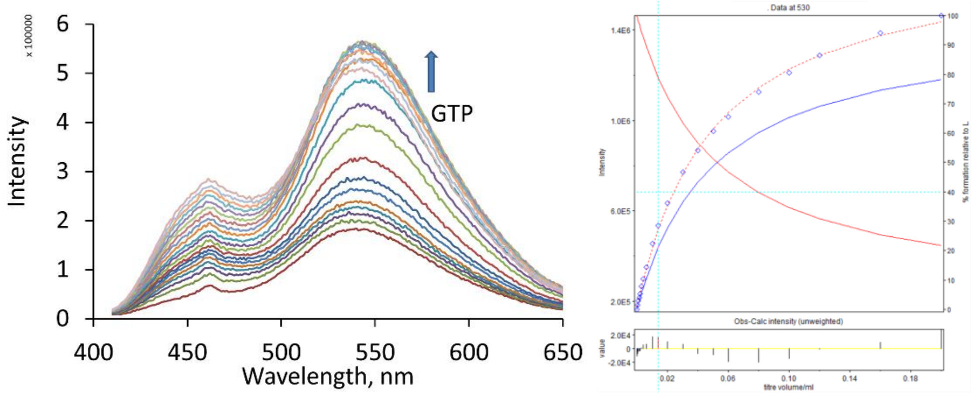

6 with GTP, pH 6.2, log*K* = 3.40±0.01

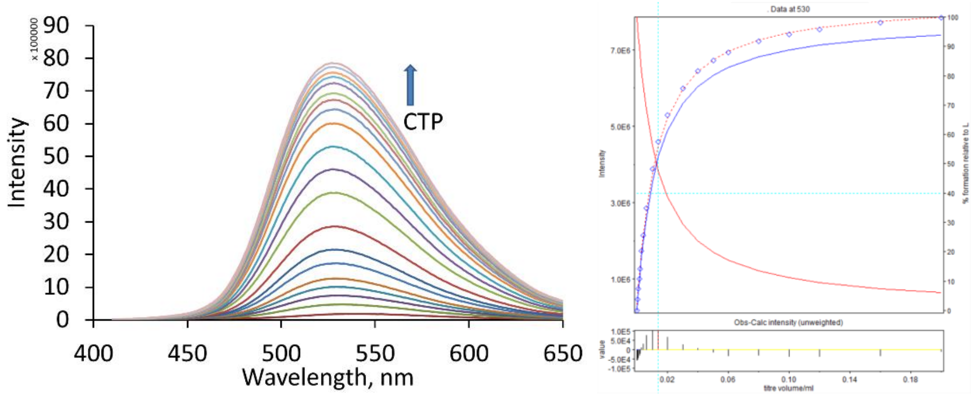

6 with CTP, pH 6.2, log*K* = 4.43±0.01

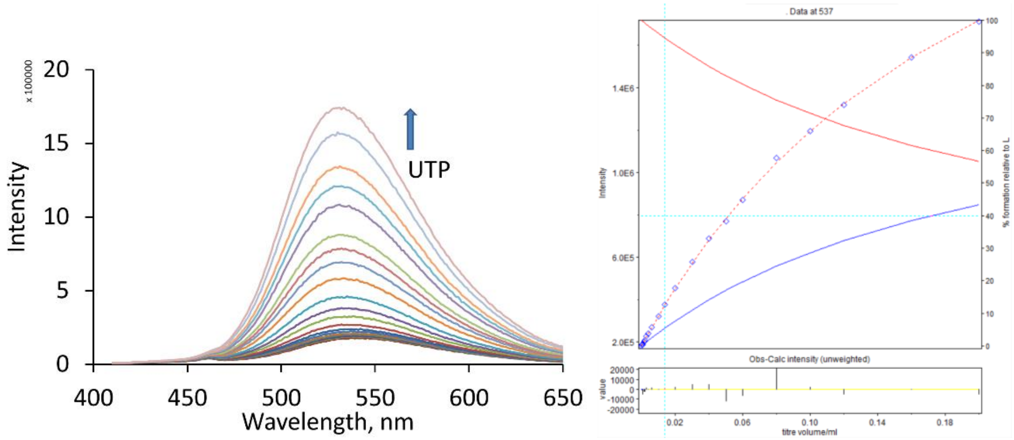

6 with UTP, pH 6.2, log*K* = 3.20±0.01

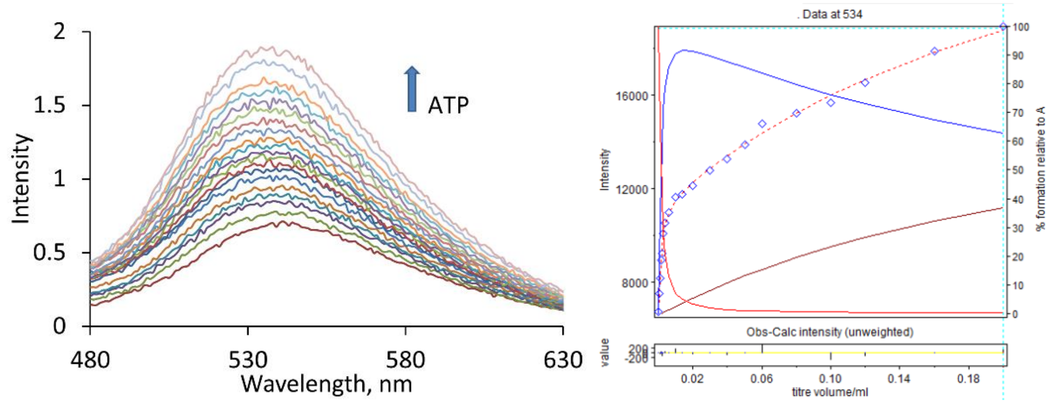

6 with ATP, pH 7.4, logK_{11} = 5.50±0.01, logK_{12} = 2.80±0.01

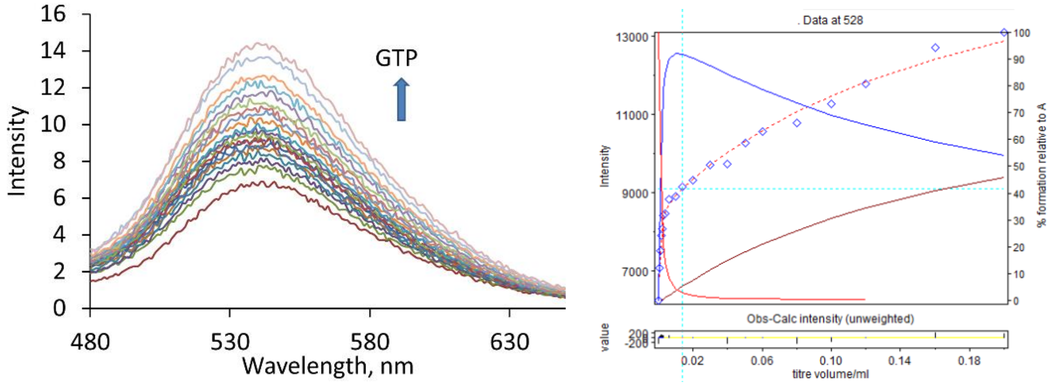

6 with GTP, pH 7.4, logK_{11} = 5.72±0.01, logK_{12} = 2.97±0.01

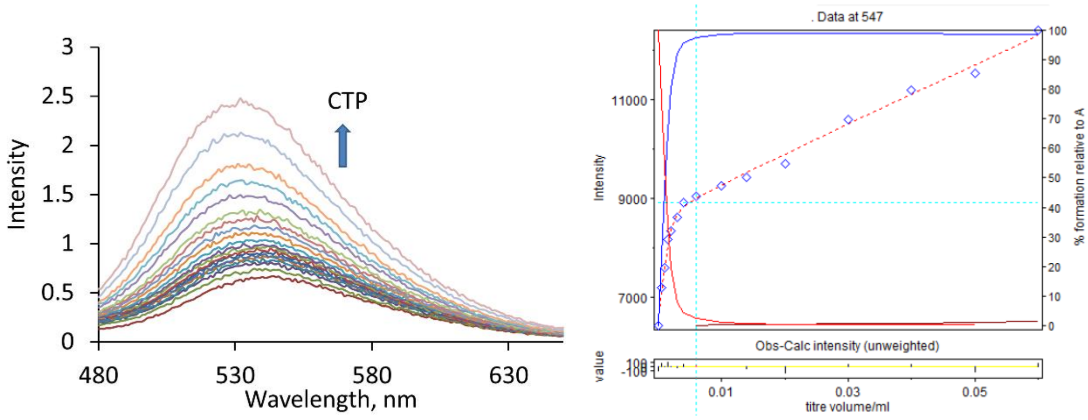

6 with CTP, pH 7.4, logK_{11} = 6.32±0.01, logK_{12} = 2.78±0.01

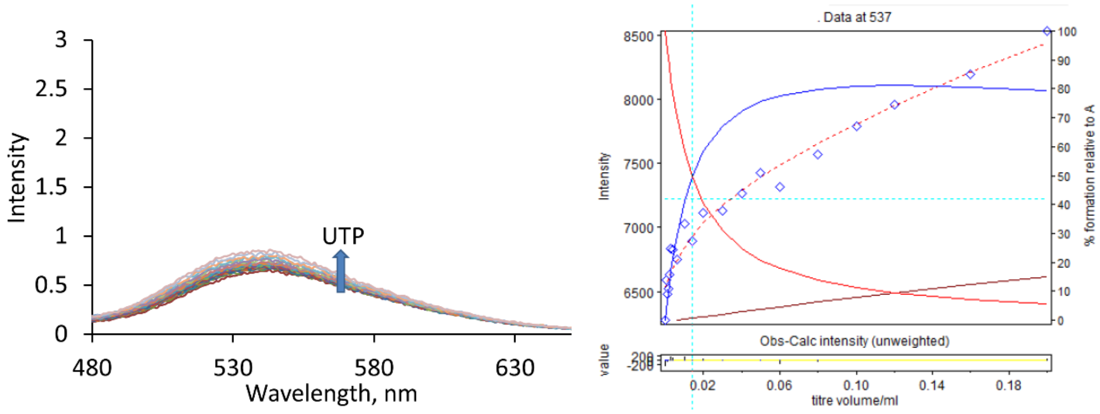

6 with UTP, pH 7.4, logK_{11} = 4.18±0.01, logK_{12} = 2.32±0.01

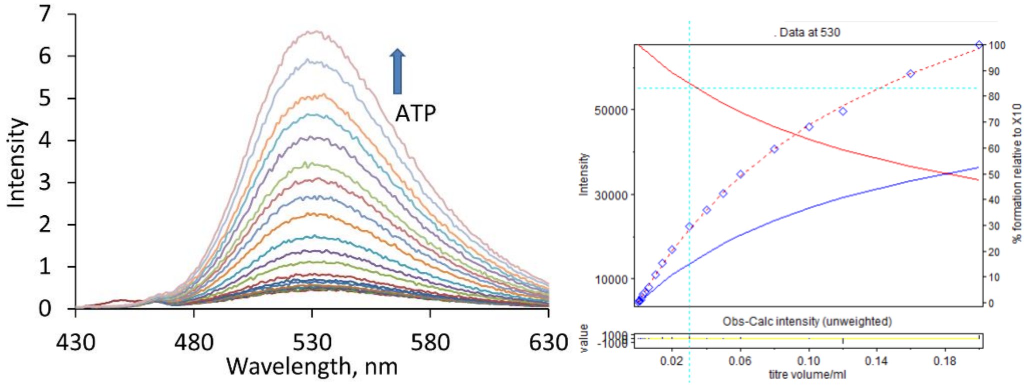

7 with ATP, pH 6.2, log$K$ = 3.08±0.01

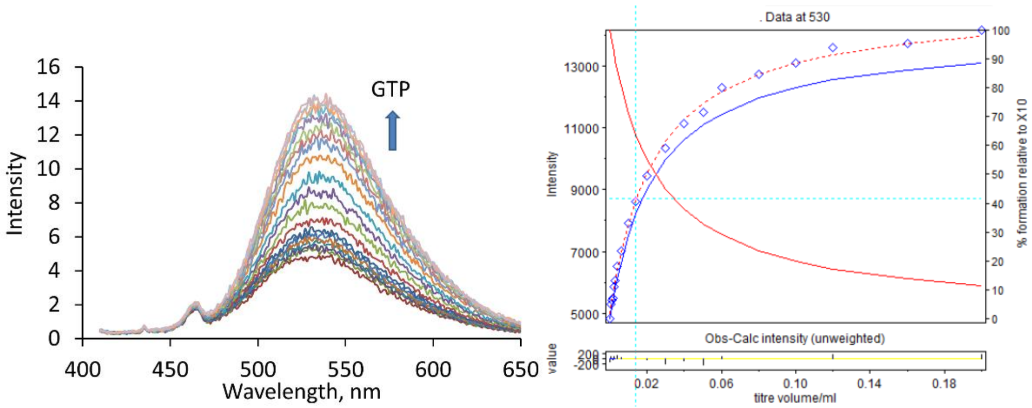

7 with GTP, pH 6.2, log$K$ = 3.93±0.01

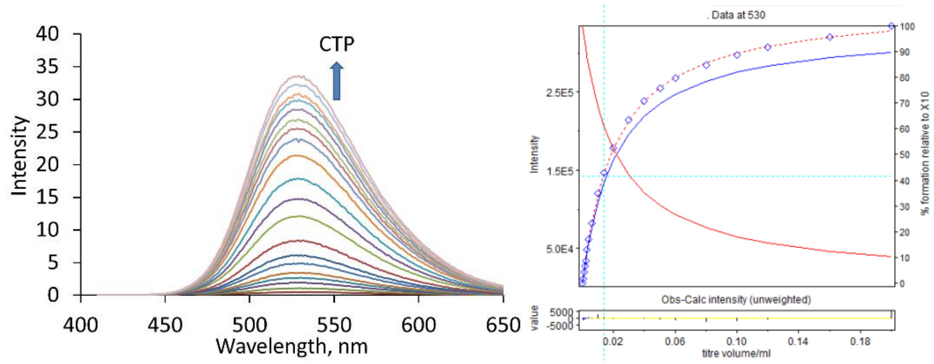

7 with CTP, pH 6.2, log*K* = 3.98±0.01

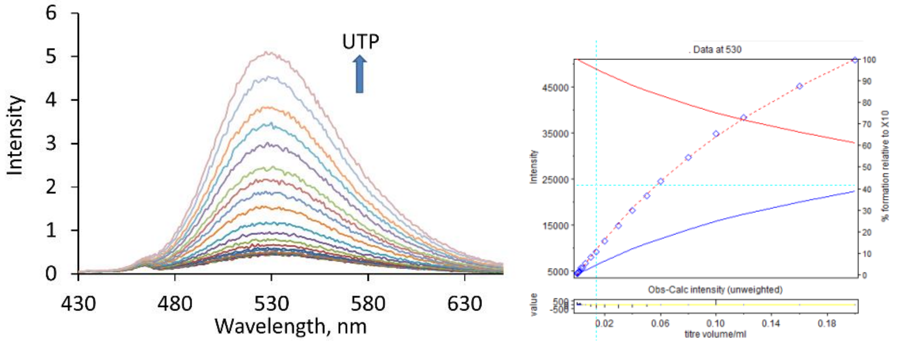

7 with UTP, pH 6.2, log*K* = 2.84±0.02

Figure S26. Fluorescence titrations together with the fitting curves (shown as red dotted line) obtained for receptors **6** and **7**. Conditions: 50 mM MOPSO buffer (5% DMSO), pH 6.2 or 7.4.

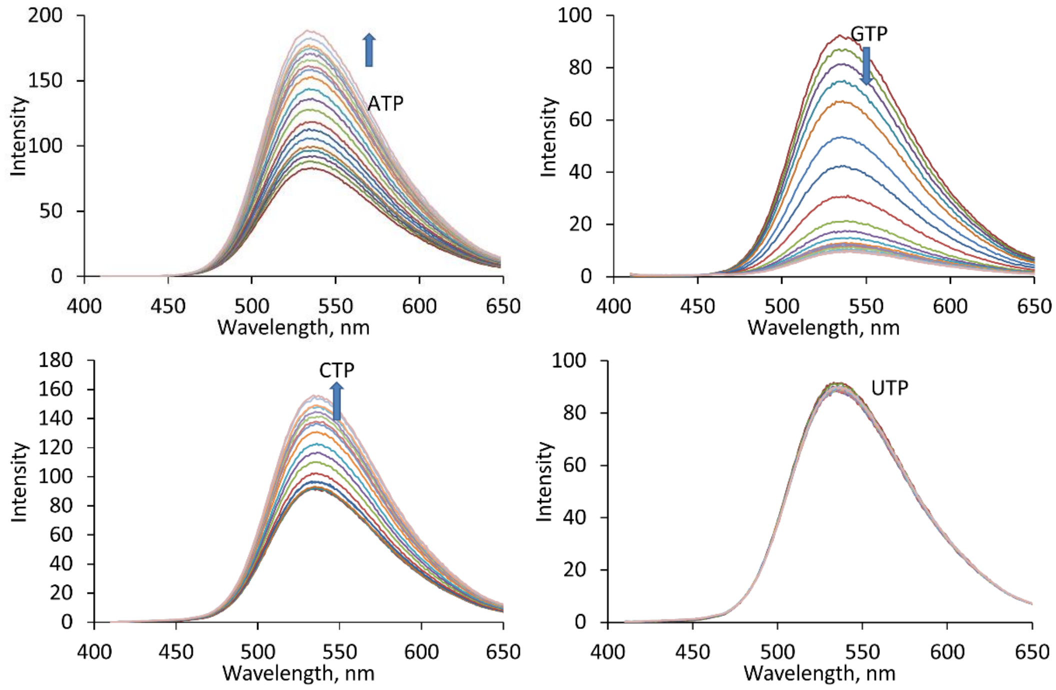

Figure S27. Fluorescence titration of reference compound **10** with nucleotides.

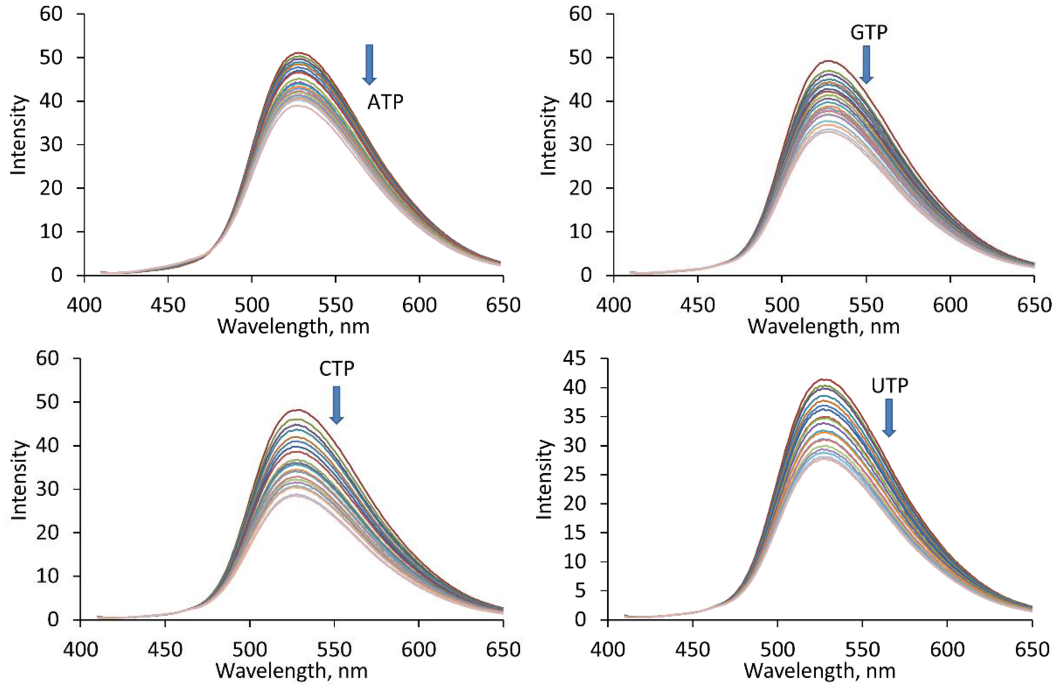

Figure S28. Fluorescence titration of reference compound **11**.

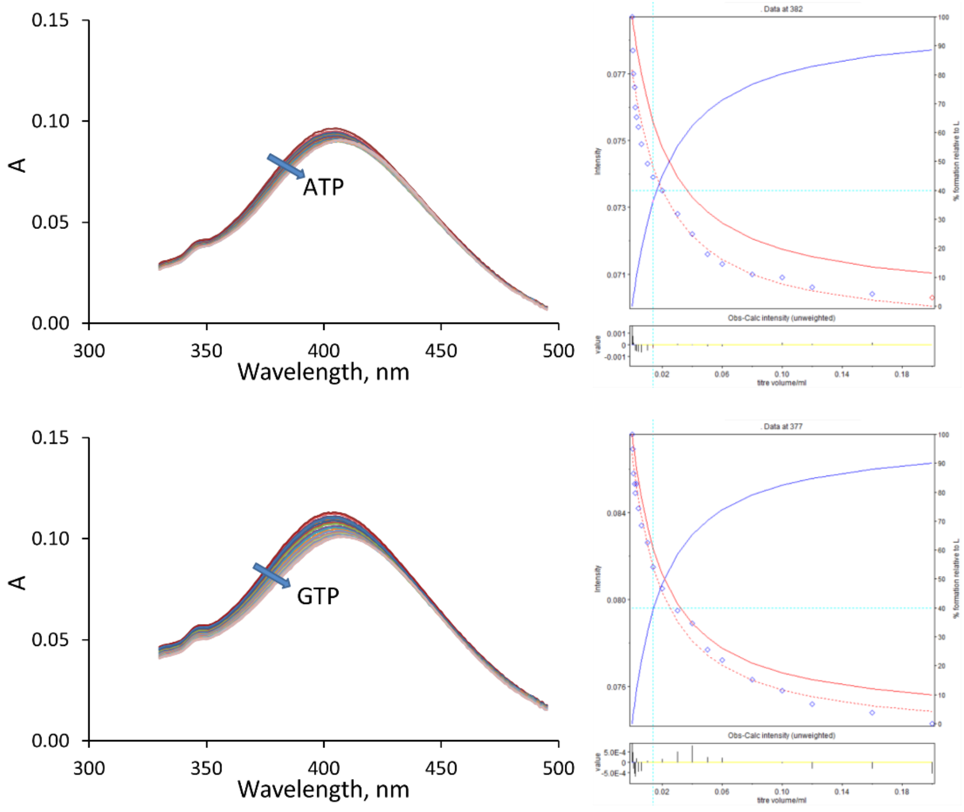

log*K* = 3.64±0.01

log*K* = 3.62±0.02

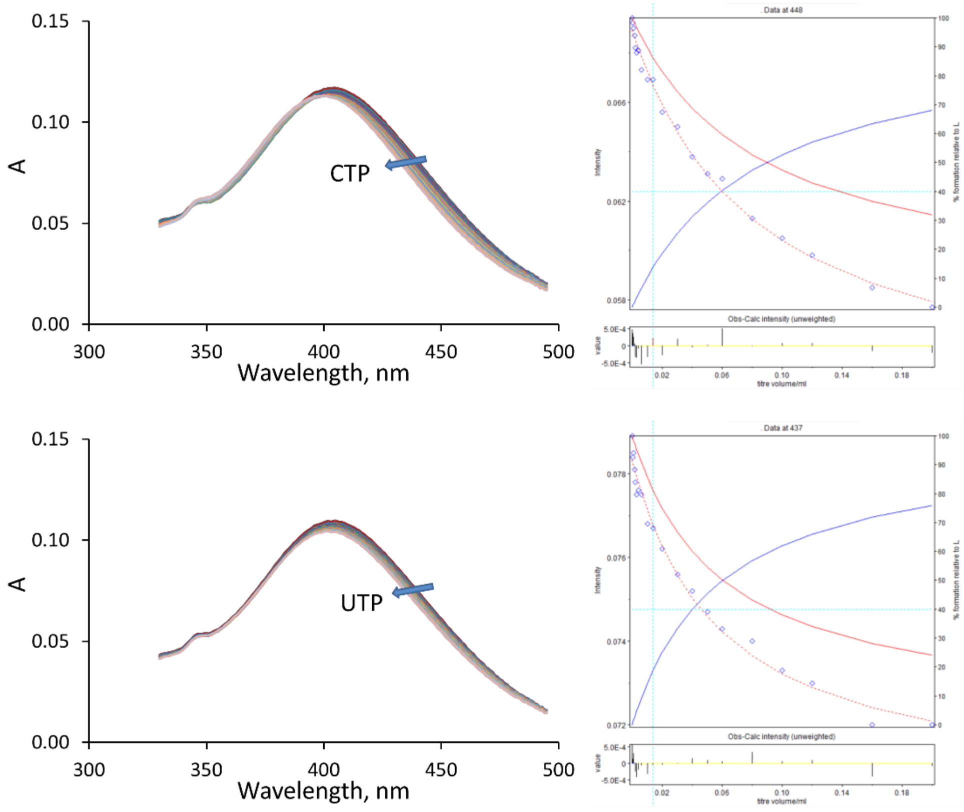

logK = 3.80±0.01

logK = 3.20±0.01

Figure S29. UV-Vis titrations together with the fitting curves (shown as red dotted line) obtained for receptor **5**.

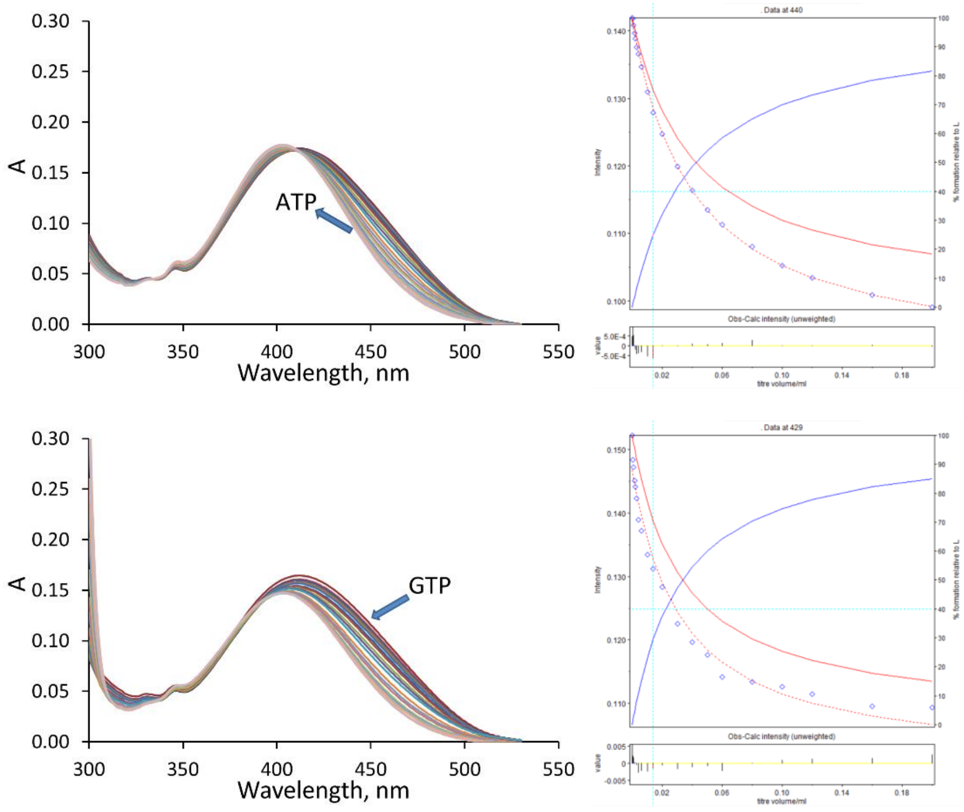

logK = 3.38±0.01

logK = 3.54±0.04

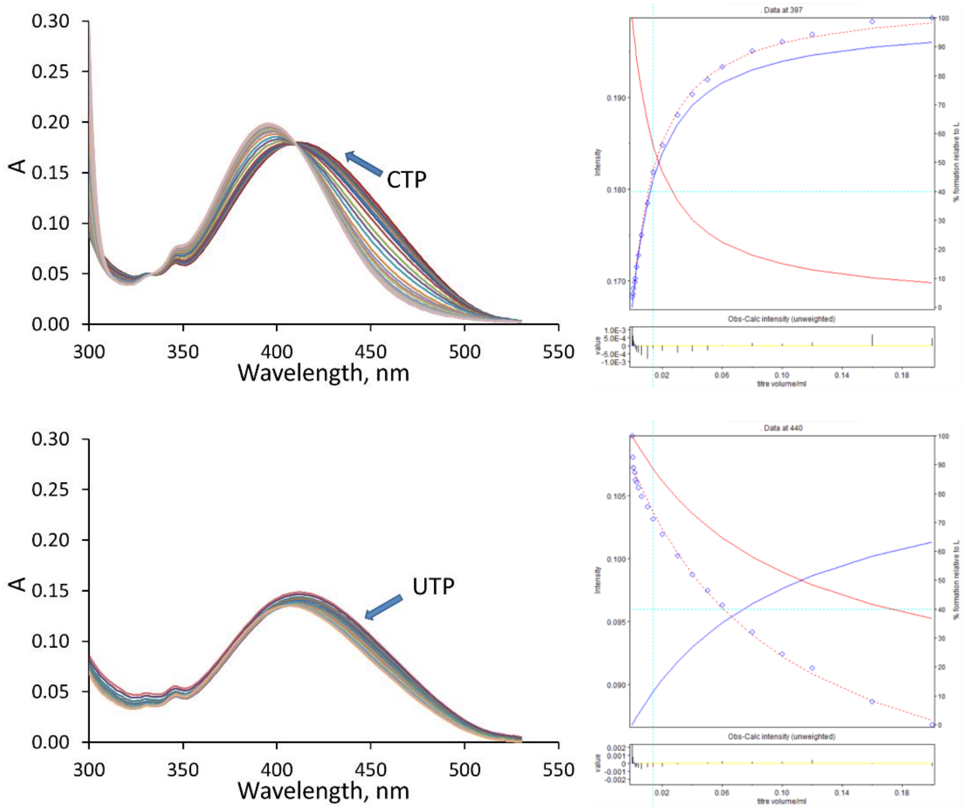

logK = 4.38±0.01

logK = 3.27±0.01

Figure S30. UV-Vis titrations together with the fitting curves (shown as red dotted line) obtained for receptor **6**.

Competition experiment

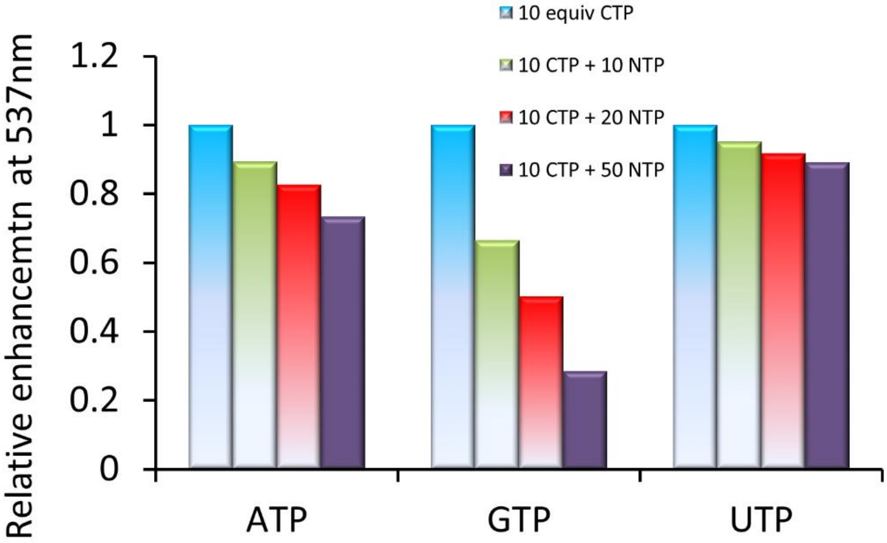

Figure S31. Competition experiment shows relative fluorescence enhancement for addition of 10 equiv of CTP to receptor **6** in the absence of a competing nucleotide (blue bar), and in the presence of a competing nucleotide: 10 equiv (green line), 20 equiv (red line) and 50 equiv (violet line).

The limit of detection (LOD) and limit of quantitation (LOQ)

The LOD and LOQ were calculated using the following formula: LOD = 3.3 σ/S. Where σ=the standard deviation of the analytical background response, and S = the slope of the calibration curve (plot of fluorescence intensity as a function of the nucleotide concentrations in the region of low analyte content).[2-4] To determine the standard deviation, the fluorescent intensity of **6** without any analyte was measured in 10 prepared probes (with concentration 1E-5M). Equation 1:

$$\sigma = \sqrt{\frac{1}{\mathrm{N}-1}\sum_{i=1}^{N}(m_i-\mu)^2}$$

where (m_1, m_2 , … , m_N) are the observed fluorescent intensities at certain wavelength values of the sample items, and μ is the mean value of these observations, while the denominator N stands for the number of probes. In our case, there were 10 samples, the standard deviation was 6716.16 with an average fluorescence intensity of 177385 a.u. at 530 nm.

There was a good linearity in micromolar concentration levels between fluorescent intensity data at 530 nm and concentrations of ATP. The linear equation (equation 2)

$$y = Sx + b$$

was found to be y= 9.00E+10x+ 291663.0234 **(Figure S32)**, where y is the fluorescent intensity data at 530 nm measured at given ATP concentration and x represents the concentration of ATP added. Thus, the detection limit and the limit of quantitation of **6** were calculated. LOD = 3.3σ/S= 3.3*6716.16/9.00E+10 =2.46E-07 M, LOQ = 3.3 σ/S=10*6716.16/9.00E+10= 7.46E-07 nM.

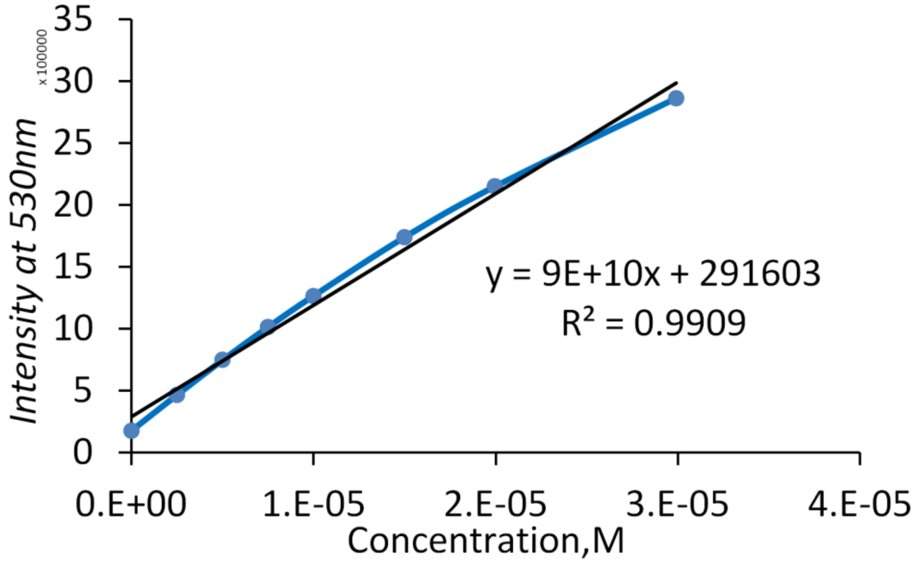

Figure S32. Fluorescence changes of receptor **6** at 530 nm with increasing amounts of CTP together with the corresponding trend line.

Femtosecond transient absorption spectroscopy

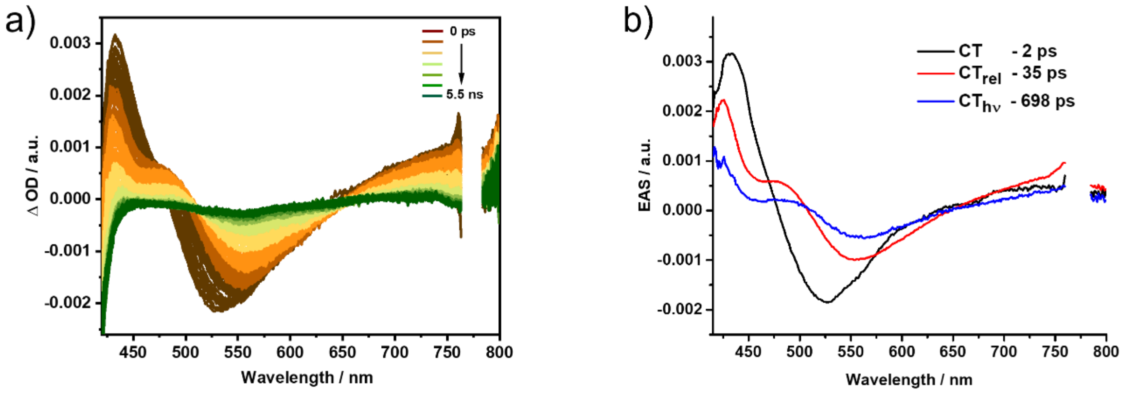

Figure S33. a) Femtosecond differential absorption spectra of receptor **6** + GTP (100 eq.), at time delays between 0 and 5.5 ns after 387 nm photo-excitation at room temperature. Measurements were done in 50 mM MOPSO buffer (5% DMSO, pH 6.2). b) Evolution associated spectra reconstructed from the sequential global analysis of femtosecond TA spectra of receptor **6** + GTP.

Electrospray ionization (ESI) studies

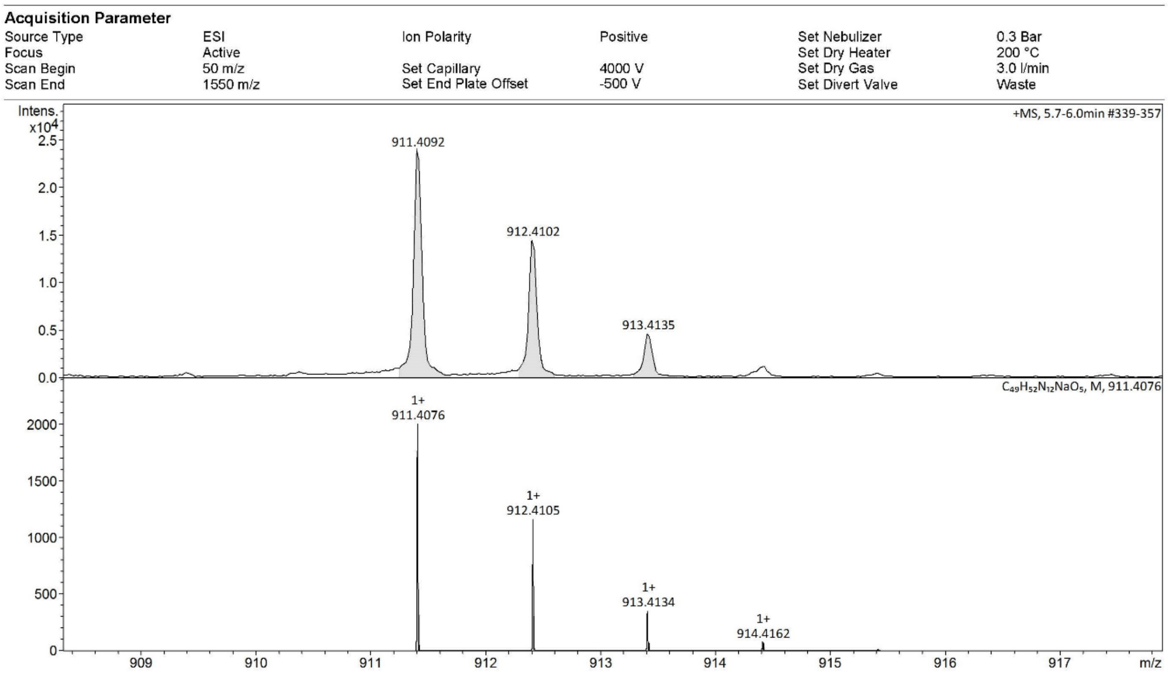

Figure S34. Observed and calculated (below) mass spectrum for [**6**·Cytosine·Na^+]$^+$ cation.

Acquisition Parameter

Source Type	ESI	Ion Polarity	Positive	Set Nebulizer	0.3 Bar
Focus	Not active			Set Dry Heater	200 °C
Scan Begin	300 m/z	Set Capillary	5000 V	Set Dry Gas	3.0 l/min
Scan End	3000 m/z	Set End Plate Offset	-500 V	Set Divert Valve	Waste

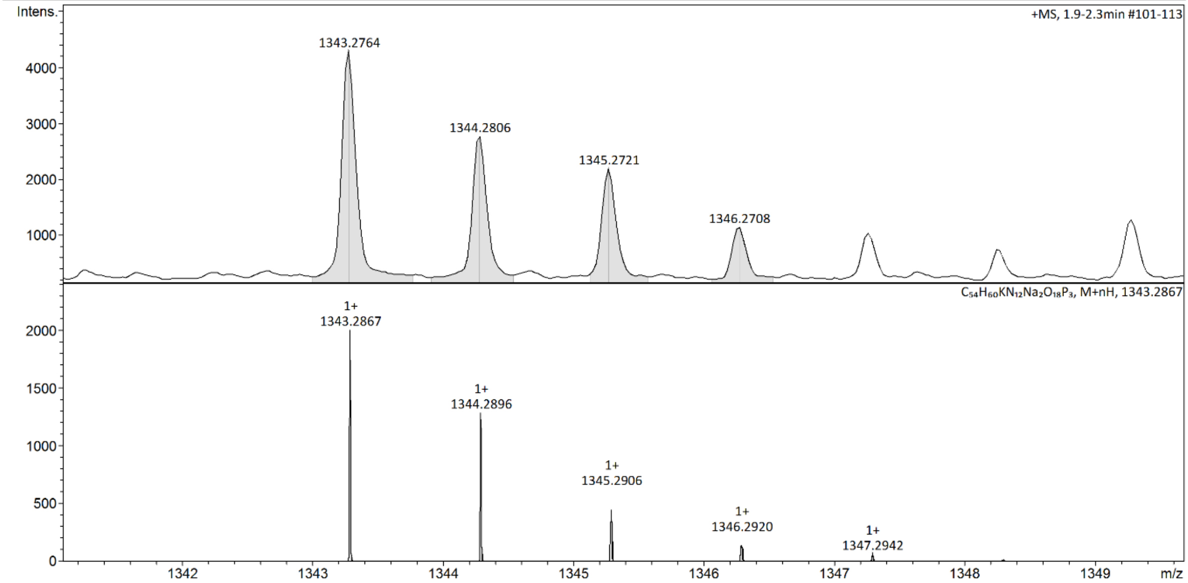

Figure S35. Observed and calculated (below) mass spectrum for for [**6**·CTP^{2-}·$Na_2^+K^+$]$^+$ cation.

^{1}H NMR studies

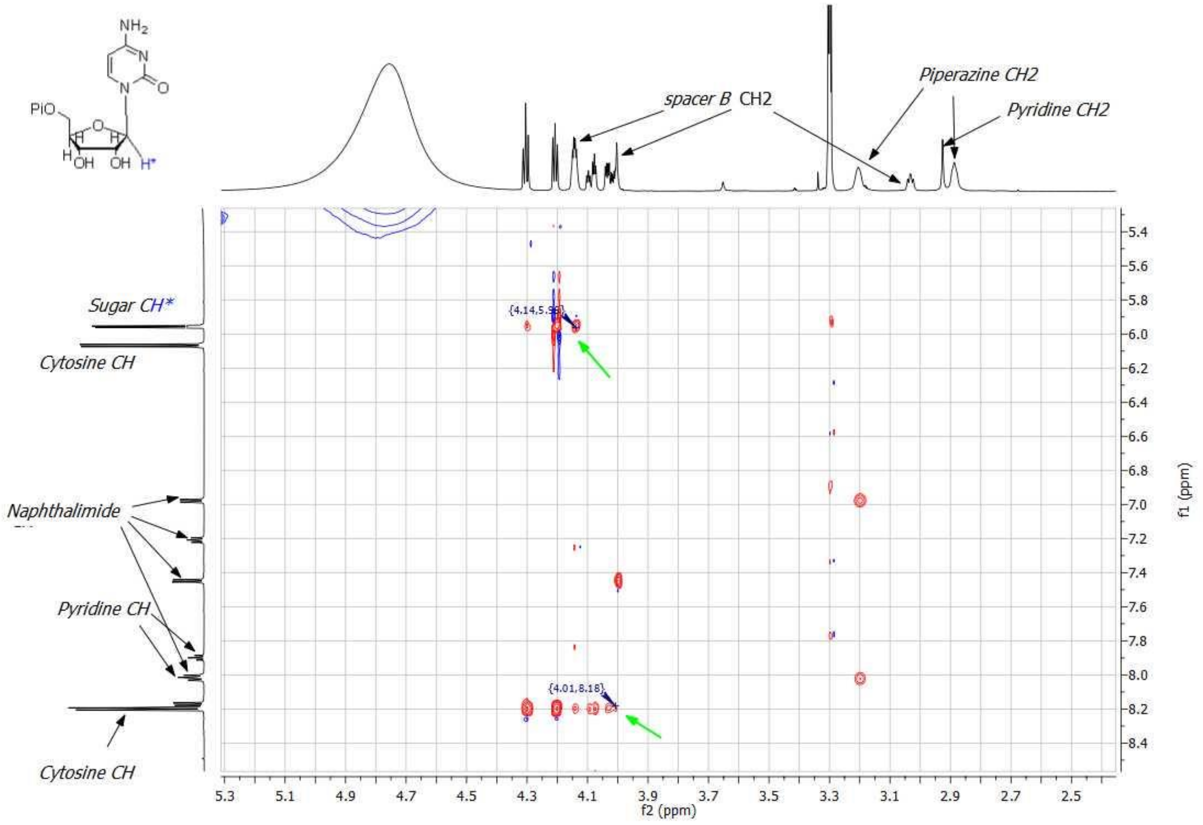

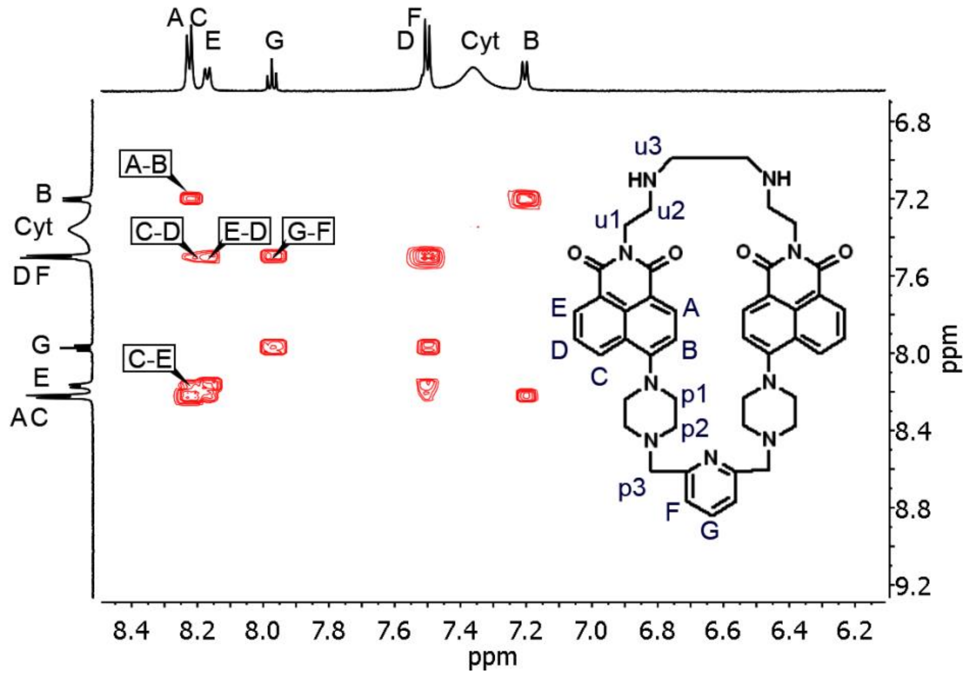

Figure S36. 2D ROESY and COSY spectra of the mixture of **6** with 3 equiv of CMP in CD_3OD-D_2O (1:1) solution.

NMR titrations were carried out by sequential addition of sodium salts to the NMR tube containing the receptors followed by the measurements. The 10% DMSO–buffer mixture was used because of solubility limitation of the receptors at 0.5 mM concentration. The following conditions were used: 0.5 mL of 0.5 mM solution of receptors in a 1:9 DMSO-d_6–D_2O (50 mM MOPSO buffer, pH 6.2) mixture.

Sodium salts dissolved in the same D_2O-based buffer (0.0625 M) were added as follows (equiv): 0.25; 0.5; 0.75; 1; 1.5; 2; 3; 5; 7; 10. The fitting was performed by HypNMR program.

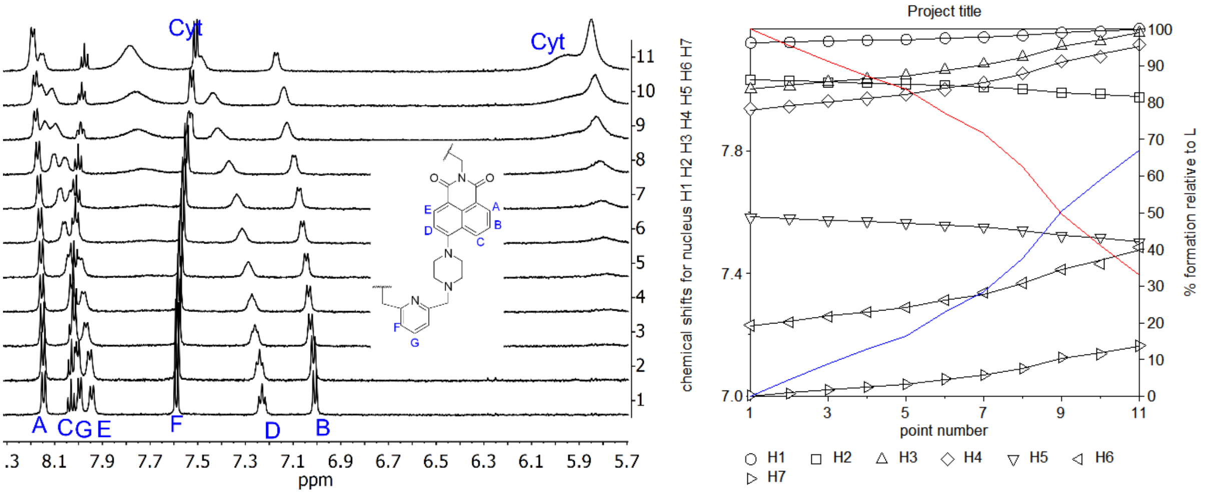

Figure S37. ^{1}H NMR titration of **6** (0.5 mM) with cytidine together with the fitting curves of the experimental data exported from HypNMR program are also shown. Conditions: 50 mM MOPSO buffer in D_2O (pH 6.2. 10% DMSO-*d6*).

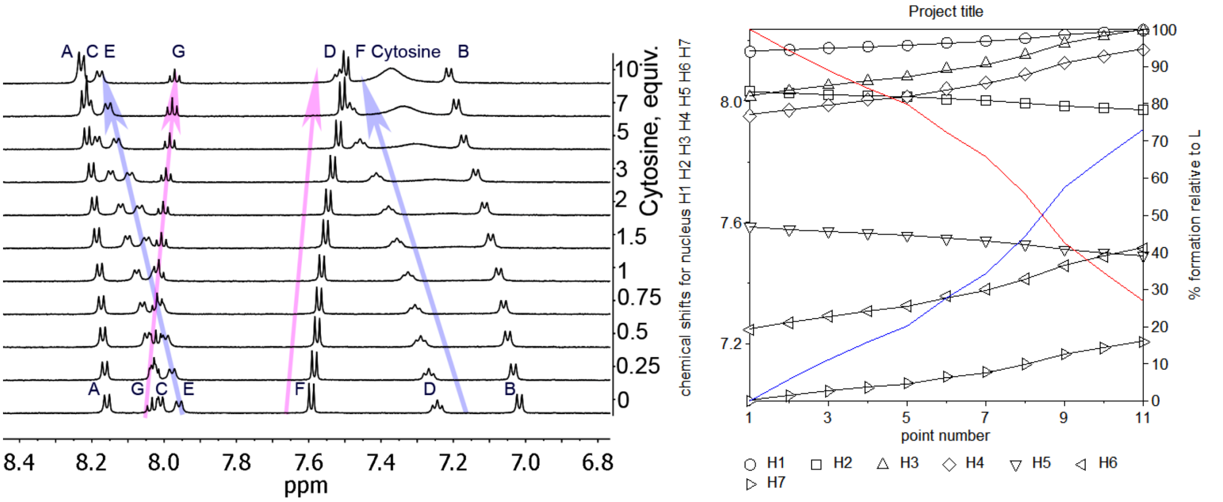

Figure S38. Annotated COSY NMR spectrum of **6** + 8 equiv of cytosine and ^{1}H NMR titration of **6** (0.5 mM) with cytosine. Fitting curves of the experimental data exported from HypNMR program are also shown. Conditions: 50 mM MOPSO buffer in D_2O (pH 6.2. 10% DMSO-*d6*).

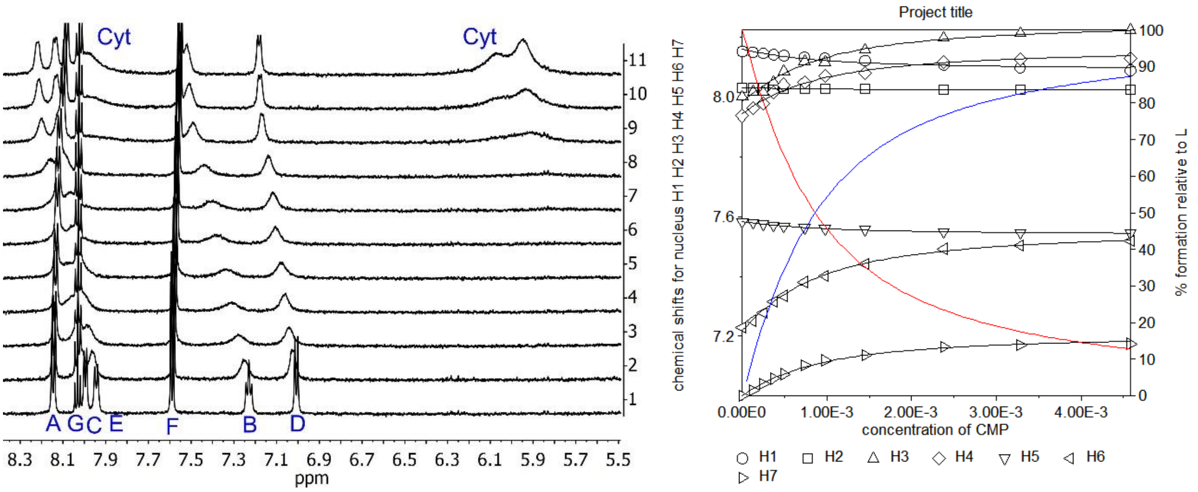

Figure S39. ^{1}H NMR titration of **6** (0.5 mM) with CMP together with the fitting curves of the experimental data exported from HypNMR program are also shown. Conditions: 50 mM MOPSO buffer in D_2O (pH 6.2. 10% DMSO-*d6*).

Studies with live cells

Uptake of 5 and 6

One day before the experiment, A2780 cells were seeded in RPMI 1640 medium containing 5% (v/v) FBS, 1% (v/v) L-Glu, and 1% (v/v) Pen/Strep (250 cells/µL, 0.1 mL/well) in a 96-well microtiter plate. On the day of the experiment, the cells were incubated with the indicated concentration of compounds **5** and **6** and 1% (v/v) DMSO for 2 h at 37 °C and 5% CO_2. The cells were washed twice with phosphate buffered saline (PBS, 10 mM, pH = 7.4), detached from the microtiter plate by using trypsin/ethylenediaminetetraacetic acid (EDTA) (0.25% trypsin, v/v, 50 µL), and resuspended in the cultivation medium (5% FBS, 100 µL). Finally, the fluorescence of all viable cells from a total cell count of 5000 was determined by using flow cytometry (FITC: λ_{ex} = 488 nm, λ_{em} = 525/40 nm; KO525: λ_{ex} = 405 nm, λ_{em} = 525/40 nm). Three independent experiments were performed and the standard deviation (SD) was calculated. An unpaired Student's t-test was performed for statistical analysis (*: p< 0.05; **: p< 0.01; ***; p< 0.001; ns: p≥ 0.05).

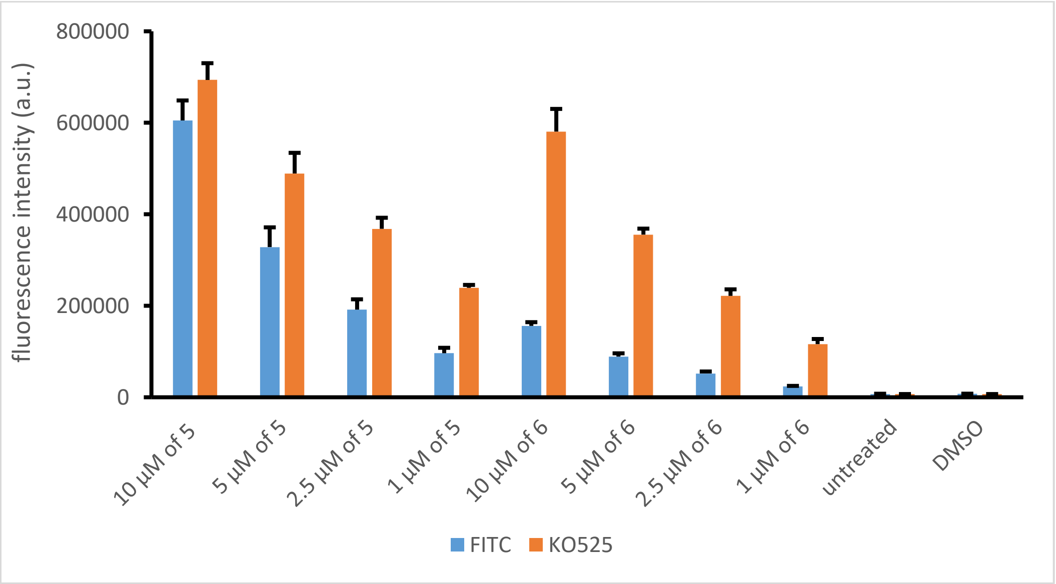

Figure S40. Uptake of **5** and **6** in A2780 cells by quantification of fluorescence intensity in viable cells. A2780 cells were incubated with the indicated concentration of **5** or **6** or just the carrier DMSO for 2 h before the fluorescence per single cell was determined (FITC: λ_{ex} = 488 nm, λ_{em} = 525/40 nm; KO525: λ_{ex} = 405 nm, λ_{em} = 525/40 nm). Three independent experiments were performed and the standard deviation (SD) was calculated. An unpaired Student's t-test was performed for statistical analysis (*: p< 0.05; **: p< 0.01; ***; p< 0.001; ns: p$\geq$ 0.05).

Microscopy of 5 and 6 in A2780 and SBLF9

One day before the experiment, A2780 cells were seeded in RPMI 1640 medium containing 5% (v/v) FBS, 1% (v/v) L-Flu, and 1% (v/v) Pen/Strep (80 cells/µL, 0.5 mL) in imaging dishes (µ-Dish 35 mm, high, ibidi GmbH). SBLF9 were seeded in F-12 Medium supplemented with 15% (v/v) FBS, 2% (v/v) non-essential amino acids and 1% (v/v) Pen/Strep (40 cells/µL, 0.5 mL) in the same dishes. On the day of the experiment, the cells were incubated with **5** (2.5 µM, 1% DMSO) for 2 h at 37 °C and 5% CO_2. Subsequently, the cells were washed twice with 2 mL PBS, before Hoechst 33342 was added (1 µg/mL in 2 mL cultivation medium, 0.1% DMSO) and incubated for 20 min at 37 °C and 5 % CO_2. Cells were washed again twice with 2 mL PBS. Finally, 1 mL RPMI 1640 medium containing 5% (v/v) FBS, 1% (v/v) L-Glu, and 1% (v/v) Pen/Strep was added and fluorescent images taken with the Zeiss Axio Observer fluorescence microscope with a 40x/1.30 Oil DIC objective. Channel 1 (blue): λ_{ex}: 355 nm; λ_{em}: 450/50 nm; Channel 2 (green): λ_{ex}: 470/40 nm; λ_{em}: 525/50 nm. In addition, brightfield images (DIC) were taken.

Lysosomal Co-staining of 5 in A2780 and SBLF9

One day before the experiment, A2780 cells were seeded in RPMI 1640 medium containing 5% (v/v) FBS, 1% (v/v) L-Flu, and 1% (v/v) Pen/Strep (80 cells/µL, 0.5 mL) in imaging dishes (µ-Dish 35 mm, high, ibidi GmbH). SBLF9 were seeded in F-12 Medium supplemented with 15% (v/v) FBS, 2% (v/v) non-essential amino acids and 2% (v/v) Pen/Strep (40 cells/µL, 0.5 mL) in the same dishes. On the day of the experiment, the cells were incubated with 5 (2.5 µM, 1% DMSO) for 2 h at 37°C and 5% CO2 in RPMI 1640 medium containing 5% (v/v) FBS, 1% (v/v) L-Flu, and 1% (v/v) Pen/Strep (2 mL). Subsequently, the cells were washed twice with 2 mL PBS, before LysoTracker™ Deep Red (Thermo Fisher Scientific) was added (500 nM in 2 mL Hank's Balanced Salt Solution, 0.1% DMSO) and incubated for 20 min at 37 °C and 5 % CO2. Cells were washed again twice with 2 mL PBS. Finally, 1 mL RPMI 1640 medium containing 5% (v/v) FBS, 1% (v/v) L-Glu, and 1% (v/v) Pen/Strep was added and fluorescent images taken with the Zeiss Axio Observer fluorescence microscope with a 40x/1.30 Oil DIC objective. Channel 2 (green): λ_{ex}: 470/40 nm; λ_{em}: 525/50 nm; Channel 3 (red): λ_{ex}: 640/30 nm; λ_{em}: 690/50 nm. In addition, brightfield images (DIC) were taken. Pearson's Coefficients were calculated using ImageJ Fiji.

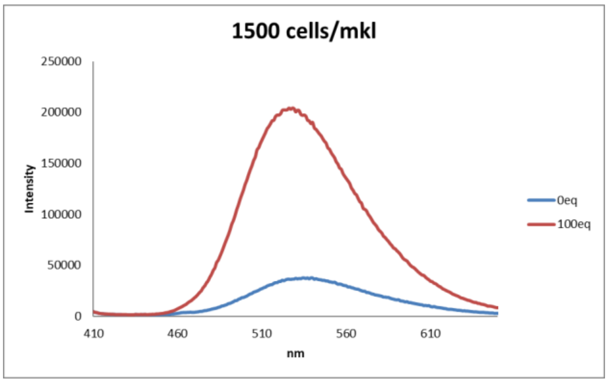

Figure S41. Detection of CTP in cell lysate 1500 cells/µL in a 50 ml MOPSO buffer, pH 6.2. The cell lysate was prepared by sonication of a solution with a desired concentration of cells for 1.5h at 4°C.

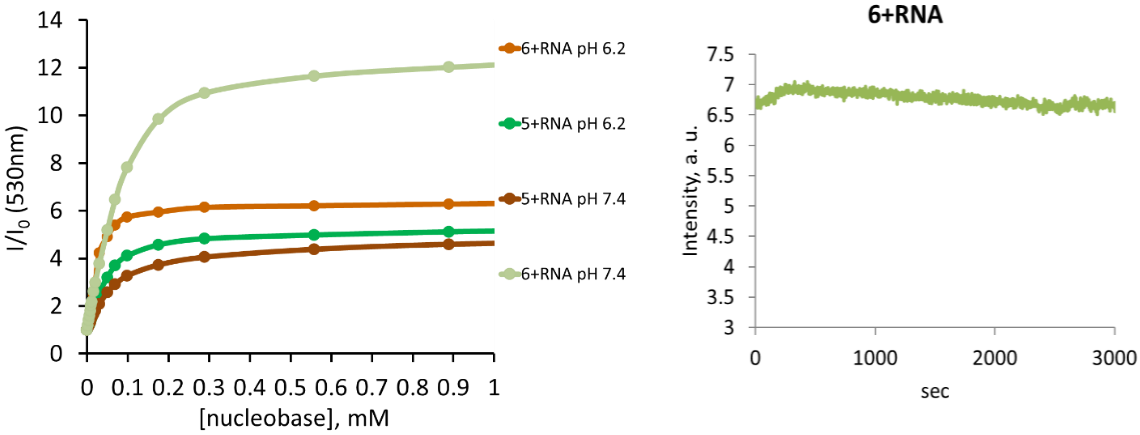

Figure S42. (Left figure) Comparison of fluorescence response of **5** and **6** with RNA at pH 7.4 in a 50 mM MOPSO buffer. (Right figure) Control experiment for the experiment shown in Figure 7d, in which **6** and RNA were incubated to show that no hydrolysis occurs in the absence of RNase. RNase was purchased from Sigma-Aldrich 70kU/mg. It was dissolved in a buffer and added to 2 ml cuvette to achieve concentration of 1mg/ml.

**Receptor 5**	**Receptor 6**
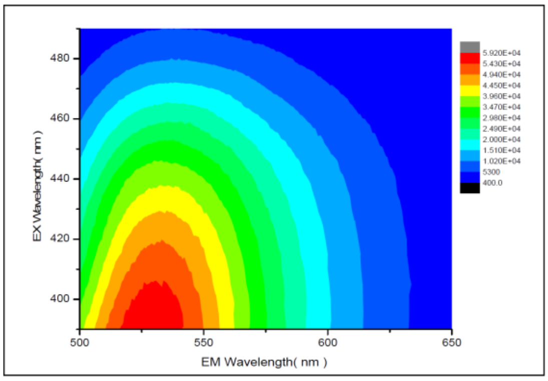	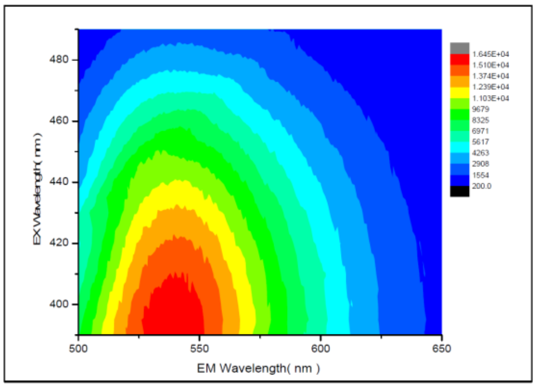
**Receptor 5 + RNA**	**Receptor 6 + RNA**

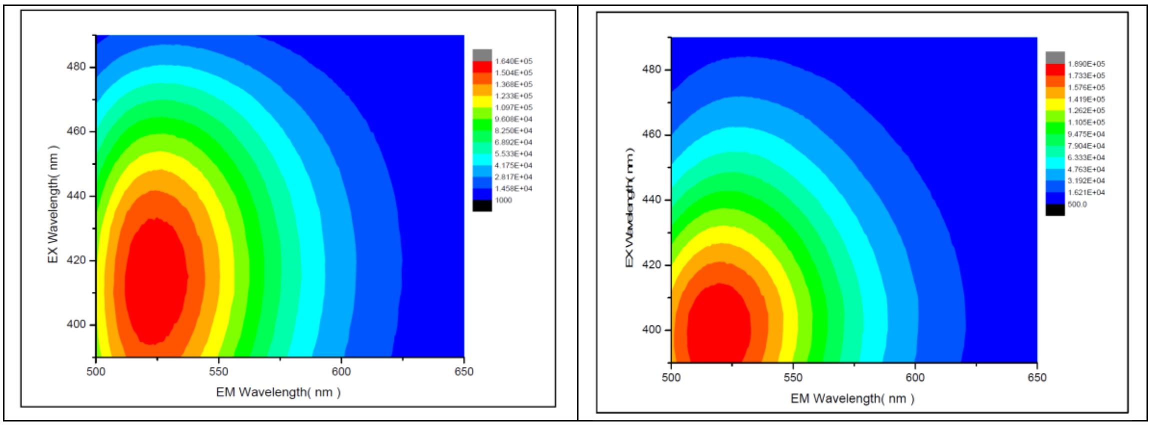

Figure S43. 2D excitation-emission scans for receptor 5 and 6 in the absence and in the presence of RNA.

Table S1. Apparent association constants for binding of RNA oligonucleotides determined in a 50 mM MOPSO pH 6.2.

AUCCCCU	$\log K_{21} = 11.98 \pm 0.02$; $\log K_{31} = 18.44 \pm 0.02$
AUCCCUU	$\log K_{21} = 12.82 \pm 0.01$
AUGUUGU	$\log K_{21} = 12.84 \pm 0.02$
AUGCUGC	$\log K_{21} = 12.80 \pm 0.05$
AUCCUCC	$\log K_{21} = 11.64 \pm 0.02$; $\log K_{31} = 18.60 \pm 0.02$
AUCCCUC	$\log K_{21} = 8.02 \pm 0.03$; $\log K_{31} = 17.12 \pm 0.02$
ACCUCUC	$\log K_{21} = 8.14 \pm 0.05$; $\log K_{31} = 17.45 \pm 0.02$

Figure S44.

DFT Calculations

The geometry of host obtained from X-ray data was used in the construction of host-guest complexes. Cytosine or CTP were placed together to generate 8 possible arrangements with or without π-π stacked naphthalimides and cytosine. The obtained structures were optimized by a parametrized model involving dispersion interactions.[6] Five most favorable structures were further optimized by more time-consuming DFT-D3 calculations by using software package ORCA. The coordinates of the complexes with lowest energies are presented below:

Complex **6**H_2^{2+}·Cytosine $^-$				**6**H_4^{4+}·CTP^4			
Energy = -2930.8327				-5128.4470			
	-4.079452000	-2.323687000	-0.867937000	8	-4.157523000	-3.062658000	0.582889000
8	-3.650397000	-0.049243000	-4.832287000	8	-4.198107000	-1.803520000	-3.836826000
8	-3.078442000	4.753194000	0.221389000	8	-3.740867000	4.147567000	0.931844000
8	-4.130727000	1.666648000	3.415372000	8	-4.626376000	1.160382000	4.288148000
7	-6.130378000	0.536692000	-1.800756000	7	-6.555433000	-0.590509000	-0.684662000
1	-6.827765000	1.297956000	-1.869862000	1	-7.378932000	0.021402000	-0.808263000
7	-5.554933000	1.359037000	1.117286000	7	-5.861342000	0.659210000	2.025349000
1	-5.379951000	1.325640000	2.162400000	1	-5.551111000	0.723920000	3.066698000
7	-3.846882000	-1.262937000	-2.901737000	7	-4.146392000	-2.411459000	-1.631127000
7	2.365735000	-2.039081000	-1.095574000	7	2.191941000	-2.942453000	-0.549384000
7	4.279675000	-4.207740000	-1.109046000	7	4.621768000	-4.527208000	-0.319120000
7	6.101228000	-2.128824000	0.144322000	7	5.982445000	-1.916968000	1.053793000
7	5.112000000	0.656480000	0.766310000	7	4.774654000	0.590837000	1.815735000
7	2.697361000	2.136003000	1.330908000	7	2.157438000	1.753238000	1.991128000
				7	-4.122793000	2.798359000	2.752961000

7	-3.575119000	3.378995000	1.993483000
6	-5.272683000	-1.443121000	-3.187276000
1	-5.371474000	-1.816539000	-4.226750000
1	-5.636951000	-2.225707000	-2.497028000
6	-6.132251000	-0.180493000	-3.131187000
1	-7.180055000	-0.453266000	-3.369226000
1	-5.769766000	0.549145000	-3.879639000
6	-6.413763000	-0.318018000	-0.603347000
1	-7.315345000	-0.927453000	-0.817042000
1	-5.557900000	-1.022733000	-0.496795000
6	-6.676997000	0.466117000	0.680199000
1	-6.854300000	-0.279494000	1.479680000
6	-5.680956000	2.821260000	0.759392000
1	-5.229339000	2.977574000	-0.239707000
1	-6.757548000	3.081013000	0.722785000
6	-4.990865000	3.702154000	1.799541000
1	-5.495842000	3.626443000	2.781553000
1	-5.039751000	4.747020000	1.439897000
6	-3.313061000	-1.882523000	-1.741194000
6	-1.859184000	-1.937624000	-1.648277000
6	-1.260394000	-2.642470000	-0.590229000
1	-1.914814000	-3.195171000	0.099200000
6	0.123170000	-2.682771000	-0.436239000
1	0.535962000	-3.322699000	0.351759000
6	1.008781000	-1.993123000	-1.314611000
6	0.405898000	-1.184103000	-2.379138000
6	1.126919000	-0.242829000	-3.166143000
1	2.189075000	-0.062374000	-2.959384000
6	0.516421000	0.499696000	-4.181922000
1	1.116075000	1.204599000	-4.776373000
6	-0.856157000	0.336715000	-4.453064000
1	-1.355824000	0.874065000	-5.272858000
6	-1.624223000	-0.497198000	-3.633505000
6	-1.024897000	-1.239096000	-2.570666000
6	-3.086315000	-0.571162000	-3.872501000
6	3.391885000	-2.104176000	-2.154938000
1	2.990381000	-1.750979000	-3.117084000
1	4.251573000	-1.475255000	-1.842004000
6	3.842012000	-3.579534000	-2.328967000
1	4.650197000	-3.620095000	-3.085518000
1	2.968544000	-4.136521000	-2.747879000
6	3.332090000	-4.075924000	-0.025474000
1	2.426276000	-4.694856000	-0.228321000
1	3.780737000	-4.445832000	0.917757000
6	2.911491000	-2.594841000	0.151148000
1	3.829047000	-2.013653000	0.367640000
1	2.189785000	-2.455981000	0.977451000
6	5.691556000	-4.372813000	-0.805469000
1	5.897021000	-5.425704000	-0.505058000
1	6.259586000	-4.205919000	-1.744158000
6	6.270806000	-3.462147000	0.274158000
6	6.633837000	-1.313173000	1.078401000
6	7.345402000	-1.802675000	2.192341000
1	7.758771000	-1.103153000	2.933913000
6	7.520499000	-3.185887000	2.331487000
1	8.072888000	-3.599249000	3.188715000
6	6.973962000	-4.029909000	1.356461000
1	7.090122000	-5.122006000	1.425221000
6	6.493716000	0.184477000	0.878613000
1	7.013421000	0.460779000	-0.064135000
1	7.037508000	0.703402000	1.708783000
6	5.063597000	2.101487000	0.580144000
1	5.467846000	2.643894000	1.476280000
1	5.701138000	2.383500000	-0.282430000
6	3.643519000	2.597532000	0.305528000
1	3.310951000	2.242967000	-0.700897000
1	3.658292000	3.703171000	0.294715000
6	2.820899000	0.677843000	1.543151000
1	2.124141000	0.342646000	2.327996000
1	2.552747000	0.163784000	0.588131000
6	4.255440000	0.289899000	1.885530000
1	4.307374000	-0.804195000	2.050578000
6	-5.570973000	-2.702855000	-1.755827000
1	-5.717424000	-3.275456000	-2.694119000
1	-5.846563000	-3.341775000	-0.897011000
6	-6.489459000	-1.488086000	-1.892167000
1	-7.515516000	-1.853807000	-2.105690000
1	-6.145718000	-0.867275000	-2.741908000
6	-6.645524000	-1.299506000	0.622493000
1	-7.472593000	-2.039197000	0.572239000
1	-5.700349000	-1.876420000	0.756811000
6	-6.908367000	-0.378369000	1.806255000
1	-6.957195000	-1.018116000	2.709789000
6	-6.208771000	2.069976000	1.621151000
1	-5.855548000	2.232494000	0.584311000
1	-7.310866000	2.187357000	1.657624000
6	-5.549608000	3.063472000	2.577657000
1	-6.028123000	3.028476000	3.575223000
1	-5.638383000	4.075254000	2.139897000
6	-3.487249000	-2.766533000	-0.428948000
6	-2.032851000	-2.761891000	-0.472191000
6	-1.298981000	-3.170494000	0.655899000
1	-1.858966000	-3.528955000	1.531089000
6	0.096955000	-3.169653000	0.654680000
1	0.630385000	-3.584756000	1.521609000
6	0.830477000	-2.772488000	-0.498187000
6	0.104182000	-2.206632000	-1.619602000
6	0.747733000	-1.482955000	-2.662062000
1	1.804260000	-1.203532000	-2.561707000
6	0.026041000	-1.029115000	-3.770299000
1	0.535648000	-0.405007000	-4.516541000
6	-1.361645000	-1.239383000	-3.843812000
1	-1.956184000	-0.866003000	-4.689594000
6	-2.046317000	-1.826317000	-2.767161000
6	-1.331585000	-2.289495000	-1.624756000
6	-3.504152000	-1.989066000	-2.836093000
6	2.972371000	-3.233791000	-1.750669000
1	2.303610000	-3.374844000	-2.618080000
1	3.712404000	-2.419416000	-2.000064000
6	3.741708000	-4.544992000	-1.563018000
1	4.410580000	-4.713877000	-2.428082000
1	3.061268000	-5.413425000	-1.438745000
6	3.786066000	-4.156546000	0.887043000
1	3.096332000	-5.010343000	1.051222000
1	4.471890000	-4.063541000	1.749133000
6	3.039324000	-2.859646000	0.630736000
1	3.764187000	-2.029720000	0.435649000
1	2.444431000	-2.598782000	1.522580000
6	5.921383000	-3.720482000	-0.537118000
1	6.619961000	-4.445462000	-0.996488000
1	5.707813000	-2.874739000	-1.286868000
6	6.509525000	-3.107064000	0.705900000
6	6.569921000	-1.173464000	2.002327000
6	7.667279000	-1.668370000	2.744818000
1	8.118730000	-1.052619000	3.537519000
6	8.190114000	-2.928478000	2.429323000
1	9.052247000	-3.329815000	2.983657000
6	7.624125000	-3.656224000	1.368125000
1	8.050910000	-4.620562000	1.051135000
6	6.167280000	0.280393000	2.103170000
1	6.509181000	0.710090000	3.079077000
6	4.572902000	2.032178000	1.596690000
1	4.786769000	2.598650000	2.540005000
1	5.243686000	2.364307000	0.777591000
6	3.163939000	2.329965000	1.095308000
1	3.048010000	1.937148000	0.056830000
1	3.035792000	3.423867000	1.039785000
6	2.387145000	0.306903000	2.153678000
1	1.606026000	-0.144724000	2.788808000
1	2.331284000	-0.139215000	1.133746000
6	3.782238000	0.040996000	2.729330000
1	3.944003000	-1.053344000	2.821985000
1	3.862370000	0.484046000	3.758633000
6	0.903062000	2.301894000	2.070142000
6	0.452902000	3.236844000	1.097487000
1	1.099276000	3.509938000	0.252273000
6	-0.878423000	3.639165000	1.063201000
1	-1.242001000	4.250780000	0.226492000

1	4.561519000	0.785269000	2.846316000
6	1.475840000	2.770147000	1.447416000
6	1.062742000	3.748210000	0.505521000
1	1.763982000	4.117930000	-0.251717000
6	-0.262915000	4.194302000	0.452838000
1	-0.576916000	4.916073000	-0.316046000
6	-1.240794000	3.670902000	1.309658000
6	-2.646857000	4.017263000	1.105055000
6	-3.242422000	2.400694000	2.929083000
6	-1.812673000	2.249770000	3.253455000
6	-0.841955000	2.794032000	2.357362000
6	0.541047000	2.425784000	2.512188000
6	0.914658000	1.750970000	3.705630000
1	1.981348000	1.608995000	3.929323000
6	-0.042284000	1.311847000	4.626675000
1	0.278541000	0.818983000	5.555850000
6	-1.415348000	1.500706000	4.366780000
1	-2.186037000	1.101363000	5.042316000
8	-3.876256000	1.836423000	-1.725599000
7	-3.082137000	0.709417000	0.113805000
6	-2.012547000	0.191311000	0.765156000
6	-0.677877000	0.454445000	0.323298000
7	-1.619249000	1.720857000	-1.438265000
6	-0.521849000	1.240991000	-0.785659000
6	-2.911200000	1.427595000	-1.030010000
7	-2.237509000	-0.565980000	1.851313000
1	-1.459578000	-0.894234000	2.420882000
1	-3.181200000	-0.690203000	2.216766000
1	-1.524919000	2.247848000	-2.311992000
1	0.454229000	1.509002000	-1.202437000
1	0.186948000	0.038850000	0.846166000
1	-4.591951000	0.996362000	0.721871000
1	-5.155835000	1.054315000	-1.699550000
1	-7.598031000	1.081331000	0.612667000

6	-1.827894000	3.144730000	1.989642000
6	-3.236474000	3.445500000	1.813235000
6	-3.743650000	1.878886000	3.724644000
6	-2.320780000	1.741801000	3.985127000
6	-1.393503000	2.293645000	3.042794000
6	-0.004811000	1.925599000	3.145681000
6	0.418245000	1.235434000	4.311888000
1	1.494841000	1.098425000	4.483855000
6	-0.500137000	0.765251000	5.264569000
1	-0.136642000	0.242627000	6.162045000
6	-1.875577000	0.966170000	5.073260000
1	-2.623911000	0.556497000	5.766891000
8	-4.769265000	1.167146000	-1.011569000
7	-3.501497000	0.255754000	0.654455000
6	-2.270444000	-0.081931000	1.121754000
6	-1.103748000	0.289631000	0.404063000
7	-2.496648000	1.521084000	-1.086131000
6	-1.247913000	1.140599000	-0.665179000
6	-3.619648000	0.974489000	-0.489306000
7	-2.210619000	-0.782586000	2.273414000
1	-1.317193000	-0.883645000	2.751897000
1	-3.057354000	-0.950226000	2.813497000
1	-0.346508000	1.508136000	-1.196010000
1	-0.098369000	-0.029910000	0.696878000
1	-4.921024000	0.386450000	1.510595000
1	-5.668851000	0.172533000	-0.747525000
1	-7.891542000	0.131958000	1.725279000
6	-1.624206000	3.180363000	-4.270051000
6	-2.154969000	1.926907000	-3.550751000
6	-1.260232000	4.146135000	-3.124567000
6	-2.658467000	2.493942000	-2.205162000
8	-1.896644000	3.632916000	-1.923483000
8	-2.707623000	3.693416000	-5.074541000
8	-3.135388000	1.243148000	-4.283695000
1	-3.747273000	2.701679000	-2.248509000
1	-1.277137000	1.274351000	-3.368812000
1	-0.733103000	2.904164000	-4.867505000
6	0.233229000	4.401179000	-2.833052000
1	0.761775000	4.570546000	-3.798670000
1	0.310853000	5.340313000	-2.243737000
8	0.835177000	3.390595000	-2.061309000
15	1.569639000	2.036229000	-2.781305000
8	1.567098000	1.054450000	-1.605626000
8	0.805459000	1.731221000	-4.068630000
1	4.918675000	-5.501293000	-0.157118000
8	3.020422000	2.612900000	-3.220231000
15	4.597617000	2.470306000	-2.610483000
8	4.575818000	2.899014000	-1.147121000
8	5.493230000	3.146394000	-3.615413000
8	4.728553000	0.803669000	-2.751818000
15	5.295043000	-0.257095000	-1.619576000
8	4.009528000	-0.366278000	-0.573187000
8	5.329639000	-1.621241000	-2.358408000
8	6.517611000	0.248368000	-0.873062000
1	-3.490988000	1.945479000	-4.880490000
1	-2.337602000	3.924763000	-5.944150000
1	-1.733165000	5.123057000	-3.373995000
1	4.363632000	-0.006604000	0.305933000
1	6.738556000	0.747833000	1.263089000

References

1. Gans, P.; Sabatini, A.; Vacca, A., Investigation of equilibria in solution. Determination of equilibrium constants with the HYPERQUAD suite of programs. *Talanta* **1996,** *43*, 1739-1753.

2. Fernandez-Alonso, S.; Corrales, T.; Pablos, J. L.; Catalina, F., A Switchable fluorescence solid sensor for Hg2+ detection in aqueous media based on a photocrosslinked membrane functionalized with (benzimidazolyl)methyl-piperazine derivative of 1,8-naphthalimide. *Sensor Actuat B-Chem* **2018,** *270*, 256-262.

3. Zhang, Z. Q.; Feng, Q.; Yang, M.; Tang, Y. L., A ratiometric fluorescent biosensor based on conjugated polymers for sensitive detection of nitroreductase and hypoxia diagnosis in tumor cells. *Sensor Actuat B-Chem* **2020,** *318*.
4. Mei, Q. B.; Shi, Y. J.; Hua, Q. F.; Tong, B. H., Phosphorescent chemosensor for Hg2+ based on an iridium(III) complex coordinated with 4-phenylquinazoline and carbazole dithiocarbamate. *Rsc Advances* **2015,** *5* (91), 74924-74931.
5. Weigert, V.; Jost, T.; Hecht, M.; Knippertz, I.; Heinzerling, L.; Fietkau, R.; Distel, L. V., PARP inhibitors combined with ionizing radiation induce different effects in melanoma cells and healthy fibroblasts. *BMC Cancer* **2020,** *20* (1), 775.
6. Laikov, D. N., A new parametrizable model of molecular electronic structure. *J Chem Phys* **2011,** *135* (13), 134120.
